# Psychological and computational evidence that merit is a constructed evaluative process

**DOI:** 10.1126/sciadv.aeh5472

**Published:** 2026-10-02

**Authors:** Sarah Vahed, Alan G. Sanfey

**Affiliations:** ^1^ Donders Institute for Brain, Cognition and Behaviour, Radboud University, Netherlands.; ^2^ Behavioural Science Institute, Radboud University, Netherlands.

## Abstract

Merit is a foundational moral principle invoked to justify inequality between people, yet an individual’s productivity is shaped not only by their efforts but also their opportunities. How, then, do people construct merit judgements? Across four preregistered, incentivized studies with US-based samples (total *N* = 759), we provide psychological and computational evidence that merit is not the product of a fixed rule, but rather the outcome of a subjective valuation. In a two-stage paradigm, participants first completed a real-effort tournament that dissociated performance (effort) from structural productivity (output dependent on opportunity). Thereafter, groups of observers and stakeholders allocated resources to tournament players. Across studies, a player’s performance and structural productivity influenced how people judged deservingness. Computational modeling revealed that decision-makers integrated these dimensions of merit into a value computation that shaped allocations. Crucially, individuals differed in their latent weighting patterns, yielding subgroups with directly opposing conceptions of deservingness. By revealing merit as a nonuniform ideal, these findings provide a mechanistic account of how deservingness is determined, helping explain the compelling yet contested nature of meritocracy.

## INTRODUCTION

Contemporary debates about social inequality hinge upon judgement of who is deemed as “deserving” in society (*1*). A dominant ideal invoked to guide distributive decisions is that of merit, which asserts that resources and opportunities should be allocated in proportion to an individual’s contributions or achievements (*2*–*8*). Across social, political, and economic domains, merit-based systems shape societies’ most consequential allocations. University admissions, hiring decisions, and allocations of grants or bonuses are all routinely justified on the basis of individual performance or productivity (*5*, *9*–*11*). As such, merit functions as a moral filter, with unequal outcomes derived from meritocratic attributes perceived as fair, whereas unequal outcomes attributed to chance, arbitrariness, or corruption are not (*5*, *6*, *12*–*14*). In this way, merit not only determines allocations, it shapes the standard by which fairness itself is evaluated. Understanding how people evaluate merit is therefore central to questions of justice, revealing when and why individuals choose to tolerate or contest social disparities. Here, we introduce an experimental and computational framework that formalizes how people construct judgements of merit, and then use their conceptualization of this foundational principle to determine who they consider to be deserving.

Traditionally, the notion of merit has been conceived as a self-evident and unitary property of individuals, most often operationalized through observable performance, and frequently contrasted with luck (*4*, *15*–*18*). Under this assumption, deservingness follows directly from output: Higher performers are more productive and therefore more deserving, lower performers less so. This framing aligns with the ideal of meritocracy as a principled and bias-free system, in which only relevant inputs (such as amount of effort invested or tangible outcomes produced) are considered, while unrelated characteristics are deemed irrelevant, and are thus excluded (*7*, *12*, *19*). Although this approach has engendered a substantial body of literature on equity and merit-based allocation (*14*, *19*, *20*), it has left a crucial question underpinning distributive justice unresolved, namely, how people construct deservingness judgements.

A central reason the formative question has remained unexamined is the widespread implicit assumption that performance and productivity are interchangeable, that is, how much someone produces faithfully reflects how much effort they put in. In many real-world systems, however, this supposition fails. Two individuals may both exert effort yet produce dissimilar outputs, not necessarily because of differences in persistence or ability, but instead due to the conditions under which they act (*19*, *21*). Performance and productivity can thus come into conflict, with differences in output in fact reflecting unequal opportunities. This tension lies at the core of criticism against modern merit-based systems as being “flawed” or a “myth” (*3*, *21*–*23*), not due to merit being irrelevant, but because the signals used to infer merit may be systematically distorted by noncomparable external conditions between individuals (*2*, *21*, *22*). Yet, despite this critique, little is empirically known about how people assign merit under such conditions. In other words, when the basic assumption that performance equals productivity is violated, how do we evaluate merit?

In the present research, we investigate this gap by examining how individuals determine deservingness. We distinguish two dimensions of merit: performance, defined as how much effort an individual exerts relative to others, and productivity, defined as the amount of output delivered. Productivity here does not depend on effort alone but also on the structural conditions that govern how efficiently effort can be converted into output. For example, in a running race, all else being equal in ability and preparation between athletes, differences in who finishes ahead are not determined by effort alone, but also by the conditions under which runners compete, such as how close to the finish line they are placed relative to one another and how many hurdles obstruct the route for each athlete. We refer to this mapping between effort and output as structural productivity, which captures underlying differences in opportunity to produce. To isolate these components and allow for an exploration of how they may be differentially used in assessments of deservingness, we designed a two-stage paradigm. First, players in a tournament setting performed an effortful task (namely, counting grids of zeros and ones), but differed in experimentally manipulated task difficulty. This manipulation altered the efficiency with which some individuals could generate output, without changing the underlying action across players, allowing productivity differences to arise from opportunities embedded in the task structure rather than from differential exertion or talent. Second, we then examined how people allocated resources to tournament players, including in situations varying in the decision-maker’s stake and proximity to the system, helping us examine patterns of deservingness judgements across distinct decision contexts (Fig. 1).

**Fig. 1.**
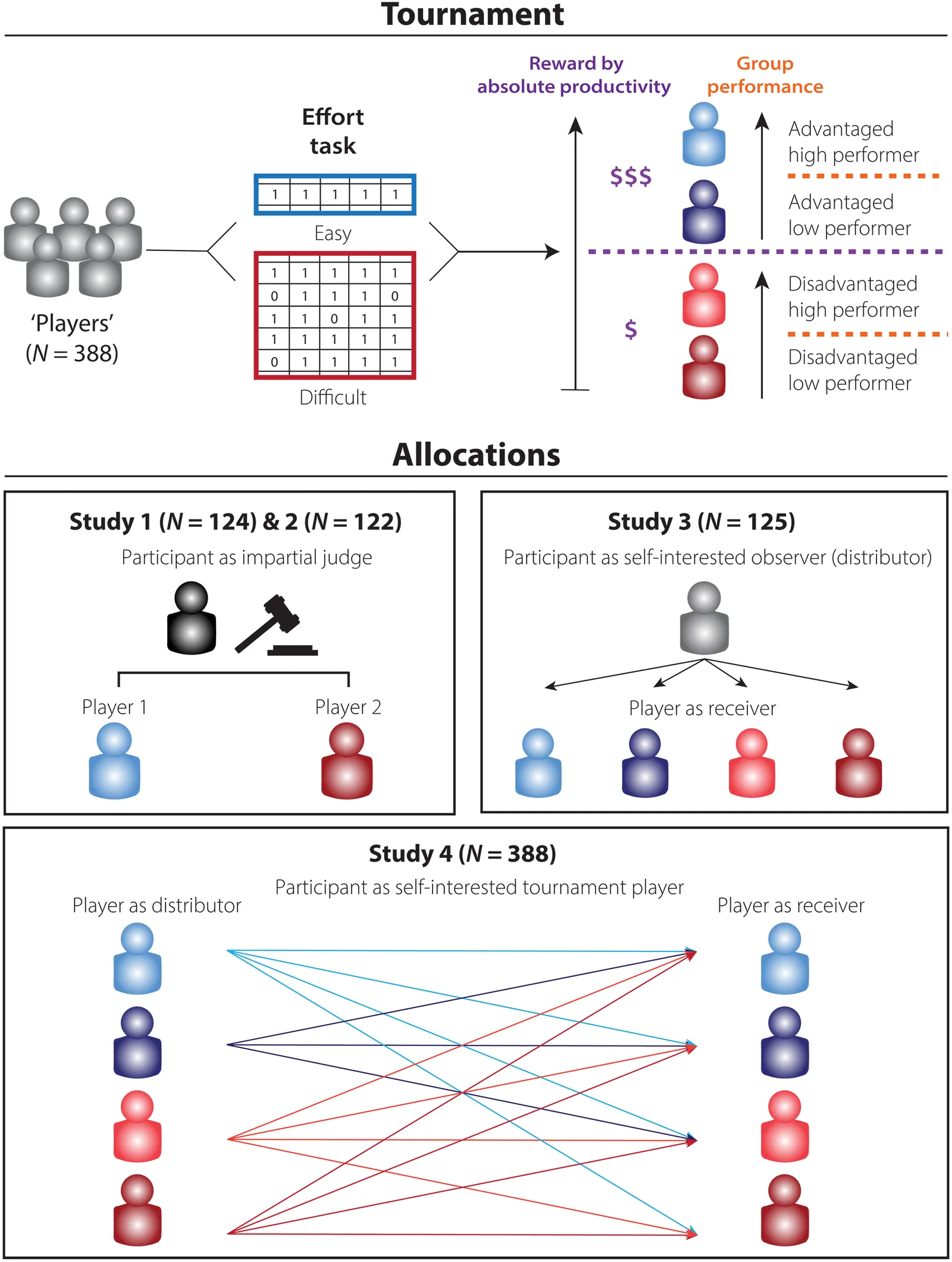
Schematic of study designs. Tournament. Players (*N* = 388) completed a real-effort task in which they were presented with tables comprising of zeros and ones. For each table, players were required to correctly count and input the number of zeros in that table within two attempts and within a maximum of 8 s (across three blocks of 3 min). In a between-subjects manipulation, participants were presented either easy or difficult tables. Absolute productivity was used to determine high or low reward outcomes. Given the structural opportunities embedded in the task, participants who completed the easy tables were the most productive and thereby received the high reward, while those who completed the difficult tables were less productive and received the low reward (structural productivity). Within-group performance, high or low, indexed number of tables completed compared to others facing the same task difficulty (performance). Allocations. Across four studies, participants were provided the opportunity to allocate additional resources to tournament players, though distributive role varied: studies 1 and 2 (impartial judges; *N* = 246): Participants divided a $1 endowment between two tournament players while viewing the tournament metrics (tables completed, group performance, and overall performance/reward) of both players. Study 3 (self-interested observer; *N* = 125): Participants completed a modified Dictator Game (DG) during which a single tournament player was presented at a time. They could share a $1 endowment between themselves and that player. Study 4 (self-interested tournament player; *N* = 388): Participants had themselves completed the tournament and undertook a modified DG in which they viewed both their own tournament metrics and those of another player, making $1 allocation decisions.

Across four preregistered and incentivized studies, samples of US-based participants allocated resources in impartial contexts (studies 1 and 2: *N* = 246), situations involving self-interest (study 3: *N* = 125), and scenarios where participants directly experienced the (unequal) system themselves (study 4: *N* = 388). Combining behavioral analyses with computational modeling, we investigated the degree to which resource allocation choices are sensitive to cues of performance and structural productivity, as well as the joint influence of these merit dimensions in guiding distributive decision-making. Our analytic approach focused on the individual-level to uncover whether allocations follow a single universal principle or instead reveal patterns of systematic differences across people in who they consider to be deserving. By treating merit as a constructed value that may differ across individuals, rather than an assumed ideal shared by all, this work provides a principled account of how deservingness is conceptualized. In doing so, we move beyond simply asking whether people endorse meritocratic beliefs to specifying how merit itself is computationally formed and applied.

## RESULTS

### Tournament

#### Structural opportunity biases productivity, perceived control, and fairness

To examine how performance and structural productivity influence allocation decisions, we first implemented a real-effort tournament, in which a sample of players was randomly assigned to either advantaged or disadvantaged conditions. Although all players performed the same underlying activity (i.e., counting zeros), the difficulty of the task differed, creating inequality in opportunity to generate rewards based on absolute productivity independent of effort, thus allowing us to dissociate structural productivity from performance (see Materials and Methods).

Players’ absolute productivity was indexed by their number of correctly completed tables. Across the sample (*n* = 388), the median number of completed tables was 61 (range: 0 to 283). Players assigned to the “difficult” tables completed on average 17 (range: 0 to 60), whereas players assigned the “easy” tables completed 178 tables (range: 62 to 283). Within the disadvantaged condition, low performers completed an average of five tables (range: 0 to 13) and high performers completed 31 (range: 14 to 60). Within the advantaged condition, low performers completed 132 tables on average (range: 62 to 173) and high performers 217 tables (range: 175 to 283) (Fig. 2). Thus, while performance differences were present within each condition, productivity was determined by task difficulty, rather than pure effort alone.

**Fig. 2.**
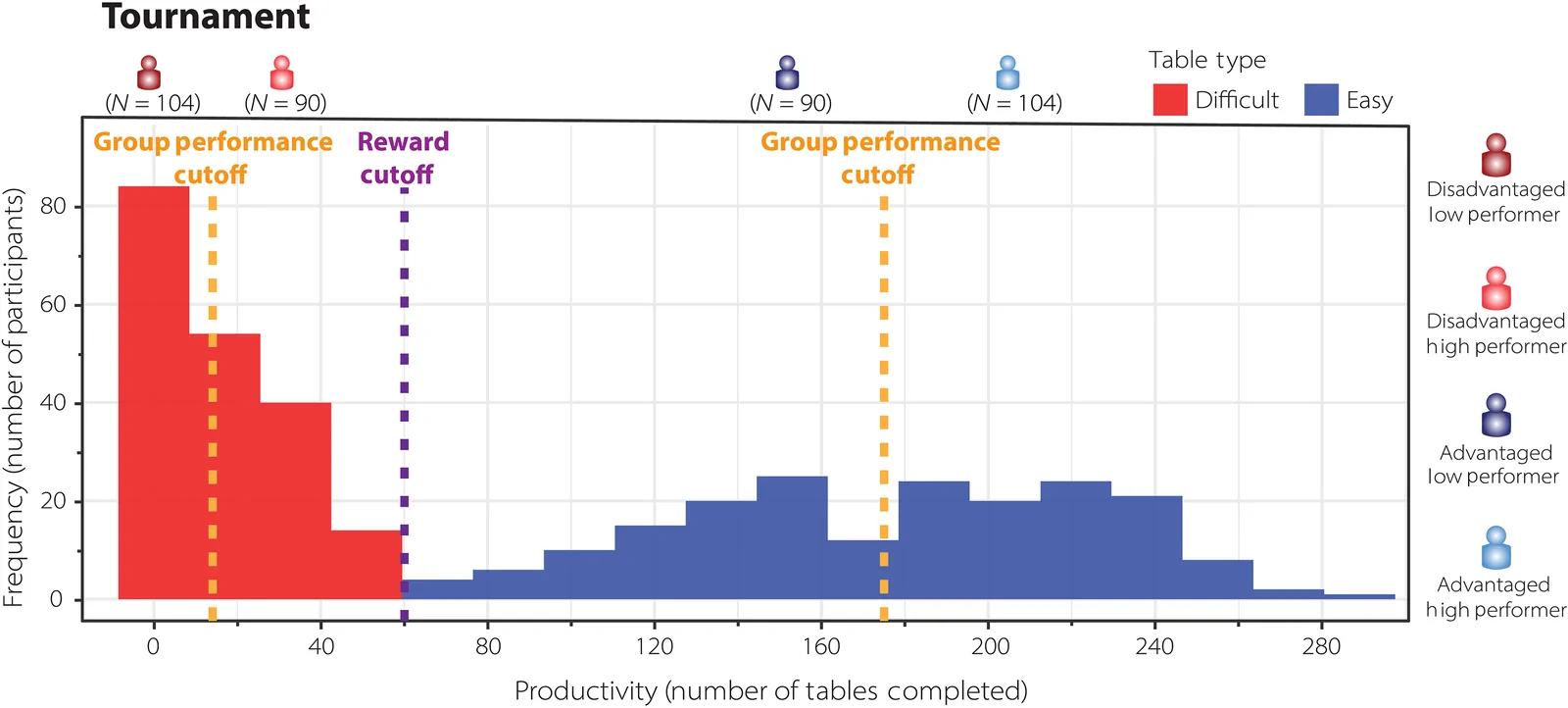
Tournament summary. Histogram of productivity distribution (number of correctly completed tables) by assigned table type (red = difficult, blue = easy). Bars represent the frequency of participants across productivity bins (used for purposes of distribution visualization only). Yellow dotted lines mark within-condition thresholds separating low versus high performers, while purple dotted line indicates median split of the sample used to assign low versus high reward levels.

To confirm whether the manipulation altered subjective experience beyond behavioral differences in output, we assessed perceptions of control, effort, luck, and fairness [see the Supplementary Materials (Questionnaires)]. Participants in the disadvantaged condition reported significantly lower perceived control over the tournament outcome (*M*_disadv_ = 1.95 out of 5) than participants in the advantaged condition (*M*_adv_ = 3.02; *P* < 0.001). Similarly, in contrast with those advantaged, disadvantaged players attributed outcomes more to luck than effort (*M*_disadv_ = 42% out of 100 versus *M*_adv_ = 61%; *P* < 0.001) and rated the tournament as significantly unfair (*M*_disadv_ = 1.84 of 5 versus *M*_adv_ = 3.18; *P* < 0.001). To assess whether participants experienced the tournament as requiring similar effort but yielding different rewards across conditions, we computed an effort-reward ratio, which captures how much effort participants felt they invested relative to the rewards they received. Participants in the disadvantaged condition reported a significantly higher effort-reward imbalance (*M*_disadv_ = 1.35) than participants in the advantaged condition (*M*_adv_ = 0.93; *P* < 0.001; see Supplementary Results S5 for full statistics). Together, these findings indicate that structural disadvantage reduced absolute productivity and perceived control, shifted outcome attributions toward luck, increased perceptions of unfairness and resulted in greater effort-reward imbalance.

### Allocations

#### Studies 1 and 2: Structural productivity and performance shape third-party allocations

Having experimentally induced a flawed meritocratic system, we sought to examine whether the structural productivity and performance of players influenced merit judgements. In study 1 (*N* = 124), participants acted as impartial judges and divided a $1 reward between two tournament players while presented with information about how each player had performed (the tournament metrics) (See Materials and Methods).

For pairs of players with similar tournament metrics, participants divided the $1 equally, giving both players $0.50 on average. However, for mixed pairs (i.e., differences in productivity and performance levels), on average, participants deviated from 50/50 allocations (Fig. 3A). A generalized linear mixed-effects model with a beta distribution and logit link function, using relative-difference coding of player metrics, revealed significant main effects of structural productivity (H1: β = 0.49, SE = 0.03, *z* = 15.56, *P* < 0.001) and performance (H2: β = 0.87, SE = 0.04, *z* = 21.46, *P* < 0.001), with participants allocating more to player 2 when they were disadvantaged and when they were a higher performer. A significant interaction (H3: β = −0.12, SE = 0.05, *z* = −2.41, *P* = 0.016) indicated that the performance advantage was attenuated when the player was disadvantaged. Main effects were robust to a fully specified model separating players 1 and 2 attributes (table S5). A mixed-effects logistic model predicting equal allocations ($0.45 to $0.55) further showed that participants were substantially more likely to split equally when players matched on productivity and performance levels (H4: β = 4.56, SE = 0.12, *z* = 38.31, *P* < 0.001).

**Fig. 3.**
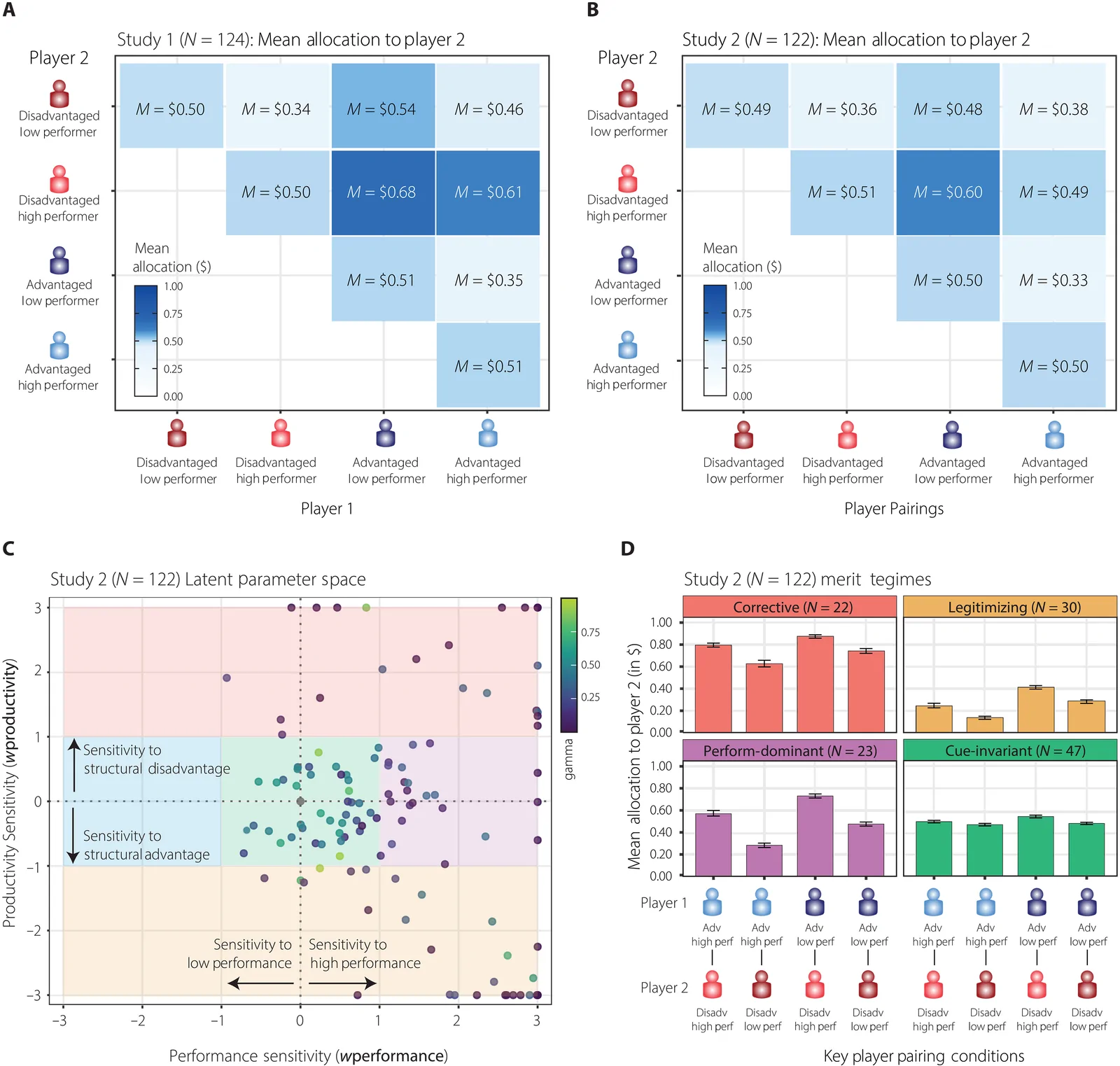
Allocations in studies 1 and 2. (**A**) Study 1 judgement task. Heatmap of the mean proportional allocation to player 2 (with remainder allocated to player 1), as a function of player 1 and player 2 pairing. Tile color indicates the average allocation (out of $1), with darker colors representing higher allocations. (**B**) Study 2 judgement task. Heatmap of the mean proportional allocation to player 2 (with remainder allocated to player 1), as a function of player 1 versus player 2 pairing. Tile color indicates the average allocation (out of $1), with darker colors representing higher allocations. (**C**) Latent parameter space. Participants’ estimated raw parameters, plotted as sensitivity to performance (*w*_performance_; *x* axis) and sensitivity to structural productivity (*w*_productivity_; *y* axis). *X*-axis values >0 reflect sensitivity to high performance players while values <0 reflect sensitivity to low performance players. *Y*-axis values >0 reflect sensitivity to structurally disadvantaged players and values <0 reflects sensitivity to structurally advantaged players. Vertical and horizontal dashed lines at 0 indicate null sensitivity. Shaded regions delineate relative weighing of performance versus structural productivity, defining distinct merit regimes (red = corrective; yellow = legitimizing; purple = performance-dominant; green = cue-invariant; blue = performance-lenient). Point color reflects individual-level sensitivity attenuation (auxiliary parameter: gamma, γ). (**D**) Merit regimes. Mean proportional allocation to player 2 (out of $1; remainder allocated to player 1) across mixed player pairings, grouped by cluster-derived latent sensitivities. Error bars reflect ±1 SEM.

Given the observed effect of structural productivity, we sought to investigate whether this result is driven by knowledge of the reward amounts (i.e., $0.19/$3.00). To rule out anchoring on payout values, we replicated the task with study 2 (*N* = 122), with participants here only informed that the tournament involved a reward disparity between players, without any specific monetary amounts mentioned other than the terms “high” or “low” reward. The pattern of results followed study 1 (Fig. 3B). A generalized linear mixed-effects model with a beta distribution and logit link revealed significant effects of structural productivity (H1: β = −0.14, SE = 0.03, *z* = −4.13, *P* < 0.001) and performance (H2: β = −0.91, SE = 0.03, *z* = −26.29, *P* < 0.001), as well as significant interaction (H3: β = 0.14, SE = 0.05, *z* = 2.84, *P* = 0.005), indicating that the penalty for low performance was modestly weakened when the player was also disadvantaged (table S6). A generalized linear mixed-effects model with a binomial distribution predicting equal (50/50) allocations showed that participants were substantially more likely to split equally when players matched on productivity and performance levels (H4: β = 3.76, SE = 0.08, *z* = 49.06, *P* < 0.001).

#### Study 2: Latent integration of structural productivity and performance underlies allocations

Our behavioral findings demonstrated that both structural productivity and performance are weighted when forming judgements of merit. However, the results raised the question whether weighting is consistent across individuals, that is, does meritocratic decision-making reflect a shared collective principle, or rather vary as a function of individual (or perhaps subgroup) norms? To investigate this, we formalized participants’ trial-by-trial allocations using a set of computational models fitted to data from study 2. Model comparison indicated that decisions were most parsimoniously explained by a latent value-difference model in which participants jointly integrated the tournament players’ structural productivity and performance categories. In this model, participants were assumed to construct a subjective value difference signal that guided decisions. Subjective value on each trial was defined as

$$\Delta V=w_{\text{productivity}}.s_{\text{productivity}}+w_{\text{performance}}.s_{\text{performance}}$$ (1)

where *s*_productivity_ and *s*_performance_ encode trial-level contrasts between player 2 and player 1 in structural productivity and performance category, respectively, and *w*_productivity_ and *w*_performance_ capture individual sensitivity to each dimension. Positive values of *w*_productivity_ reflect preferences favoring structurally disadvantaged players, whereas positive values of *w*_performance_ reflect preferences that favor higher-performing players. Trial-wise value differences were mapped onto expected allocations using a sensitivity-attenuated logistic function,

$$\mu =\frac{1}{1+\text{exp}\left[-\left(1-\gamma \right)\Delta V\right]}$$ (2)

where μ ∈ (0,1) denotes expected proportion allocated to player 2 and γ ∈ (0,1) captures the extent to which value-differences translate into allocation choice (see Materials and Methods).

We compared this integrated value-difference model against three theoretically motivated alternatives: structural productivity-only, performance-only, and a null model corresponding to a cue-invariant heuristic (i.e., consistent 50/50 split). Group-level information-criterion values [Akaike information criterion (AIC) and Bayesian information criterion (BIC)] strongly favored the integrated model. Relative to the null model, the integrated model showed substantially lower AIC (mean ΔAIC = −2116; median ΔAIC = −346) and BIC (mean ΔBIC = −2109; median ΔBIC = −339), indicating a better fit. Similar advantages were observed relative to the productivity-only model (mean ΔAIC = −1272; median ΔAIC = −55.2; mean ΔBIC = −1267; median ΔBIC = −50.5) and the performance-only model [mean ΔAIC = −1245; median ΔAIC = −171; mean ΔBIC = −1240; median ΔBIC = −166; table S7 (Model comparison)]. Parameter recovery tests further indicated that the integrated model parameters were recoverable: structural productivity sensitivity [*r* = 0.87, 95% confidence interval CI (0.82 to 0.91), *t*(123) = 19.6, *P* < 0.001], and performance sensitivity [*r* = 0.86, 95% CI (0.80 to 0.90), *t*(123) = 18.4, *P* < 0.001]. Recovery of the auxiliary sensitivity-attenuation parameter, gamma, was weaker [*r* = 0.52, 95% CI (0.39 to 0.64), *t*(123) = 6.89, *P* < 0.001; fig. S1 (parameter recovery), robustness checks: figs. S2 (Simulations), S3 (Confusion matrix), Supplementary Methods: Held-out predictive performance, and S4 (Held-out predictive performance)].

While the integrated model best explained behavior, raw fitted parameters varied substantially across participants (Fig. 3C). Performance sensitivity was predominantly positive (*M* = 1.16), indicating a tendency to allocate more to higher-performing players, with estimates ranging from −0.93 to 3.00. Productivity sensitivity was more evenly distributed around zero (*M* = −0.20), yet spanned the parameter range [−3;3]. These results indicate that third-party meritocratic decisions arise from a latent evaluative process, with variation in dimension weightings across individuals.

#### Study 2: Individual differences reveal heterogeneous clusters of merit regimes

Having established that participants, on average, integrate both structural productivity and performance when forming distributive judgements, we turned to the question of whether there might be distinct, interpretable patterns in how these weightings are constructed across people. Although the integrated model explained behavior at the group level, estimated sensitivity parameters varied substantially across participants. To characterize this variation, we classified individuals into latent regimes (i.e. strategies) based on their raw parameters using a conservative magnitude threshold (∣*w*| ≥ 1; see Supplementary Methods). This parameter-based classification converged with allocation patterns generated from an independent k-means clustering of behavior [fig. S5 (*K*-means clustering)], supporting its validity.

Applying this classification, a substantial proportion of participants can be described as largely insensitive to tournament information (“null/cue-invariant:” 38.5% of sample, *N* = 47). Among participants exhibiting stronger sensitivities, 24.6% primarily favored advantaged over disadvantaged players (“legitimizing,” *N* = 30), 18.9% selectively weighted high performance irrespective of task condition (“performance-dominant,” *N* = 23), and 18% favored disadvantaged over advantaged players (“corrective,” *N* = 22). These regimes corresponded to distinct allocation patterns across tournament pairings (Fig. 3D), indicating that heterogeneity in parameter estimates reflects interpretable differences in behavior.

#### Studies 3 and 4: Structural productivity and performance weighting arise across decision contexts

Study 2 analyses focused on allocations made in the absence of a personal stake, with the decision-maker acting as an impartial judge. Nonetheless, real-world judgements of deservingness are frequently made by individuals who are not complete external arbiters, but also participants in the allocative system. Our results thus left open the question as to whether allocation and underlying weighting of structural productivity and performance are similarly observed when decisions occur in contexts in which personal stake and partiality are inherent in the choice process. To this end, we analyzed allocations, model-derived parameters and regimes in two studies that differ in participant role: one in which participants are self-interested, but neutral observers of the tournament (study 3), and one in which participants are themselves tournament players (study 4).

#### Study 3: Structural productivity and performance are weighted during self-interested allocations

To capture allocations in a context involving self-interest, participants in study 3 (*N* = 125) were assigned the role of tournament “observer,” and on each trial were paired with a tournament player. In a modified Dictator Game (DG), participants received a $1 endowment, and had the choice to share any amount with the paired player (see Materials and Methods).

On average, participants allocated $0.29. The highest allocations were given to disadvantaged high performers ($0.40), followed by advantaged high performers ($0.31), disadvantaged low performers ($0.26), with the lowest amounts allocated to advantaged low performers ($0.21) (Fig. 4A). A linear mixed-effects model revealed a significant main effect of receiver’s productivity. Specifically, participants allocated more to disadvantaged players compared to advantaged players [H1: β = 0.0907, SE = 0.0096, *t*(2872) = 9.397, *P* < 0.001]. A main effect of performance was also found, with participants allocating less to low performers compared to high performers [H2: β = −0.0996, SE = 0.0096, *t*(2872) = −10.322, *P* < 0.001], as well as a significant interaction between a player’s productivity and performance [H3: β = −0.0443, SE = 0.0137, *t*(2872) = −3.244, *P* = 0.001]. These results indicate that the difference in allocations based on performance was more pronounced for disadvantaged players. As a final trial, participants were paired with a receiver who had also “observed” the tournament. On average, participants allocated $0.36 (±0.03SE), confirming the sample aligns with typical DG allocation patterns (Fig. 4B).

**Fig. 4.**
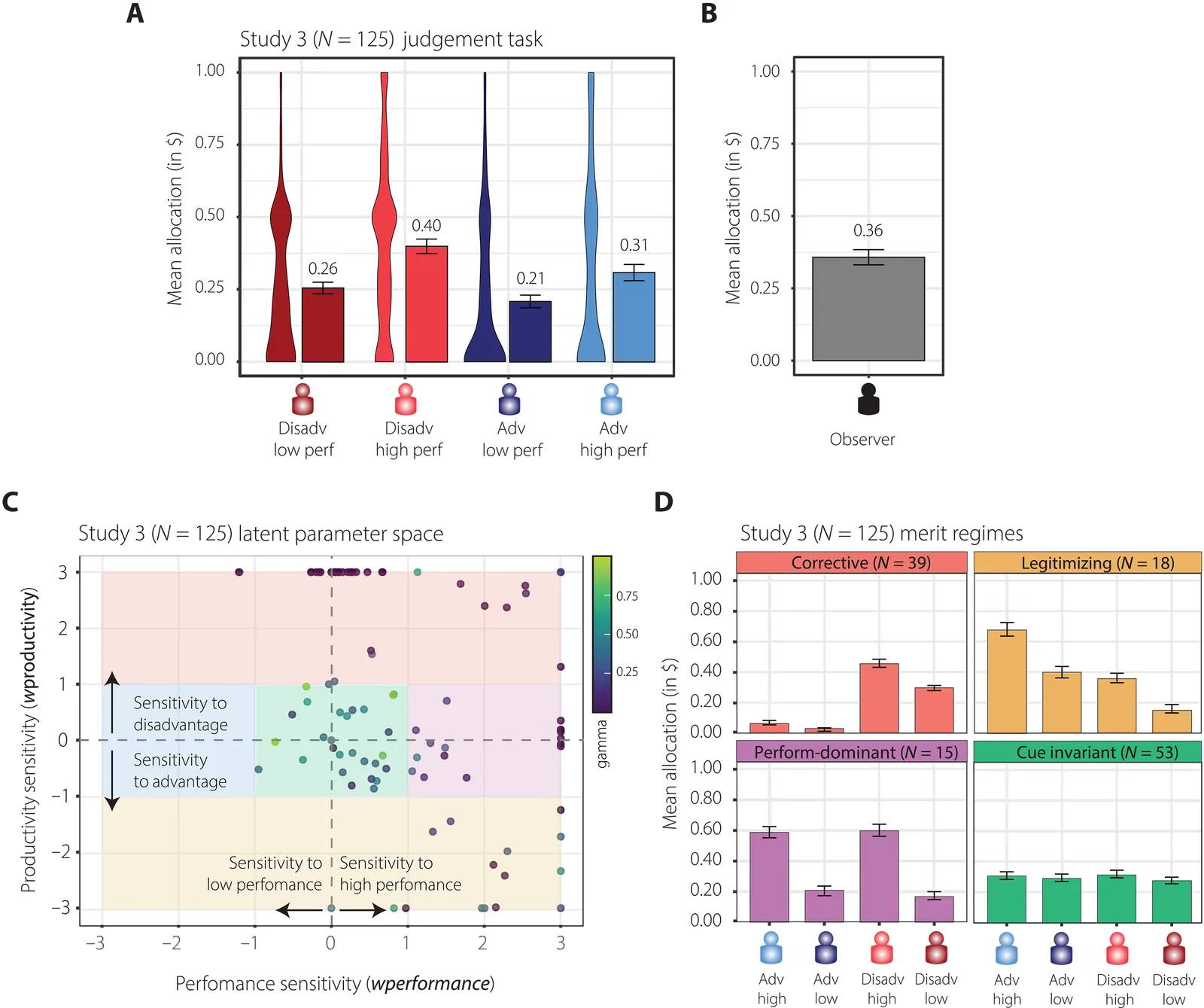
Allocations in study 3. (**A**) Study 3 judgement task (pairing with player). Mean allocation to the receiver as a function of receiver tournament group, with adjacent violin plots to illustrate response distribution. Error bars indicate ±1 SEM. (**B**) Study 3 judgement task (pairing with observer). Mean allocation to receiver who also participated in the role of observer. Error bar indicates ±1 SEM. (**C**) Latent parameter space. Each point represents an individual participant’s estimated raw parameters, plotted as sensitivity to performance (*w*_performance_; *x* axis) and sensitivity to structural productivity (*w*_productivity_; *y* axis). Positive *x* axis values indicate greater weighting of high relative to low performance; positive *y* axis values indicate greater weighting of structurally disadvantaged relative to advantaged players. Dashed lines at zero denote null sensitivity. Shaded regions delineate relative weighting of performance versus structural productivity, defining distinct merit regimes (red = corrective; yellow = legitimizing; green = cue-invariant; purple = performance-dominant; blue = performance-lenient). Point color reflects individual-level sensitivity attenuation (auxiliary parameter: gamma, γ). (**D**) Merit regimes. Mean allocation to each receiver category (out of $1), grouped by cluster-derived latent sensitivity. Error bars indicate ±1 SEM.

To assess whether behavioral patterns reflected differences in how productivity and performance were weighted, we analyzed decisions using the same participant-level continuous choice model as in study 2, with the key difference that, in this study 3, productivity and performance were encoded as trial-level binary predictors describing the observed tournament player relative to the participant (see Materials and Methods). As in study 2, group-level information-criterion values AIC and BIC favored the integrated model [table S8 (Model comparison). Also: table S9 (Model comparison winning table), and figs. S6 (Parameter recovery), S7 (Simulations), S8 (Confusion matrix), and S9 (Held-out predictive performance)].

Following the approach in study 2, heterogeneity in how participants weighted tournament metrics was characterized by classifying individuals into decision regimes based on their fitted raw parameters (Fig. 4C). The largest proportion of participants were classified as largely insensitive to tournament information (null/cue-invariant: 42.4%, *N* = 53). Of these, 18 consistently kept the full endowment, six always allocated $0.50, one always gave away $1, and one always allocated $0.20, with the remaining showing weak differentiation in allocations across receiver groups. Among participants exhibiting stronger sensitivity on at least one dimension, the most prevalent regime was corrective (31.2%, *N* = 39). A smaller subset of participants showed a legitimizing profile (14.4%, *N* = 18), and a minority were classified as performance-dominant (12%, *N* = 15) (Fig. 4D).

#### Study 4: Structural productivity and performance are weighted during self-interested, nonimpartial allocations

Given that judgements of deservingness are often made by individuals with a personal experience and stake in the systems they evaluate, we extended our design to a context involving direct self-interest and prior exposure to relevant structural conditions. Thus, in study 4, participants (*N* = 388) who had themselves played the tournament undertook an allocation task through a modified DG. These participants were informed that they would be matched with other players (“receivers”) and had the option to share a $1 endowment. Before deciding, participants were shown both their own and the receiver’s tournament metrics.

On average, participants allocated $0.27. Advantaged participants allocated slightly more to receivers (*M* = $0.31) compared to disadvantaged participants (*M* = $0.24). Disadvantaged players gave, on average, $0.31 to other disadvantaged receivers and $0.18 to advantaged receivers, whereas advantaged players gave, on average, $0.37 to disadvantaged receivers and $0.24 to other advantaged receivers. Advantaged low performers gave the most, allocating an average of $0.37, followed by disadvantaged low performers who gave $0.30, then advantaged high performers gave $0.24, with disadvantaged high performers giving the least, an average of $0.18 [Fig. 5A; see also fig. S10 (In- versus out-group allocations)].

**Fig. 5.**
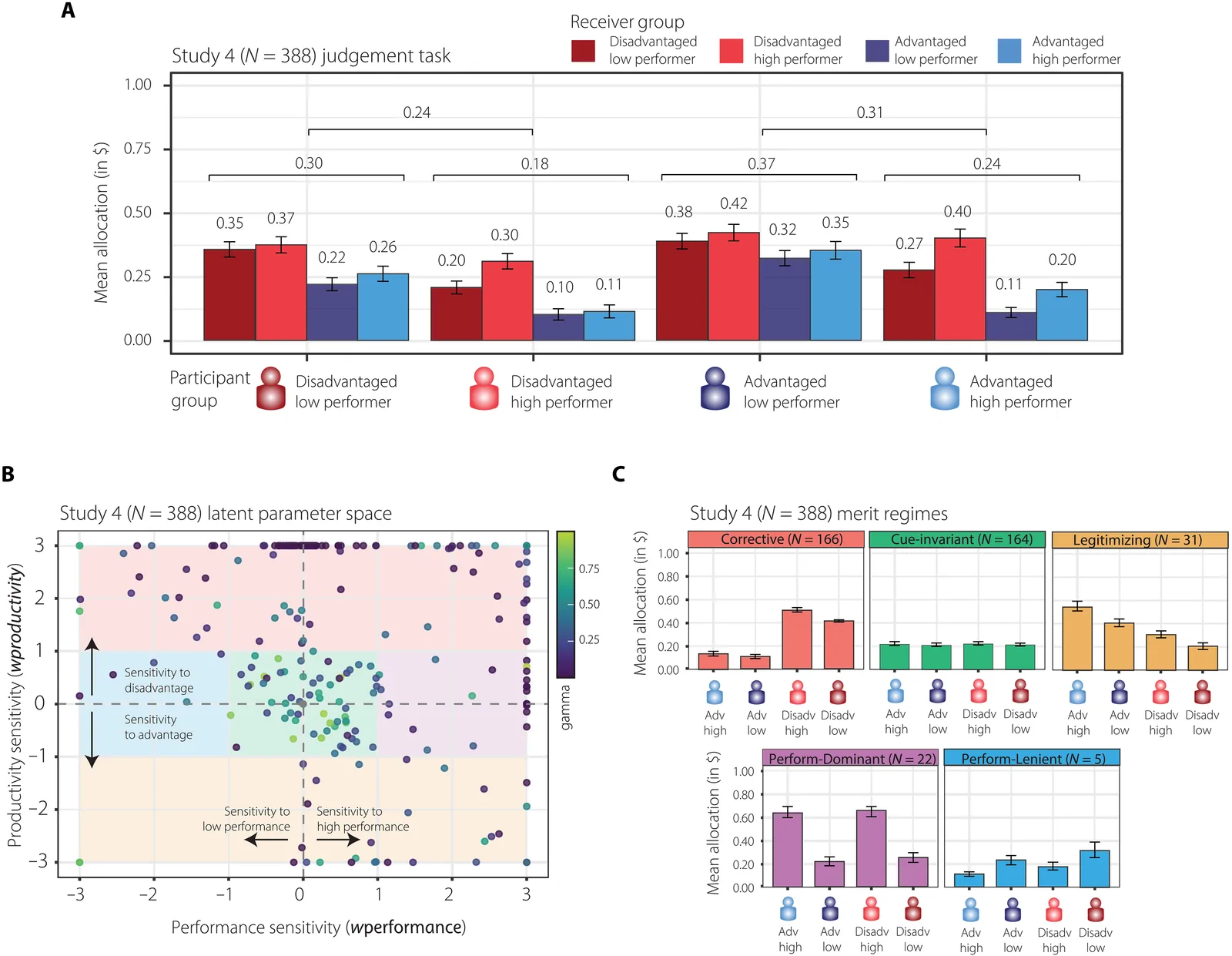
Allocations in study 4. (**A**) Study 4 judgement task (pairing with players). Mean allocation by participant tournament group as a function of receiver tournament group. Error bars indicate ±1 SEM. (**B**) Latent parameter space. Individual raw parameter estimates plotted as sensitivity to performance (*w*_performance_; *x* axis) and sensitivity to structural productivity (*w*_productivity_; *y* axis). Dashed lines at zero denote null sensitivity. Shaded regions delineate relative weighting of performance versus structural productivity, defining distinct merit regimes (red = corrective; green = cue-invariant; yellow = legitimizing; purple = performance-dominant; blue = performance-lenient). Point color reflects individual-level sensitivity attenuation (auxiliary parameter: gamma, γ). (**C**) Merit regimes. Mean allocation to each receiver category (out of $1), grouped by cluster-derived latent sensitivity. Error bars indicate ±1 SEM.

We conducted a linear mixed-effects model to examine the effects of participants’ productivity, participants’ performance, receiver’s productivity, receiver’s performance, the interaction between participant productivity and performance and the interaction between receiver productivity and performance on allocations. The results revealed a nonsignificant effect of participant productivity (H1: β = −0.062, SE = 0.035, *P* = 0.072). Conversely, a main effect of participant performance was found with low performers allocating significantly more than high performers (H2: β = 0.123, SE = 0.035, *P* < 0.001). A main effect of receiver productivity was also found with participants allocating more to Disadvantaged receivers (H3: β = 0.144, SE = 0.005, *P* < 0.001), and an effect of receiver performance with participants allocating significantly less to those who were lower performers (H4: β = −0.044, SE = 0.005, *P* < 0.001). Interaction effects between participant productivity and performance were nonsignificant (H5: *P* = 0.915); however, the interaction between receiver productivity and receiver performance was significant (H6: β = −0.025, SE = 0.008, *P* = 0.001), indicating the performance of disadvantaged receivers influenced how much they were allocated with more given to high performers (see tables S10 and S11 for pairwise comparisons).

Next, to further assess whether the integration of productivity and performance generalized to this context in which participants had experienced the tournament, we applied the same participant-level continuous choice model as in studies 2 and 3 to allocation decisions in study 4. As before, we compared the integrated latent value model against reduced models (see Materials and Methods), with group-level information-criterion values AIC and BIC showing strong favoring of the integrated model [see tables S12 and S13 (Model comparison), as well as figs. S11 (Parameter recovery), S12 (Simulations), S13 (Confusion matrix), and S14 (Held-out predictive performance)]. Overall, these results indicate that, even when participants have themselves experienced either the advantaged or disadvantaged tournament conditions, on average, players integrate productivity and performance cues when allocating resources (Fig. 5B).

Following the approach adopted in studies 2 and 3, heterogeneity in how participants weighted tournament information was characterized by classifying individuals into regimes based on their sensitivity parameters. A large proportion of participants were functionally insensitive to tournament information (null/cue-invariant: 42.3%, *N* = 164). Of these, 83 always kept the full endowment, 12 gave away $0.50, five gave away $1, and one always allocated $0.10, with the remaining showing weak differentiation to the receiver groups. Among participants exhibiting sensitivity on at least one dimension, the most prevalent regime was corrective (42.8%, *N* = 166). A smaller subset of participants showed a legitimizing profile (7.99%, *N* = 31). A subset of participants was classified as performance-dominant (5.67%, *N* = 22), and a minority were performance-lenient (1.29%, *N* = 5) (Fig. 5C).

### Study 4: Experience of (dis)advantage is associated with merit regime classification

Having established in studies 3 and 4 that structural productivity and performance shape allocations and individuals can be classified into corresponding regimes across our studies, we next aimed to explore what may impact the expression of these regimes. That is, we specifically sought to better understand what might predict an individual’s preference weighting and consequent strategy. Study 4 provided an opportunity to test this as participants first took part in the tournament before making decisions, allowing us to test whether experiencing a structurally advantaged or disadvantaged position alters the occurrence of strategies.

Across studies, participants could be grouped into similar decision profiles, including null, corrective, legitimizing, and performance-based strategies [see figs. S15 to S17 (regime disaggregation)]. However, the relative prevalence of regimes differed across contexts (Fig. 6A). Within each, behavioral profiles remained qualitatively consistent (as particularly evident in studies 3 and 4), indicating that the task context itself did not substantially alter the internal logic of decision-making strategies.

**Fig. 6.**
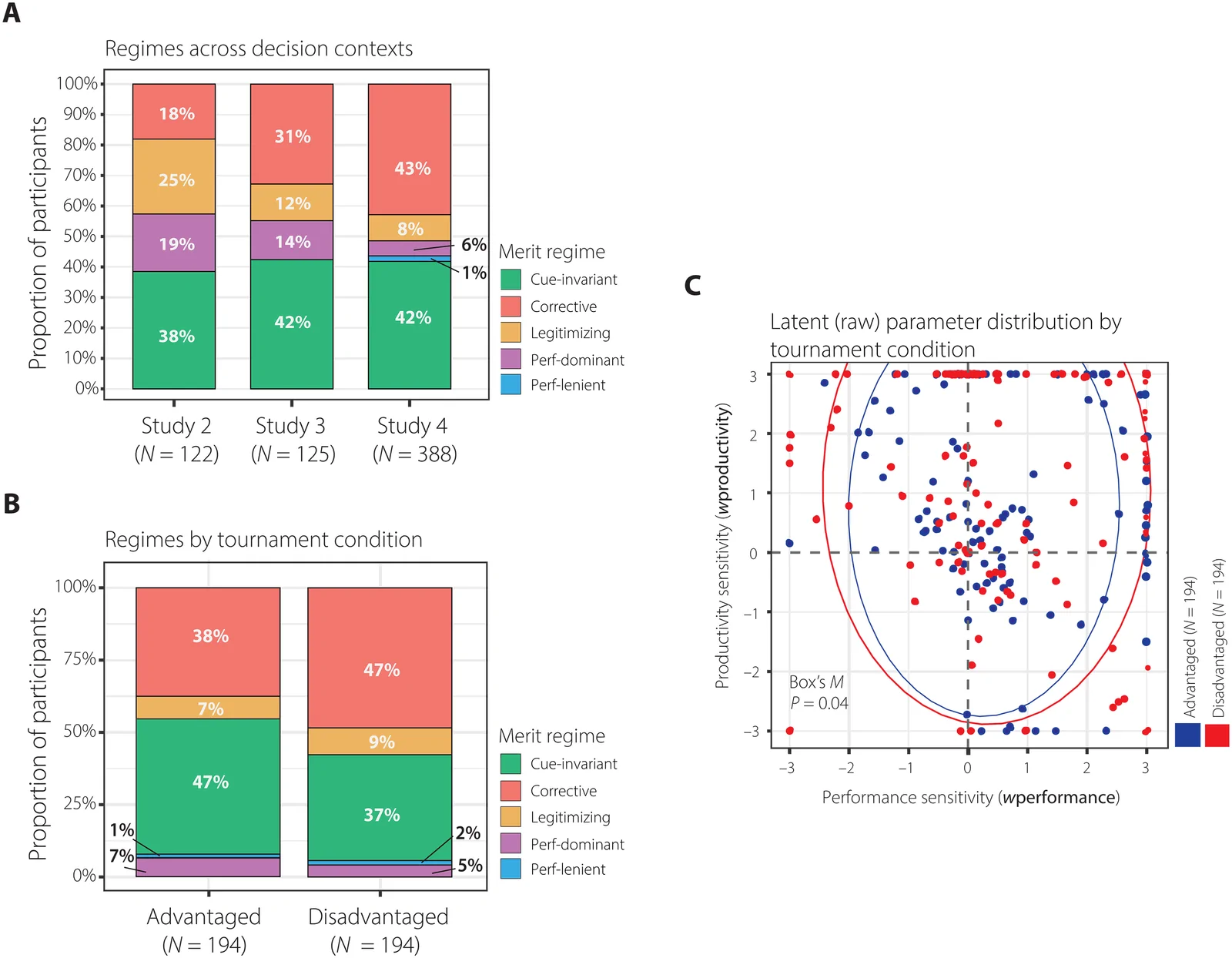
Exploratory analyses of merit regimes across contexts and conditions. (**A**) Regimes across decision contexts. Stacked bar graphs reflecting proportion of merit regimes (allocation strategies) for studies 2, 3, and 4, respectively (red = corrective; green = cue-invariant; yellow = legitimizing; purple = performance-dominant; blue = performance-lenient). (**B**) Regimes by tournament condition. Stacked bar graphs reflecting proportion of study 4 merit regimes, by tournament condition (red = corrective; green = cue-invariant; yellow = legitimizing; purple = performance-dominant; blue = performance-lenient). (**C**) Latent parameter distribution by tournament condition. Study 4 parameter space with each point representing one participant’s jointly estimated raw performance and structural productivity sensitivity. Ellipses depict 95% data ellipse of the joint (raw) parameter distributions within each structural productivity group (blue = structurally advantaged; red = structurally disadvantaged). Differences in variance-covariance structure were assessed using Box’s *M* test (*P* = 0.04).

Within study 4, although both groups exhibited overall similar allocation patterns, prioritizing disadvantaged over advantaged receivers, disadvantaged participants were more often classified in the corrective strategy (47.4%) than advantaged participants (38.1%), and within the advantaged participants, the null regime was the most prevalent strategy [47.4%; Fig. 6B, fig. S18 (Cluster allocations)]. Consistent with this, a multinomial logistic regression predicting decision-regime membership from participants’ own tournament productivity and performance (reference = corrective), revealed that experiencing structural disadvantage reduced the odds of expressing the null relative to the corrective regime (OR = 0.60, *z* = −2.25, *P* = 0.025).

To further explore experience-dependent differences, we compared the joint distribution of sensitivities by tournament condition. A permutation test for homogeneity of multivariate dispersions revealed greater dispersion among disadvantaged participants (*F* = 5.96, *P* = 0.01; centroid distance: disadvantaged = 2.01, advantaged = 1.69). Similarly, Box’s *M* test showed that the variance-covariance structure of the raw parameters differed statistically between groups [χ^2^(3) = 8.15, *P* = 0.043; Fig. 6C]. Together, these results suggest that experiencing structural (dis)advantage does not appear to generate distinct decision processes, but rather may redistribute the prevalence of participants within a shared parameter space.

Further exploratory analyses examining associations between sociodemographic characteristics (such as political orientation) and model-derived parameters are reported in tables S14 to S22.

## DISCUSSION

Merit is foundational to notions of justice, widely endorsed as a principled means by which resources ought to be distributed (*2*, *12*, *14*, *15*). As such, merit plays a crucial role in guiding consequential allocations across social, economic, and political domains (*2*). Within this framework, inequality arising from luck is typically regarded as unfair, whereas inequality attributed to differences in merit is viewed as deserved, allowing merit to serve as a moral justification for unequal outcomes (*7*, *12*, *15*, *19*). Despite its normative prominence, up until now, merit has been treated as a self-evident property, most often equated with a person’s performance or productivity, and contrasted with luck (*4*, *7*, *16*–*18*, *24*). Prior work on fairness and social preferences has therefore largely treated merit as a fixed input into distributive decisions (*4*, *8*, *13*), rather than a construct that itself requires investigation. Critically, whilst existing models formalize how individuals respond to inequality (*20*, *25*, *26*), they leave unaddressed the prior, formative question of how deservingness is determined.

Here, across four preregistered, incentivized studies with samples of US-based participants (total *N* = 759), we challenge the aforementioned assumptions by offering a formal account, based on experimental and computational evidence, of how merit is constructed and applied in systems where structural conditions obscure how performance and productivity are generated. Through resource allocations reflecting judgements of deservingness captured across contexts, we demonstrate that individuals construct merit by weighing how well someone performed given how much opportunity they had to do so. Merit is thus revealed not as a fixed, universal rule, but individual judgement produced by a quantifiable process that shapes distributive decision-making.

A central contribution of this work is to experimentally and computationally define merit as a composite construct. By devising a real-effort tournament in which productivity differences were driven by unequal opportunity, rather than differential effort alone, we aimed to simulate real-world meritocracy and dissociate two dimensions of merit that are often conflated, namely, performance (one’s effort expended) and structural productivity (one’s rewarded output as a function of what is possible given the circumstances). Structural productivity specifically captures here the combined effect of unequal productivity conditions and their material consequences. Crucially, we explicitly sought to minimize the confounding role of talent by investigating instances in which outcomes cannot plausibly be justified by differences in ability, but must instead be inferred only from outcomes produced under unequal conditions. Players in the tournament were sensitive to this manipulation both behaviorally and subjectively. Structurally disadvantaged players exhibited lower overall absolute productivity, as well as perceived greater unfairness, reduced control, increased belief in the role of luck in determining outcomes, and a heightened effort-reward imbalance, compared to those advantaged. The two merit dimensions directly determined how deservingness was actually assessed, including across impartial decisions as well as choices involving self-interest. This establishes that structural productivity, that is, the pathway through which output is achieved, is not merely contextual background information, but is used as an integral input into evaluations of merit and consequent allocations of resources.

Crucially, in contrast with how merit has traditionally been conceptualized or documented, including across psychology (*2*, *12*, *14*, *27*), behavioral economics (*4*, *15*, *16*, *24*, *28*), law and philosophy (*3*, *23*, *29*), merit judgements did not collapse into a single dimension, such as performance or productivity maximization, nor were they explained through a simple equality heuristic alone. Instead, decision-making across contexts was explained by a latent value-difference model in which structural productivity and performance were jointly integrated into an internal value signal guiding allocations. This formulation allows merit to be specified as an explicit function of observable inputs, rather than inferred post-hoc from behavior. By defining merit in this way, we thus move beyond descriptive accounts of meritocratic preferences as an exogenous moral principle and instead provide a model-based account of merit as an endogenous variable constructed through an evaluative process.

At the same time, our approach allows for, and indeed demonstrates, that the process of merit computation is not uniform across individuals. While most participants integrated both structural productivity and performance, they differed systematically in how these dimensions were weighted. This heterogeneity gave rise to distinct merit regimes, namely, allocation strategies that could be classified as corrective (predominantly favoring structural disadvantage), legitimizing (primarily favoring structural advantage), or performance-focused. We also identify large proportions of cue-invariant subgroups whose allocations can be characterized by the absence of adjustment to the dimensions of merit presented. This pattern is not interpreted simply as evidence of inattention or random responding alone, as allocation profiles of cue-invariant participants are consistent with different underlying motives, including principled egalitarianism (i.e., 50/50 split), purely self-interested responding, and strong other-regarding preferences. Together, the regimes reflect regions of parameter space with behavioral patterns that align with diverging ideals of fairness (*4*, *30*). The existence of these regimes implies that disagreements about merit need not reflect disagreement about facts, but may rather be differences in how meritocratic cues are normatively incorporated into individual valuation processes. From this perspective, therefore, debates about deservingness are not merely ideological but potentially computational, grounded in dissimilar weightings applied to the same inputs.

An important proposition within the present work is that personal experience is associated with a shift in the prevalence of merit regimes. Players who experienced structural disadvantage were more likely to be classified as corrective, whereas players who had experienced advantage more often exhibited cue-invariant preferences. While the two groups adopted a similar behavioral tendency of prioritizing allocations toward those who had experienced structural disadvantage, this was not generated through the same composition of latent strategies. Yet, across conditions, the underlying valuation remained similar. This distinction between the consistency of the decision process and how strategies are distributed suggests that one’s own path may influence which merit regime is used, rather than reshaping how merit is computed.

This empirically grounded framework of deservingness has important implications for clarifying when and why inequality is legitimized in ostensibly meritocratic systems, as well as for better understanding what has been described as the “myth of meritocracy” (*2*) or the “tyranny of merit” (*3*). If merit were defined solely by observed outputs, correcting misperceptions about opportunity should suffice to align judgements to ensure more equitable allocations. Our findings suggest otherwise. Even when structural disadvantage is transparent, individuals still differ in how much weight they assign to it when computing merit. Consequently, consensus about fairness does not necessarily follow from consensus about facts. That is, even if people agree on who had more opportunity, who performed better, and how unequal a system was, they may still disagree about what outcome is fair, due to differences in how merit dimensions are weighted. Using “merit” to allocate resources thus necessitates precision about how the concept is defined. Moreover, efforts to design equitable systems benefit from an understanding of individual differences, as what counts as deserved depends on the distribution of merit-computation profiles within a population, and how the social context of the decision-maker might activate their expression.

Methodologically, this work demonstrates the value of treating moral and social judgements as computational processes rather than static preferences (*31*–*36*). By specifying merit as a value function, carefully designing an experiment to allow examination of its component parts, and estimating individual-level parameters, we were able to quantify shared structure and meaningful heterogeneity in decision-making. This approach complements existing work on social preferences (*8*, *31*, *33*, *37*, *38*), by specifying the internal trade-offs that generate social behavior. Through the current findings, we extend this approach to recognize merit itself as a fundamental psychological process.

Several limitations should, nonetheless, be noted. First, the current work focused on allocations in a controlled experimental setup. This design allowed us to test the behavioral and computational structure of merit judgements under transparent and incentive-compatible conditions, though importantly represents a minimal and simplified form of structural inequality. Future work is needed to examine the generalizability of computations identified here to naturalistic contexts which contain multidimensional, and often imperfectly observable, signals of merit (such as talent, skill, credentials, etc.). Second, across studies, both the tournament outcome and the allocation decision were expressed through the same format, namely money. In real-world settings, structural advantage may of course result in outcomes such as grades, status, access, or career opportunities. Allocative responses might also take different forms, including financial (re)distribution, policy preferences, or punishment. Further research could usefully extend these findings to examine whether similar deservingness judgements and allocation strategies emerge when the source of inequality and available response differ in format. Third, participants were U.S.-based and English-speaking. The sampling approach allowed us to test the paradigm across comparable samples; however, meritocratic beliefs and norms may vary substantially across societies (*4*). Therefore, it would be crucial to examine whether the patterns identified here generalize across broader cultural, institutional, and ideological contexts. Fourth, our multistudy design shows that merit construction emerges across different decisions, including impartial and self-interested contexts. As the studies did not manipulate contextual features in a fully crossed design, they do not allow for isolating the separate causal contribution of each. Future work could thus test whether any differences between studies are driven by the structure of the allocation, the presence of self-interest, personal tournament experience, visibility of one’s own metrics, or some combination of these factors. Fifth, while our exploratory analyses suggest that political orientation is associated with sensitivity to structural productivity (with more conservative participants in the self-interested allocation contexts associated with lower sensitivity to structural disadvantage), more research is needed to further specify the relationship between sociodemographic factors, such as political leaning, and merit weighting patterns. Last, regime classification was based on thresholds applied to latent cue-weight parameters, and thus alternative modeling approaches and partitioning of parameter space may yield complementary perspectives to advance understanding of underlying allocation strategies. Addressing these open questions would be imperative for characterizing meritocratic judgements in complex social systems, as well as for informing interventions aimed at developing merit-based systems perceived as “fair” across individuals.

Collectively, this work reframes merit not as a fixed moral axiom but as a constructed, quantifiable, decision process that arises from the evaluation of performance and structural productivity cues. By experimentally dissociating these dimensions and formalizing their integration, we provide a precise account of how (different) people could compute merit and thus decide on deservingness. This conceptualization helps explain the contested nature of meritocracy, while offering a framework for understanding how judgements of merit can sustain or challenge inequality.

## MATERIALS AND METHODS

### Ethics statements

Before data collection, ethical approval for all studies was provided by the Ethics Committee Faculty of Social Sciences, Radboud University (ECSW-2024-084). Before starting each of the experiments, all participants were informed of the general nature of the study and were asked to provide their express written consent. All measures used in the studies are described in the “Experimental procedure” section and in the Supplementary Materials. The studies, including hypotheses and analyses for behavioral results, were preregistered on OSF, which can be found at: study 1 (osf.io/agvhx), study 2 (osf.io/3rxb7), study 3 (osf.io/ma5fz), and study 4 (osf.io/krb5n).

### Participants

All participants were recruited via the Prolific platform. To enroll, participants needed to self-report being fluent in English (to understand the task instructions), residing in the United States of America (U.S.), and be over 18 years of age. Participants who had enrolled in any of our studies (studies 1, 2, 3, or 4) were excluded from participating in any of the other studies, to ensure no crossover effects from participating in more than one role (studies 1 and 2: judge; study 3: observer; and study 4: player). Experimental procedures were programmed in Gorilla Experiment Builder (www.gorilla.sc).

For study 1, 125 participants were recruited. One participant was excluded from the analysis due to a technical error which occurred during their data collection. Therefore, data from 124 participants (*M*_age_ = 39, range: 18 to 78) were analyzed. This is in line with our preregistration, where an a priori power analysis was conducted using G*Power 3.1 (*39*), which determined that a minimum sample size of 119 participants would be required to detect a small effect size (*f* = 0.10) with a significance level of α = 0.05 and a desired statistical power of 0.95. The gender distribution was broadly balanced with 69 females (56%), 53 males (43%), and 2 “other” (2%). For full details of sample sociodemographic characteristics, see table S1. Participants in this study received a flat rate fee of 6.01 GBP/hour for their participation and took approximately 29 min to complete the study. In addition, for each participant the outcome of one judgement task trial was randomly selected for payout. Participants could additionally earn bonuses for accurately estimating responses in regarding to the counting tournament. On average, participants received a bonus of 0.17 GBP, with a mean payout to tournament players being 0.50 GBP.

For study 2, 125 participants were recruited. Two participants were excluded from the analysis due to failing all three attention check questions. Therefore, data from 122 participants (*M*_age_ = 37.37, range: 18 to 71) were analyzed. As above, in line with our preregistration, an a priori power analysis determined that a minimum sample size of 119 participants would be required to detect a small effect size (*f* = 0.10) with a significance level of α = 0.05 and a desired statistical power of 0.95. The gender distribution was again broadly balanced with 62 females (51%) and 60 males (49%). For full details of sample sociodemographic characteristics, see table S2. Participants received a flat rate fee of 6.81 GBP/hour for their participation and took approximately 39 min to complete the study. In addition, for each participant the outcome of one judgement task trial was randomly selected for payout. Participants could additionally earn bonuses for accurately estimating responses regarding the counting tournament. On average, participants received a bonus of 0.17 GBP, with a mean payout to tournament players being 0.50 GBP.

For study 3, the sample consisted of 125 participants (*M*_age_ = 40.34, range: 21 to 80). This is in line with our preregistered a priori power analysis which determined that a minimum sample size of 106 participants would be required to detect a small effect size (*f* = 0.10) with a significance level of α = 0.05 and a desired statistical power of 0.95. The gender distribution was balanced with 64 males (51%) and 61 females (49%). For full details of sample sociodemographic characteristics, see table S3. Participants received a flat rate fee of 6.01 GBP/hour for their participation and took approximately 22 min to complete the study. In addition, each participant could earn extra money from (i) the outcome of one randomly selected DG trial and (ii) for accurately estimating responses in regarding to the counting tournament. On average, participants received a bonus of 1.23 GBP, with a mean payout for player receiver of 0.26 GBP.

For study 4, in line with our preregistration, we collected data to ensure that each between-subjects condition had a minimum of *N* = 90. As data collection resulted in oversampling in two conditions, participants in the oversampled groups were randomly sampled down so that no group exceeded approximately 15% in size relative to the others. Thus, data from 388 participants were analyzed (structurally advantaged: *N* = 194; structurally disadvantaged: *N* = 194). This is in line with our preregistered a priori power analysis which determined that a minimum sample size of 360 participants would be required to detect a small effect size (Cohen’s *f*^2^ = 0.05) with a significance level of α = 0.05 and a desired statistical power of 0.90. The sample mean age was 40 years (range: 18 to 83), and the gender distribution was balanced with 198 males (51%), 184 females (47%), 5 other (1%), and 1 preferred not to say. For full details of sample socio-demographic characteristics, see table S4. Participants received a flat rate fee of 6.31 GBP/hour for their participation and took approximately 33 min to complete the study. In addition, participants could earn extra bonus money from (i) the tournament, (ii) the outcome of one randomly selected DG trial, and (iii) for accurately answering questions about the tournament. On average, participants received a bonus of 1.96 GBP.

### Experimental procedure

#### Stimuli, task, and design

The experiment consisted of two phases, a tournament followed by an allocation stage (“the judgement task”). Participants in all studies completed the allocation phase, which was adapted across studies to capture differences in neutrality and self-interest inherent in the choice process (described more fully below). Only participants from study 4 took part themselves in the tournament, participating as players.

#### Tournament

Participants either observed the outcomes of (studies 1, 2, and 3) or themselves competed in (study 4) a real-effort task. The tournament was designed to be easy to understand, not reliant on any particular cognitive abilities or specific skills, and difficult to cheat on, thus ruling out any impact of inherent talent on outcomes. Participants were informed that the task required counting zeros in tables comprised of ones and zeros (*40*). The tournament consisted of three rounds (each lasting 3 min) with two breaks, resulting in a total of 9 min of tournament time. For each table presented, players had to count and then enter the correct number of zeros within two attempts, each lasting 4 s (totaling 8 s). Failure to provide the correct answer after both attempts resulted in a 30-s penalty lockout from that round.

Before the tournament began, players were randomly assigned to one of two conditions: a structurally advantaged condition (labeled as “easy”) in which they would be presented with simple tables featuring a single row of five numbers of ones or zeros, or the structurally disadvantaged condition (labeled as “difficult”) in which they would be presented with more complex tables, each comprising a grid of five rows of five numbers of ones and zeros. During the instruction phase, players practiced one round with both table types to help them understand the nature of the task and the potential tables that could be assigned.

Players were informed they were competing to earn a monetary reward based on their total number of correct tables answered, as compared to other players who had previously played the tournament. The exact reward amounts were not specified. However, players were aware that the top overall performers would receive a “high” reward approximating $3.00, while the bottom performers would receive a “low” reward of approximately $0.19. This ensured that players were aware of a notable (approximately 16-fold) disparity in payouts between top and bottom half of players. Before beginning, players were required to pass five comprehension questions to ensure they understood the rules of the game. Thereafter, the players were informed of the table type assigned to them and proceeded with the three rounds.

After all rounds were complete, but before being informed of the tournament outcome, players were asked to indicate how much they felt they deserved for their performance on a scale from $0 to $3.00 (in increments of 0.10), using a slider with a randomized starting point. Players were then shown their tournament performance. Specifically, each player’s actual performance was converted into a matrix of three “tournament metrics.” These metrics captured: table difficulty (whether the player was assigned easy or difficult tables), group performance (whether the player ranked as a low or high performer within their table group), and overall performance (whether the player received a low or high reward). On the basis of their actual performance, players were assigned to one of four possible combinations of these metrics (Table 1). Thresholds used to classify low versus high performance within each table condition were determined from the empirical distribution observed in pilot data (difficult condition: 13 tables; easy condition: 174 tables). Overall performance (reward) was determined using a median split of the sample. Reward classification therefore depended entirely on participants’ actual performance relative to these thresholds, and data collection continued until each performance category reached the predefined minimum sample size.

**Table 1.** Tournament metrics reflecting participants’ real performance.

Participant categories*	Table difficulty	Group performance†	Overall performance (reward)
Advantaged high performer	Easy	High	High
Advantaged low performer	Easy	Low	High
Disadvantaged high performer	Difficult	High	Low
Disadvantaged low performer	Difficult	Low	Low

* Participant categories are used only for purposes of reporting these results and were never shown to any participant. The terms “disadvantaged,” “advantaged,” “structural,” “injustice,” “inequality,” or “status” were never used or presented to participants at any point.

† In the difficult condition, low performers completed 0 to 13 tables and high performers completed 14 to 60 tables. In the easy condition, low performers completed 62 to 173 tables and high performers completed 175 to 283 tables.

As overall reward directly followed from the structural conditions under which performance took place, table difficulty and reward level are treated, in our analyses, as a single construct, which we refer to as “structural productivity.” This aligns with our aim of capturing situations in which structural advantage produces higher observable outcomes, but which precludes us from fully separating the effects of opportunity independent of such material reward. An “easy tables/high reward” combination reflects a structurally advantaged context, whereas a “difficult tables/low reward” outcome reflects a structurally disadvantaged context. Group performance, by contrast, indexes individual effort relative to that of peers within the same structural context and is thus separable from structural productivity. The player combinations of “difficult tables/low reward” and “easy table/high reward”, are referred to hereafter as “(structurally) disadvantaged” and “(structurally) advantaged,” respectively. None of these terms (“disadvantaged,” “advantaged,” “structural,” “injustice,” or “status”) were ever presented to participants. Thus, while participants in all studies were shown each player’s raw tournament metrics [i.e., tables completed, group performance, and overall performance (reward)], participant categories (namely, advantaged high performer, advantaged low performer, disadvantaged high performer, and disadvantaged low performer) are used only for purposes of reporting our results.

#### Allocations (the judgement task)

The judgement task gave participants the opportunity to allocate an additional amount to tournament players. All participants across all studies completed a version of the task, though each study differed in terms of the role of the participant and whether one or two players were evaluated per trial. During the task, participants were presented with the three converted tournament metrics (i.e., tables completed, group performance, and overall performance) of the respective player(s) and used a slider (with a randomized starting point) to indicate the amount they wished to allocate to the player(s).

Before making any decisions, participants in studies 1, 2, and 3 completed a brief 3-min practice version of the task to familiarize themselves with the performance information they would later judge or evaluate. Participants were then asked to estimate how many tables each group (easy versus difficult) had completed. These estimates were incentivized and served as a measure of perceived group performance. Before beginning the allocation trials, participants in all studies were required to pass five comprehension questions to ensure understanding of the tournament metrics and task rules.

In studies 1 and 2, participants were informed that they would act as judges of the tournament performance and therefore had no personal stake in the outcomes. Here, two tournament players (and their metrics) were presented simultaneously, and participants were given the opportunity to allocate between $0 and $1 ($0.10 increments) to player 2, with the remaining amount automatically allocated to player 1. Study 1 consisted of 40 trials (10 unique pairings × four repeated measures). Study 2 followed the exact same procedure, but participants were not provided with information about the approximate $0.19 to $3.00 payout difference from the tournament. Instead, they were only told that rewards differed substantially between high and low rewards, without providing any exact or approximate amounts, and an increased number of 80 trials (16 pairings x five repeated measures). In both studies, one randomly selected allocation was paid out to the actual tournament players.

In study 3, participants had also not participated in the tournament but were assigned the role of observer. Specifically, participants were told that observers do not participate in the tournament themselves, but had the chance to earn rewards by answering questions about the tournament and the people who played the tournament. They completed a modified DG in which a single tournament player (and their metrics) was presented on each trial. Participants could share a $1 endowment between themselves and that player, across 24 trials [namely, four receiver categories (advantaged high performer, advantaged low performer, disadvantaged high performer, and disadvantaged low performer) × six repetitions per receiver category]. Participants were thus impartial to the tournament but self-interested, as one randomly selected allocation affected their own bonus payment and that of a player. Before ending the task, participants were also paired with a receiver who had also observed the counting tournament. During this phase, participants received no other information about the “observer” receiver but were similarly given $1 to share if they wished. This phase was designed to pair participants with a member who had the same role as themselves to establish a DG baseline.

In study 4, participants had themselves competed in the tournament, and subsequently completed a modified DG. On each of 24 trials (four receiver categories × six repetitions per category), participants viewed both their own tournament metrics and those of another player (i.e., the receiver), and could make $1 allocation decisions as self-interested and partial actors of the tournament. At the end of the study, participants were informed that they had also been placed in the role of a receiver for another participant, and would receive additional bonus money based on a randomly selected DG trial from another tournament player.

These studies were collectively designed as a sequence across increasingly complex decision contexts, rather than as a fully crossed design isolating the contribution of each new feature within each allocation task.

#### Post-task measures

After completing the allocations, participants from all studies answered additional questionnaires, including whether they believed the tournament depended on luck or effort, and their perceptions of tournament fairness (for a full list of questionnaires, see the Supplementary Materials). Last, participants were asked to reflect on their allocation strategy through an open-ended text prompt.

### Computational models of merit

Across studies 2, 3, and 4, behavior was analyzed using the same participant-level continuous choice model, which formalizes how information about structural productivity and performance is transformed into allocation decisions. The core model specification, estimation procedure, and validation analyses were identical across studies. Differences between studies arose solely from the trial structure and the manner in which the merit cues were encoded at trial level.

#### Model specification

Allocation behavior was assumed to be guided by a latent subjective value signal derived from structural productivity and performance information. For a given participant and trial t, the subjective value difference was defined as:

##### Integrated/full model

$$\Delta V_{t}=w_{\text{productivity}}.s_{\text{productivity},t}+w_{\text{performance}}.s_{\text{performance},t}$$ (3)

where *s*_productivity,*t*_ and *s*_performance,*t*_ denote trial-level predictors encoding structural productivity and performance respectively, and *w*_productivity_ and *w*_performance_ capture individual sensitivity to each dimension. Positive values of *w*_productivity_ indicate preferences favoring structurally disadvantaged players, whereas positive values of *w*_performance_ indicate preferential allocation to higher-performing players. To allow for individual differences in how strongly value differences governed choice behavior, the value signal was attenuated by an auxiliary parameter, γ ∈ (0,1), yielding a scaled value

$$\eta_{t}=\left(1-\gamma \right)\cdot \Delta V_{t}$$ (4)

The sensitivity-attenuated value signal was mapped to an expected allocation using a logistic function

$$\mu_{t}=\frac{1}{1+\text{exp}\left(-\eta t\right)}$$ (5)

which ensured predicted allocations were bounded in the unit interval. Observed allocations were modelled using a beta distribution parameterized by this predicted mean and a fixed precision parameter (κ = 30), allowing for continuous bounded responses with trial-level variability. In addition to the full model incorporating both productivity and performance, three reduced models were considered.

##### Productivity-only model

$$\Delta V_{t}=w_{\text{productivity}}\cdot s_{\text{productivity},t}\;\text{with}\;w_{\text{performance}}=0$$ (6)

##### Performance-only model

$$\Delta V_{t}=w_{\text{performance}}\cdot s_{\text{performance},t}\;\text{with}\;w_{\text{productivity}}=0$$ (7)

##### Null/Cue-invariant model

$$\mu_{t}=0.5$$ (8)

Participants whose responses exhibited effectively zero variance were classified as “constant responders” and evaluated under the null model.

#### Study-specific implementation – Study 2: Third-party judges

In study 2, given that participants here divided a fixed endowment between two tournament players who differed in structural productivity (advantaged versus disadvantaged) and performance (high versus low performer), productivity and performance were contrast-coded as relative differences between players, yielding trial-level predictors *s*_productivity,*t*_ and *s*_performance,*t*_ taking values of −1, 0, or +1. Positive parameter estimates therefore indicated a tendency to favor player 2 when that player was disadvantaged or high performing.

All models were fit independently for each participant using maximum likelihood estimation with extensive multistart optimization. Model comparison was performed at the individual level using AIC, BIC, and likelihood-ratio tests. Robustness and identifiability were assessed using parameter recovery simulations, model recovery analyses, and held-out predictive checks. Full details of model fitting, comparison, parameter recovery, model recovery, and held-out predictive analyses are provided in Supplementary Methods (Modeling procedure).

#### Study-specific implementation – Studies 3 and 4: Observer and tournament players

Studies 3 and 4 used the same computational framework as study 2, but differed in trial structure. On each trial, participants allocated a fixed endowment between themselves and a single other tournament player. In study 3, participants acted as a neutral observer and in study 4 participants had themselves experienced the tournament as players. In these studies, structural productivity and performance were encoded as binary predictors describing the observed player relative to the participant (*s*_productivity,*t*_, *s*_performance,*t*_ ∈{−1,+1}). Positive parameter estimates thus reflected greater allocations to disadvantaged and high-performing players, respectively. Aside from this difference in encoding, model specification, parameter estimation, reduced-model comparisons, and validation procedures were identical to those used in study 2. Full details of model fitting, comparison, parameter recovery, model recovery, and held-out predictive analyses are provided in Supplementary Methods (Modeling procedure).

### Statistical analyses

All statistical analyses were performed in RStudio (2021.09.0) (*41*). Data processing and transformation were performed using the dplyr, tidyr, and purrr packages. Regression analyses were estimated using mixed-effects models implemented in the lme4 package, including random intercepts for participants to account for repeated observations. Clustering and model-based classification analyses were performed using the cluster, factoextra, and mclust packages, and robust estimation procedures used functions from robustbase. Figures were generated using ggplot2.

## Supplementary Materials

### This PDF file includes:

Supplementary Methods Supplementary Results Tables S1 to S22 Figs. S1 to S18

## References

[1] C. Watkins-Hayes, E. Kovalsky, *The Discourse of Deservingness* (Oxford Univ. Press Oxford, 2016), vol. 1.

[2] I. R. Hadden, C. Darnon, L. Doyle, M. J. Easterbrook, S. Goudeau, A. Cimpian, Why the belief in meritocracy is so pervasive. Trends Cogn. Sci. 29, 101–104 (2025). 10.1016/j.tics.2024.12.008 39794244

[3] M. J. Sandel, *The Tyranny of Merit: What’s Become of the Common Good?* (Farrar, Straus and Giroux, 2020).

[4] I. Almås, A. W. Cappelen, E. Ø. Sørensen, B. Tungodden, Fairness across the world. Q. J. Econ. 141, qjag044 (2026).

[5] D. Fehr, M. Vollmann, Misperceiving economic success: Experimental evidence on meritocratic beliefs and inequality acceptance. J. Econ. Inequal. 23, 835–855 (2025).

[6] A. Alesina, G.-M. Angeletos, Fairness and redistribution. Am. Econ. Rev. 95, 960–980 (2005).

[7] I. Almås, A. W. Cappelen, B. Tungodden, Cutthroat capitalism versus cuddly socialism: Are Americans more meritocratic and efficiency-seeking than Scandinavians? J. Polit. Econ. 128, 1753–1788 (2020).

[8] L. M. Bas, I. D. Roberts, C. A. Hutcherson, A. Tusche, A neurocomputational account of the link between social perception and social action. eLife 12, RP92539 (2025). 10.7554/eLife.92539 PMC12002797 40237179

[9] S. Williamson, K. Soderstrom, V. McDermott, J. Connor, N. Donnelly, C. Deen, C. Ren, Reconceptualising merit in the public sector: Introducing a new typology. Aust. J. Public Adm., 1–14 (2025).

[10] E. J. Castilla, A. Ranganathan, The production of merit: How managers understand and apply merit in the workplace. Organ. Sci. 31, 909–935 (2020).

[11] R. Chetty, J. N. Friedman, E. Saez, N. Turner, D. Yagan, Income segregation and intergenerational mobility across colleges in the United States. Q. J. Econ. 135, 1567–1633 (2020).

[12] L. S. Son Hing, D. R. Bobocel, M. P. Zanna, D. M. Garcia, S. S. Gee, K. Orazietti, The merit of meritocracy. J. Pers. Soc. Psychol. 101, 433–450 (2011). 10.1037/a0024618 21787093

[13] A. W. Cappelen, J. Konow, E. Ø. Sørensen, B. Tungodden, Just luck: An experimental study of risk-taking and fairness. Am. Econ. Rev. 103, 1398–1413 (2013).

[14] S. K. McCoy, B. Major, Priming meritocracy and the psychological justification of inequality. J. Exp. Soc. Psychol. 43, 341–351 (2007).

[15] A. W. Cappelen, A. D. Hole, E. Ø. Sørensen, B. Tungodden, The pluralism of fairness ideals: An experimental approach. Am. Econ. Rev. 97, 818–827 (2007).

[16] A. W. Cappelen, K. O. Moene, S.-E. Skjelbred, B. Tungodden, The merit primacy effect. Econ. J. 133, 951–970 (2023).

[17] L. K. Gee, M. Migueis, S. Parsa, Redistributive choices and increasing income inequality: Experimental evidence for income as a signal of deservingness. Exp. Econ. 20, 894–923 (2017).

[18] L. J. Lefgren, D. P. Sims, O. B. Stoddard, Effort, luck, and voting for redistribution. J. Public Econ. 143, 89–97 (2016).

[19] P. Andre, Shallow meritocracy. Rev. Econ. Stud. 92, 772–807 (2025).

[20] G. E. Bolton, A. Ockenfels, ERC: A theory of equity, reciprocity, and competition. Am. Econ. Rev. 90, 166–193 (2000).

[21] M. McKeown, Structural injustice. Philos. Compass 16, e12757 (2021).

[22] I. M. Young, *Justice and the Politics of Difference* (Princeton Univ. Press, 1990).

[23] D. Markovits, F. Sanders, *The Meritocracy Trap* (Allen Lane, London, 2019).

[24] B. Bartling, A. W. Cappelen, I. L. Skarpeid, E. Ø. Sørensen, B. Tungodden, The talent paradox: Why is it fair to reward talent but not luck? Eur. Econ. Rev., 105268 (2026).

[25] E. Fehr, K. M. Schmidt, A theory of fairness, competition, and cooperation. Q. J. Econ. 114, 817–868 (1999).

[26] G. Charness, M. Rabin, Understanding social preferences with simple tests. Q. J. Econ. 117, 817–869 (2002).

[27] A. Rustichini, A. Vostroknutov, Merit and justice: An experimental analysis of attitude to inequality. PLOS ONE 9, e114512 (2014). 10.1371/journal.pone.0114512 PMC4260855 25490094

[28] M. Drouvelis, Z. Qiu, Luck-based versus merit-based income shifts fairness perceptions and promotes cooperation in a public goods game. Oxf. Econ. Pap. 78, 335–349 (2026).

[29] L. Pojman, Merit: Why do we value it? J. Soc. Philos. 30, 83–102 (1999).

[30] J. M. van Baar, L. J. Chang, A. G. Sanfey, The computational and neural substrates of moral strategies in social decision-making. Nat. Commun. 10, 1483 (2019). 10.1038/s41467-019-09161-6 PMC6445121 30940815

[31] S. Vahed, E. P. Galván, A. G. Sanfey, Computational modeling of social decision-making. Curr. Opin. Psychol. 60, 101884 (2024). 10.1016/j.copsyc.2024.101884 39278165

[32] A. Konovalov, J. Hu, C. C. Ruff, Neurocomputational approaches to social behavior. Curr. Opin. Psychol. 24, 41–47 (2018). 10.1016/j.copsyc.2018.04.009 29738891

[33] S. A. Rhoads, L. Gan, K. Berluti, K. O’Connell, J. Cutler, P. L. Lockwood, A. A. Marsh, Neurocomputational basis of learning when choices simultaneously affect both oneself and others. Nat. Commun. 16, 9350 (2025). 10.1038/s41467-025-64424-9 PMC12546838 41125577

[34] S. Vahed, A. G. Sanfey, Large-scale community study reveals information sampling drives fairness decisions. Commun. Psychol. 3, 178 (2025). 10.1038/s44271-025-00354-y PMC12660913 41310169

[35] Y. Li, J. Hu, C. C. Ruff, X. Zhou, Neurocomputational evidence that conflicting prosocial motives guide distributive justice. Proc. Natl. Acad. Sci. U.S.A. 119, e2209078119 (2022). 10.1073/pnas.2209078119 PMC9897457 36445964

[36] I. Huijsmans, S. Vahed, C. E. Răţală, A. Llera, A. G. Sanfey, Competing fairness ideals underlie wealth inequality across decision contexts. Sci. Rep. 14, 31882 (2024). 10.1038/s41598-024-83361-z PMC11685827 39738352

[37] M. J. Crockett, Z. Kurth-Nelson, J. Z. Siegel, P. Dayan, R. J. Dolan, Harm to others outweighs harm to self in moral decision making. Proc. Natl. Acad. Sci. U.S.A. 111, 17320–17325 (2014). 10.1073/pnas.1408988111 PMC4260587 25404350

[38] D. T. Schultner, B. R. Lindström, M. Cikara, D. M. Amodio, Transmission of social bias through observational learning. Sci. Adv. 10, eadk2030 (2024). 10.1126/sciadv.adk2030 PMC11212708 38941465

[39] F. Faul, E. Erdfelder, A.-G. Lang, A. Buchner, G* Power 3: A flexible statistical power analysis program for the social, behavioral, and biomedical sciences. Behav. Res. Methods 39, 175–191 (2007). 10.3758/bf03193146 17695343

[40] J. Abeler, A. Falk, L. Goette, D. Huffman, Reference points and effort provision. Am. Econ. Rev. 101, 470–492 (2011).

[41] R. S. Team, RStudio: Integrated development environment for R. *No Title* (2021).

