# Supplementary material for: Psychological and computational evidence that merit is a constructed evaluative process
Source: Sci Adv. 2026 Oct 2;12(40):eaeh5472. doi: 10.1126/sciadv.aeh5472 (PMC13632724; doi:10.1126/sciadv.aeh5472)
Supplement: Supplementary file 1 [file sciadv.aeh5472_sm.pdf]

Science Advances
AAAS

Supplementary Materials for

Psychological and computational evidence that merit is a constructed evaluative process

Sarah Vahed and Alan G. Sanfey

Corresponding author: Sarah Vahed, sarah.vahed@donders.ru.nl

Sci. Adv. **12**, eaeh5472 (2026)
DOI: 10.1126/sciadv.aeh5472

This PDF file includes:

Supplementary Methods
Supplementary Results
Tables S1 to S22
Figs. S1 to S18

Supplementary Methods

S1. Experimental Materials and Questionnaires

S1.1. Tournament task screen

Example Screen of Easy Task Condition.

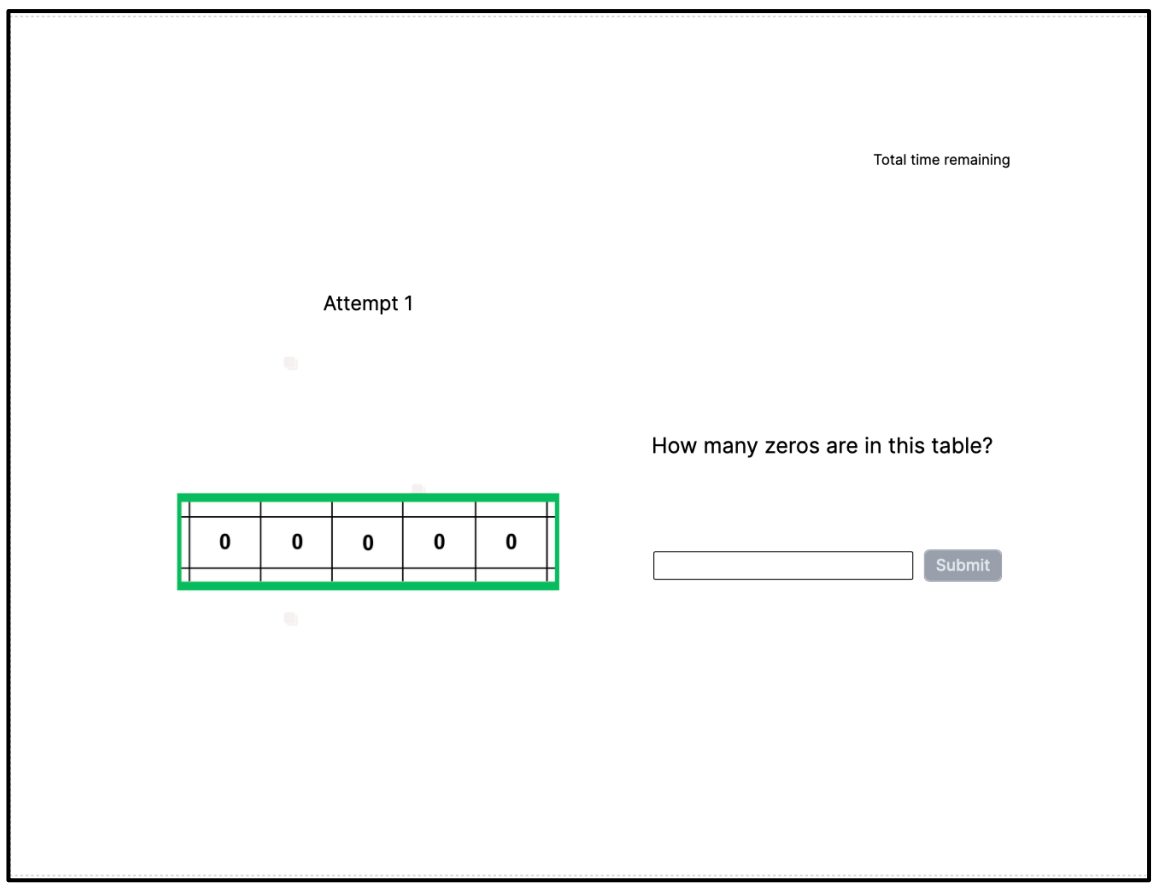

Example Screen of Diffucult Task Condition.

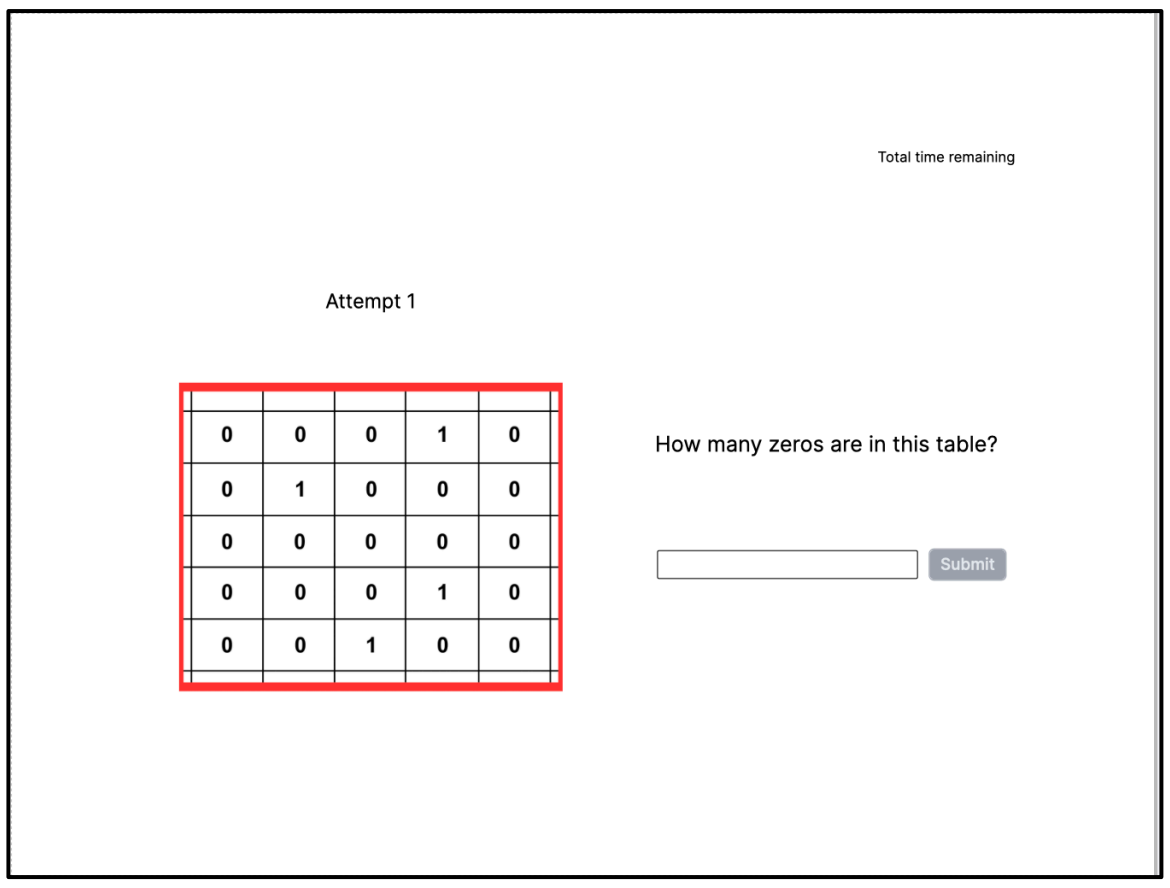

S1.2 Socio-demographic measures (captured in Studies 1, 2, 3, 4)

Your age
Please Select...
18 19 20 21 22 23 24 25 26 27 28 29 30 31 32 33 34 35 36 37 38 39 40 41 42 43 44 45 46 47 48 49 50 51 52 53 54 55 56 57 58 59 60 61 62 63 64 65 66 67 68 69 70 71 72 73 74 75 76 77 78 79 80 81 82 83 84 85 86 87 88 89 90 91 92 93 94 95 96 97 98 99+

Your gender
Female; Male; Other; Prefer not to say

The country in which you were born
Please Select...
Afghanistan, Albania, Algeria, Andorra, Angola, Antigua & Deps, Argentina, Armenia, Australia, Austria, Azerbaijan, Bahamas, Bahrain, Bangladesh, Barbados, Belarus, Belgium, Belize, Benin, Bhutan, Bolivia, Bosnia Herzegovina, Botswana, Brazil, Brunei, Bulgaria, Burkina, Burundi, Cambodia, Cameroon, Canada, Cape Verde, Central African Rep, Chad, Chile, China, Colombia, Comoros, Congo, Congo (Democratic Rep), Costa Rica, Croatia, Cuba, Cyprus, Czech Republic, Denmark, Djibouti, Dominica, Dominican Republic, East Timor, Ecuador, Egypt, El Salvador, Equatorial Guinea, Eritrea, Estonia, Ethiopia, Fiji, Finland, France, Gabon, Gambia, Georgia, Germany, Ghana, Greece, Grenada, Guatemala, Guinea, Guinea-Bissau, Guyana, Haiti, Honduras, Hungary, Iceland, India, Indonesia, Iran, Iraq, Ireland (Republic of), Israel, Italy, Ivory Coast, Jamaica, Japan, Jordan, Kazakhstan,

Kenya, Kiribati, Korea North, Korea South, Kosovo, Kuwait, Kyrgyzstan, Laos, Latvia, Lebanon, Lesotho, Liberia, Libya, Liechtenstein, Lithuania, Luxembourg, Macedonia, Madagascar, Malawi, Malaysia, Maldives, Mali, Malta, Marshall Islands, Mauritania, Mauritius, Mexico, Micronesia, Moldova, Monaco, Mongolia, Montenegro, Morocco, Mozambique, Myanmar (Burma), Namibia, Nauru, Nepal, Netherlands, New Zealand, Nicaragua, Niger, Nigeria, Norway, Oman, Pakistan, Palau, Panama, Papua New Guinea, Paraguay, Peru, Philippines, Poland, Portugal, Qatar, Romania, Russian Federation, Rwanda, St Kitts & Nevis, St Lucia, Saint Vincent & the Grenadines, Samoa, San Marino, Sao Tome & Principe, Saudi Arabia, Senegal, Serbia, Seychelles, Sierra Leone, Singapore, Slovakia, Slovenia, Solomon Islands, Somalia, South Africa, South Sudan, Spain, Sri Lanka, Sudan, Suriname, Swaziland, Sweden, Switzerland, Syria, Taiwan, Tajikistan, Tanzania, Thailand, Togo, Tonga, Trinidad & Tobago, Tunisia, Turkey, Turkmenistan, Tuvalu, Uganda, Ukraine, United Arab Emirates, United Kingdom, United States of America, Uruguay, Uzbekistan, Vanuatu, Vatican City, Venezuela, Vietnam, Yemen, Zambia, Zimbabwe

Country of citizenship
Please Select...
Afghanistan, Albania, Algeria, Andorra, Angola, Antigua & Deps, Argentina, Armenia, Australia, Austria, Azerbaijan, Bahamas, Bahrain, Bangladesh, Barbados, Belarus, Belgium, Belize, Benin, Bhutan, Bolivia, Bosnia Herzegovina, Botswana, Brazil, Brunei, Bulgaria, Burkina, Burundi, Cambodia, Cameroon, Canada, Cape Verde, Central African Rep, Chad, Chile, China, Colombia, Comoros, Congo, Congo (Democratic Rep), Costa Rica, Croatia, Cuba, Cyprus, Czech Republic, Denmark, Djibouti, Dominica, Dominican Republic, East Timor, Ecuador, Egypt, El Salvador, Equatorial Guinea, Eritrea, Estonia, Ethiopia, Fiji, Finland, France, Gabon, Gambia, Georgia, Germany, Ghana, Greece, Grenada, Guatemala, Guinea, Guinea-Bissau, Guyana, Haiti, Honduras, Hungary, Iceland, India, Indonesia, Iran, Iraq, Ireland (Republic of), Israel, Italy, Ivory Coast, Jamaica, Japan, Jordan, Kazakhstan, Kenya, Kiribati, Korea North, Korea South, Kosovo, Kuwait, Kyrgyzstan, Laos, Latvia, Lebanon, Lesotho, Liberia, Libya, Liechtenstein, Lithuania, Luxembourg, Macedonia, Madagascar, Malawi, Malaysia, Maldives, Mali, Malta, Marshall Islands, Mauritania, Mauritius, Mexico, Micronesia, Moldova, Monaco, Mongolia, Montenegro, Morocco, Mozambique, Myanmar (Burma), Namibia, Nauru, Nepal, Netherlands, New Zealand, Nicaragua, Niger, Nigeria, Norway, Oman, Pakistan, Palau, Panama, Papua New Guinea, Paraguay, Peru, Philippines, Poland, Portugal, Qatar, Romania, Russian Federation, Rwanda, St Kitts & Nevis, St Lucia, Saint Vincent & the Grenadines, Samoa, San Marino, Sao Tome & Principe, Saudi Arabia, Senegal, Serbia, Seychelles, Sierra Leone, Singapore, Slovakia, Slovenia, Solomon Islands, Somalia, South Africa, South Sudan, Spain, Sri Lanka, Sudan, Suriname, Swaziland, Sweden, Switzerland, Syria, Taiwan, Tajikistan, Tanzania, Thailand, Togo, Tonga, Trinidad & Tobago, Tunisia, Turkey, Turkmenistan, Tuvalu, Uganda, Ukraine, United Arab Emirates, United Kingdom, United States of America, Uruguay, Uzbekistan, Vanuatu, Vatican City, Venezuela, Vietnam, Yemen, Zambia, Zimbabwe

Which racial category best describes you? [only captured for studies 1, 2, and 3]
Please Select...

American Indian or Alaskan Native
Asian / Pacific Islander
Black or African American
Hispanic White / Caucasian
Multiple ethnicity/Other (please specify)

Your highest level of education
Please Select...
Less than high school diploma
High school degree or equivalent
Bachelor's degree (e.g. BA / BS)
Master's degree (e.g. MA / MSc / MEd)
Doctorate (e.g. PhD)
Other (please specify)

Employment Status
Please Select...
Employed (full-time or part-time)
Unemployed
Student
Retired
Other (please specify)

On a scale from not religious at all to very religious, how religious are you?
Please Select... 1 (Not religious at all) 2 3 4 5 6 (Very religious)

On a scale from very liberal to very conservative, how would you describe your political orientation?
Please Select... 1 (Strongly Liberal) 2 3 4 5 6 7 (Strongly Conservative)

Self-reported socio-economic status, adapted from E. Goodman, N. E. Adler, I. Kawachi, A. L. Frazier, B. Huang, G. A. Colditz, Adolescents' perceptions of social status: Development and evaluation of a new indicator. *Pediatrics* 108, e31 – e31 (2001).

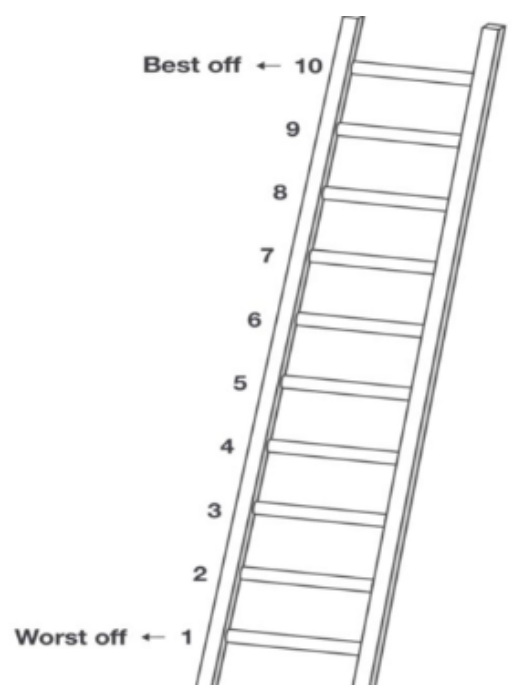

Think of this ladder as representing where people stand in the United States. At the top of the ladder are the people who are best off - those who have the most money, the most education, and the most respected jobs. At the bottom are the people who are the worst off - those who have the least money, least education, the least respected jobs, or no job. The higher up you are on this ladder, the closer you are to the people at the top; the lower you are, the closer you are to the people at the very bottom.

Where would you place yourself on this ladder? Move the slider to the number on the rung where you think you stand at this time in your life relative to other people in the United States.

1 (Worst off) – 10 (Best off)

S1.3 Tournament comprehension and estimation questions (Studies 1, 2, 3 and 4*) [Study 4 bonus=$0.15]

You have the opportunity to earn a $0.75[$0.15] bonus by answering the following question. If your response is within 5 above or below the correct number (i.e. range of 10), you will receive the bonus.

200 Prolific participants recently completed the EASY tables — how many people who completed the EASY tables do you think ended up getting the high reward? (Write your estimate below, choose a number between 0 - 200)

You have the opportunity to earn a $0.75[$0.15] bonus by answering the following question. If your response is within 5 above or below the correct number (i.e. range of 10), you will receive the bonus.

200 Prolific participants completed the EASY tables — on average, how many tables do you estimate these people completed within 9 minutes? (Write your estimate below, choose a number between 0 - 500)

You have the opportunity to earn a $0.75[$0.15] bonus by answering the following question. If your response is within 5 above or below the correct number (i.e. range of 10), you will receive the bonus.

200 Prolific participants completed the DIFFICULT tables — how many people who completed the DIFFICULT tables do you think ended up getting the high reward? (Write your estimate below, choose a number between 0 - 200)

You have the opportunity to earn a $0.75[$0.15] bonus by answering the following question. If your response is within 5 above or below the correct number (i.e. range of 10), you will receive the bonus.

200 Prolific participants completed the DIFFICULT tables — on average, how many tables do you estimate these people completed within 9 minutes? (Write your estimate below, choose a number between 0 - 500)

S1.4 Social Value Orientation (SVO Slider Measure)

Imagine that you have been randomly paired with another person, whom we will refer to as the "other". This other person is someone you do not know and will remain mutually anonymous. All of your choices would be completely confidential.

You will be making a series of decisions about allocating resources between you and this other person. For each of the following questions, please indicate the distribution you prefer most by selecting the button below the payoff allocations. You can only make one selection for each

question. Your decisions will yield money for both yourself and the other person. In the example below, a person has chosen to distribute the payoff so that he/she receives 0.50 dollars, while the anonymous other person receives 0.40 dollars.

There are no right or wrong answers, this is all about personal preferences. After you have made your decision, select the resulting distribution of money by clicking on button below your choice.

Items 1–6 (Primary SVO angle estimation items):

1. (85, 85) to (85, 15)
2. (85, 85) to (15, 85)
3. (50, 100) to (100, 50)
4. (85, 85) to (15, 85)
5. (100, 50) to (50, 100)
6. (85, 85) to (85, 15)

Items 7–15 (Inequality aversion and secondary items):

7. (85, 85) to (85, 15)
8. (85, 85) to (15, 85)
9. (100, 50) to (50, 100)
10. (50, 100) to (100, 50)
11. (85, 85) to (85, 15)
12. (85, 85) to (15, 85)
13. (100, 50) to (50, 100)
14. (50, 100) to (100, 50)
15. (85, 85) to (85, 15)

S1.5 Post-task measures [for Study 4 only] *****also asked in studies 1, 2 and 3***

"I found the tournament required a lot of effort."
Strongly Disagree 0 – 4 Strongly Agree

"I found the tournament was easy to complete in the time given."
Strongly Disagree 0 – 4 Strongly Agree

"I found the tournament challenged my abilities."
Strongly Disagree 0 – 4 Strongly Agree

"For the tournament, I felt good about my performance."
Strongly Disagree 0 – 4 Strongly Agree

"For the tournament, I am satisfied with my overall rank."
Strongly Disagree 0 – 4 Strongly Agree

"I was impressed by my performance in the tournament."

Strongly Disagree 0 – 4 Strongly Agree

How would you rate how fair the tables were assigned during the tournament?
Very Unfair 1 – 5 Very Fair

How out of control did you feel over the outcome of the tournament?
Not in control at all 1 – 5 Completely in control

How would you rate how fair the tournament was?**
Very Unfair 1 – 5 Very Fair

What do you think, did the tournament reward depend on effort or luck? **
0% Effort / (100% Luck) – 100% Effort / 0% Luck

S1.6 Task strategy (Qualitative open response)

The questions below are about Phase 2: The Decision Task.

Can you explain how you made your decisions on how to share the $1 with the other players. For example, what mattered to you most during the decision task (the reward, the tables completed, the group performance, the $1)? Was it easy or difficult to make your decision? Please be as detailed as possible.

S1.7 Basic Social Justice Orientation

The questions below are about your views on American society.

What do you think, it is just if all people have the same living conditions.
Strongly Disagree 1 - 5 Strongly Agree

It is just if income and wealth are equally distributed among the members of our society.
Strongly Disagree 1 - 5 Strongly Agree

A society is just if there are only minor income disparities between people.
Strongly Disagree 1 - 5 Strongly Agree

A society is just if it takes care of those who are poor and needy.
Strongly Disagree 1 - 5 Strongly Agree

It is just if people taking care of their children or their dependent relatives receive special support and benefits.
Strongly Disagree 1 - 5 Strongly Agree

A society is just if all people have sufficient nutrition, shelter, clothing as well as access to education and medical care.
Strongly Disagree 1 - 5 Strongly Agree

It is just if hard working people earn more than others.
Strongly Disagree 1 - 5 Strongly Agree

It is just if every person receives only that which has been acquired through their own efforts.
Strongly Disagree 1 - 5 Strongly Agree

A society is just if differences in income and assets reflect performance differences between people.
Strongly Disagree 1 - 5 Strongly Agree

It is just if members of respectable families have certain advantages in their lives.
Strongly Disagree 1 - 5 Strongly Agree

It is fair if people on a higher level of society have better living conditions than those on the lower level.
Strongly Disagree 1 - 5 Strongly Agree

It is just if people who have achieved good reputation and wealth profit from this later in life.
Strongly Disagree 1 - 5 Strongly Agree

S2. Study 2 Modelling Procedure

S2.1. Coding of predictors

On each of the 80 trials, participants allocated a continuous proportion of a fixed endowment between two tournament players. Each player was characterised by two experimentally defined attributes: structural productivity (Advantaged vs Disadvantaged) and performance (High Performer vs Low Performer). To model decisions as relative evaluations, both attributes were contrast-coded at the trial level as differences between Player 2 and Player 1.

Relative productivity was coded as +1 when Player 2 was Disadvantaged and Player 1 was Advantaged, −1 when Player 2 was Advantaged and Player 1 was Disadvantaged, and 0 when both players shared the same productivity level. Relative performance was coded analogously, with +1 indicating that Player 2 was a High Performer while Player 1 was a Low Performer, −1 indicating the reverse, and 0 indicating matched performance. This coding ensured that zero values corresponded to trials on which the two players were equivalent on the relevant tournament dimensions.

Observed allocations were treated as continuous responses bounded in the unit interval. To ensure numerical stability of the Beta likelihood, responses were clipped away from exact 0 and 1 using a small tolerance (1e-6), preventing undefined likelihood values while preserving the empirical distribution.

S2.2. Core model specification

Participant behaviour was modelled using a continuous latent-value framework in which trial-wise allocations were governed by a subjective value signal derived from relative structural productivity and performance. For a given participant, subjective value on trial (t) was defined as a linear combination of the contrast-coded predictors:

$$\Delta V\_t = w\_productivity \cdot s\_productivity,t + w\_performance \cdot s\_performance,t,$$

where *w_productivity* and *w_performance* are free parameters capturing individual sensitivity to relative productivity and relative performance, respectively. To allow for individual differences in how strongly value differences governed choice behaviour, the value signal was modulated by an auxiliary sensitivity-attenuation parameter $\gamma \in (0,1)$, yielding the scaled value $(1 - \gamma)\cdot\Delta V\_t$. Larger values of γ pull expected allocations closer to equal division by reducing the influence of value differences on the allocation function. The modulated value signal was mapped to an expected allocation μ_t using a logistic function. Observed allocations were assumed to arise from a Beta distribution parameterised in mean-precision form, with μ_t defining the mean and a fixed precision/noise parameter $\kappa = 30$ controlling dispersion around the mean. In this regard, we fixed $\kappa = 30$ as it corresponds to an expected SD of approximately 0.09 at $\mu = .50$, corresponding to approximately one allocation step on the slider (0.10).

S2.3. Parameter estimation

Model parameters were estimated independently for each participant using maximum likelihood estimation. Optimisation was performed using the L-BFGS-B algorithm with box constraints on all parameters. Sensitivity parameters were constrained to a finite range to prevent implausible values, and the parameter γ was restricted to the open interval (0,1).

To mitigate the risk of convergence to local minima, a multi-start optimisation procedure was employed. For the full model, 1,000 random initialisations were drawn per participant, spanning the admissible parameter space. The solution yielding the lowest negative log-likelihood was retained as the final estimate. Reduced models were fit using analogous procedures with the same restart counts (n = 1,000).

Participants whose allocation behaviour exhibited effectively zero variance across trials were classified as 'constant responders'. For these participants, optimisation was bypassed and behaviour was instead modelled using a zero-parameter null/equality model with a fixed expected allocation of $\mu = 0.5$. These participants were retained in all subsequent analyses to avoid selection bias.

Model fit was quantified using the negative log-likelihood as well as information criteria (AIC and BIC), computed using the number of free parameters and the number of trials per participant. To provide a conservative model comparison, we used (k=3) for the full model and (k=2) for each single-cue model, counting gamma despite its lack of independent identifiability. Results were unchanged when using (k=2) and (k=1), respectively, reflecting the models' identifiable parameters.

S2.4. Parameter recovery

To assess identifiability of model parameters, parameter recovery analyses were conducted using simulated data generated from the full/integrated model. Synthetic datasets were generated by sampling known values for w_productivity, w_performance, and γ from broad uniform distributions ($w \in [-3, 3]$, $\gamma \in (0, 1)$), and simulating trial-wise allocations using the same contrast-coded design structure as in the empirical data.

For each simulated participant, allocations were generated by computing the expected allocation μ_t from the known parameters and drawing responses from a Beta distribution with fixed precision, κ. The model was then refit to the simulated data using the same model specification and estimation procedure as in the empirical analyses, with optimisation restarts set at $n = 200$.

Recovery accuracy was assessed by computing Pearson correlations between true and recovered parameter values across simulated participants. High correspondence between true and recovered parameters was taken as evidence that the model parameters were identifiable given the experimental design and number of trials.

S2.5. Model comparison

In addition to the full model incorporating both productivity and performance sensitivity, three reduced models were fit to each participant's data. The productivity-only model included *w_productivity* and γ, with weight on *w_performance* fixed to zero. The performance-only model included *w_performance* and γ, with weight on *w_ productivity* fixed to zero. As in the full model, participants exhibiting near-zero response variance were treated as constant responders and evaluated under a null/equality model. This null model assumed no sensitivity to either dimension and fixed the expected allocation to $\mu = 0.5$ on all trials.

Model comparison was conducted at the participant level. Information criteria (AIC and BIC) were computed for each model, and within-participant differences in AIC and BIC were calculated relative to the null model. Likelihood-ratio tests were used to compare the full model against each reduced model, with degrees of freedom corresponding to differences in the number of free parameters.

At the group level, model performance was summarised by computing the proportion of participants for whom each model yielded the lowest BIC, providing an estimate of the relative frequency with which each model best accounted for individual behaviour. To provide a conservative model comparison, we used (k=3) for the full model and (k=2) for each single-cue model, counting gamma despite its lack of independent identifiability. Results were unchanged when using (k=2) and (k=1), respectively.

S2.6. Model recovery

We conducted a model recovery analysis to assess whether the candidate models were distinguishable given the empirical design. Synthetic datasets were generated from each candidate model and all competing models were subsequently fit to those data using the same model specifications and comparison criteria as in the empirical analyses.

Simulated data were generated using the pooled empirical trial structure, preserving the observed combinations of relative productivity and relative performance across trials. Four models were considered: a null model assuming stable equal allocations, a productivity-only model, a performance-only model, and a full (integrated) model incorporating both productivity and performance. For each generating model, 30 independent simulations were performed. For each simulation, model-specific parameters were sampled from broad uniform distributions (sensitivity parameters in [−2, 2]; parameter γ in [0.1, 0.9]), and expected allocations were computed using the same latent value formulation and logistic mapping as in

the empirical model. Observed allocations were then sampled from a Beta distribution with fixed precision ($\kappa = 30$).

Each simulated dataset was fit with all four models (10 random initialisations per model), and model comparison was performed at the individual-dataset level using BIC. Recovery performance was summarised by computing, for each generating model, the proportion of simulations in which each candidate model was selected as the best-fitting model. These proportions were organised into a confusion matrix, allowing assessment of whether each model was reliably identified when it generated the data.

S2.7. Held-out prediction

To evaluate the out-of-sample predictive performance of the candidate models, we conducted held-out predictive checks at the participant level. For each participant, trials were randomly split into a training set (70% of trials) and a held-out test set (30% of trials). This procedure was performed once per participant. All models were fit exclusively on the training data using the same estimation procedures and parameter constraints as in the main analyses.

Four models were evaluated: a null model assuming stable equal allocations, a productivity-only model, a performance-only model, and a full model incorporating both productivity and performance. For each fitted model, expected allocations on the held-out trials were computed using the estimated parameters and the corresponding latent value formulation. Predicted means were mapped through the same logistic function and evaluated under a Beta likelihood with fixed precision ($\kappa = 30$), yielding a test log-likelihood for each model.

Predictive performance was quantified by computing, within each participant, the difference in test log-likelihood between each model and the null model. These within-participant differences reflect the extent to which each model generalised to unseen data beyond a stable equality heuristic. Group-level summaries were obtained by averaging these differences across participants and by computing the proportion of participants for whom each model achieved higher test log-likelihood than the null model. Participants exhibiting invariant allocation behaviour (‘constant responders’) do not provide meaningful held-out predictive signal, as all models reduce to the equality heuristic for these individuals. Held-out predictive analyses therefore characterise generalisation performance among participants whose behaviour varies as a function of tournament information.

S2.8. Clustering of decision regimes (strategies)

To characterise heterogeneity in decision-making, we classified participants into decision regimes based on their fitted raw model parameters. For each participant, we evaluated both the magnitude and sign of the performance and productivity sensitivity parameters ($w_{performance}$, $w_{productivity}$). A threshold of $|w| \geq 1$ was applied to identify participants whose exhibited non-trivial latent sensitivities, whereas participants with parameter magnitudes below this threshold were treated as (weakly) insensitive to that dimension in the latent parameter space.

Based on these criteria, participants were classified into five categories. Individuals were labelled ‘Null’ when both productivity sensitivity and performance sensitivity lay between −1 and 1. Participants were classified as ‘Performance-dominant’ when productivity

sensitivity lay between −1 and 1 and performance sensitivity was at least +1, and as 'Performance-lenient' when productivity sensitivity lay between −1 and 1 and performance sensitivity was at most −1. Participants were classified as 'Corrective' when productivity sensitivity was at least +1, and as 'Legitimising' when productivity sensitivity was at most −1. In both mixed categories, performance sensitivity could take any value. That is, any participant with productivity sensitivity of magnitude 1 or greater was assigned to a mixed category, regardless of their performance sensitivity.

To assess the robustness of this theory-guided classification, we compared the resulting regimes with clusters obtained from an independent, data-driven k-means analysis applied directly to behavioural allocation patterns. Specifically, for each participant, mean allocations were computed per player pairing. To guide the determination of the optimal number of clusters for this response-based analysis, we evaluated the within-cluster sum of squares (WSS) and Silhouette criteria, and a four-cluster solution was retained. K-means clustering was then performed to classify participants based on their response patterns, using 10,000 random starting point iterations. To enhance reproducibility, a consistent random seed was used for this clustering. Cluster profiles were summarised using cluster-level means and standard errors for each pairing. Furthermore, as our primary interest was in latent sensitivities, we clustered participants based on raw parameters; however, we also repeated the clustering using effective sensitivities, defined as $(1-\gamma) \times w$, and found that the raw-sensitivity threshold of $|w| \geq 1$ corresponded approximately to an effective-sensitivity threshold of $|(1-\gamma) \times w| \geq 0.70$.

S3. Study 3 and 4 Modeling Procedure

S3.1. Coding of predictors

On each trial, participants allocated a proportion of a fixed endowment ($1) between themselves and a single tournament player. The receiver was characterised by two experimentally defined attributes: structural productivity (Advantaged vs Disadvantaged) and performance (High Performer vs Low Performer). These attributes were encoded as binary predictors describing the observed player. Productivity was coded as +1 when the player was Disadvantaged and −1 when the player was Advantaged. Performance was coded as +1 when the player was a High Performer and −1 when the player was a Low Performer. Allocations were treated as continuous responses bounded in the unit interval. To ensure numerical stability of the Beta likelihood, responses were clipped away from exact 0 and 1 using a small tolerance (1e−6). ***Core model specification, Parameter estimation, Parameter recovery, Model comparison, and Clustering of decision regimes followed the same procedure as study 2.***

S3.2. Model recovery

We conducted a model recovery analysis to assess whether the candidate models were distinguishable given the empirical design. Synthetic datasets were generated from each candidate model and all competing models were subsequently fit to those data using the same model specifications and comparison criteria as in the empirical analyses.

Simulated data were generated using the pooled empirical trial structure, preserving the combinations of relative productivity and relative performance across trials, in order to test

theoretical model separability. Four models were considered: a null model assuming stable equal allocations, a productivity-only model, a performance-only model, and a full model incorporating both productivity and performance. For each generating model, 30 independent simulations were performed. For each simulation, model-specific parameters were sampled from broad uniform distributions (sensitivity parameters in $[-2, 2]$; parameter γ in $[0.1, 0.9]$), and expected allocations were computed using the same latent value formulation and logistic mapping as in the empirical model. Observed allocations were then sampled from a Beta distribution with fixed precision ($\kappa = 30$).

Each simulated dataset was fit with all four models (10 random initialisations per model), and model comparison was performed at the individual-dataset level using BIC. Recovery performance was summarised by computing, for each generating model, the proportion of simulations in which each candidate model was selected as the best-fitting model. These proportions were organised into a confusion matrix, allowing assessment of whether each model was reliably identified when it generated the data. This procedure was then repeated using the reduced participant-level trial structures.

S3.3. Held-out prediction

To evaluate the out-of-sample predictive performance of the candidate models, we conducted held-out predictive checks at the participant level. For each participant, trials were randomly split into a training set (70% of trials) and a held-out test set (30% of trials). This split was performed once per participant. All models were fit exclusively on the training data using the same estimation procedures and parameter constraints as in the main analyses (but with reduced restarts of 10). Four models were evaluated: a null model assuming stable equal allocations, a productivity-only model, a performance-only model, and a full model incorporating both productivity and performance. For each fitted model, expected allocations on the held-out trials were computed using the estimated parameters and the corresponding latent value formulation. Predicted means were mapped through the same logistic function and evaluated under a Beta likelihood with fixed precision ($\kappa = 30$), yielding a test log-likelihood for each model.

Predictive performance was quantified by computing, within each participant, the difference in test log-likelihood between each model and the null model. These within-participant differences reflect the extent to which each model generalised to unseen data beyond a stable equality heuristic. Group-level summaries were obtained by averaging these differences across participants and by computing the proportion of participants for whom each model achieved higher test log-likelihood than the null model. Participants exhibiting invariant allocation behaviour (constant responders) do not provide meaningful held-out predictive signal, as all models reduce to the equality heuristic for these individuals. Held-out predictive analyses therefore characterise generalisation performance among participants whose behaviour varies as a function of tournament information.

Supplementary Results

S4. Sample Characteristics

Supplementary Table S1. Study 1 (*N*=124) demographic characteristics of participants, including age, gender, education level, political orientation, race, religious affiliation and self-reported SES.

Demographics	Categories	Frequency (n)	Percentage (%)
Age	Mean (Range)	38.97 (18 – 78)	-
Gender	Male	53	42.7
	Female	69	55.6
	Other	2	1.61
Education	High School Diploma	49	39.5
	Bachelor's Degree	48	38.7
	Master's Degree	18	14.5
	Doctorate	2	1.61
	Other	7	5.65
Political Orientation	1 (Strongly Liberal)	15	12.1
	2	24	19.4
	3	19	15.3
	4	22	17.7
	5	21	16.9
	6	12	9.68
	7 (Strongly Conservative)	11	8.87
Race	Asian/Pacific Islander	7	5.65
	Black/African American	33	26.6
	Caucasian/White	76	61.3
	Hispanic	6	4.84
	Multiple Ethnicities/Other	2	1.61
Religion	1 (Not religious at all)	38	30.6
	2	9	7.26
	3	22	17.7
	4	23	18.5
	5	22	17.7
	6 (Very religious)	10	8.06
MacArthur Scale of Subjective Social Status	1 (Worst off in society)	0	0
	2	11	8.87
	3	19	15.3
	4	14	11.3
	5	30	24.2
	6	28	22.6
	7	12	9.68
	8	6	4.84
	9	1	0.81
	10 (Best off in society)	3	2.42

Supplementary Table S2. Study 2 (N=122) demographic characteristics of participants, including age, gender, education level, political orientation, race, religious affiliation and self-reported SES.

**Demographics**	**Categories**	**Frequency (n)**	**Percentage (%)**
Age	Mean (Range)	37.37 (18 – 72)	-
Gender	Male	60	49.2
	Female	62	50.8
Education	High School Diploma	26	21.3
	Bachelor's Degree	52	42.6
	Master's Degree	29	23.8
	Doctorate	13	10.7
	Other	2	1.64
Political Orientation	1 (Strongly Liberal)	19	15.6
	2	22	18.0
	3	8	6.56
	4	15	12.3
	5	19	15.6
	6	23	18.9
	7 (Strongly Conservative)	16	13.1
Race	Asian/Pacific Islander	4	3.28
	Black/African American	33	27.0
	Caucasian/White	79	64.8
	Hispanic	4	3.28
	Multiple Ethnicities/Other	2	1.64
Religion	1 (Not religious at all)	24	19.7
	2	8	6.56
	3	9	7.38
	4	28	23.0
	5	34	27.9
	6 (Very religious)	19	15.6
MacArthur Scale of Subjective Social Status	1 (Worst off in society)	1	0.82
	2	5	4.10
	3	6	4.92
	4	13	10.7
	5	21	17.2
	6	39	32.0
	7	24	19.7
	8	9	7.38
	9	4	3.28
	10 (Best off in society)	0	0

Supplementary Table S3. Study 3 (N=125) demographic characteristics of participants, including age, gender, education level, political orientation, race, religious affiliation and self-reported SES.

Demographics	Categories	Frequency (n)	Percentage (%)
Age	Mean (Range)	40.34 (21 – 80)	-
Gender	Male	64	51.2
	Female	61	48.8
Education	Less than high school diploma	2	1.6
	High School Diploma	41	32.8
	Bachelor's Degree	59	47.2
	Master's Degree	18	14.4
	Doctorate	2	1.6
	Other	3	2.4
Political Orientation	1 (Strongly Liberal)	22	17.6
	2	24	19.2
	3	16	12.8
	4	21	16.8
	5	15	12
	6	14	11.2
	7 (Strongly Conservative)	13	10.4
Race	Asian/Pacific Islander	1	0.8
	Black/African American	21	16.8
	Caucasian/White	89	71.2
	Hispanic	8	6.4
	Multiple Ethnicities/Other	6	4.8
Religion	1 (Not religious at all)	51	40.8
	2	13	10.4
	3	14	11.2
	4	14	11.2
	5	14	11.2
	6 (Very religious)	19	15.2
MacArthur Scale of Subjective Social Status	1 (Worst off in society)	1	0.8
	2	7	5.6
	3	11	8.8
	4	29	23.2
	5	20	16
	6	23	18.4
	7	26	20.8
	8	4	3.2
	9	2	1.6
	10 (Best off in society)	2	1.6

Supplementary Table S4. Study 4 (*N*=388) demographic characteristics of participants, including age, gender, education level, political orientation, religious affiliation and self-reported SES.

**Demographics**	**Categories**	**Frequency (n)**	**Percentage (%)**
Age	Mean (Range)	39.97 (18 – 83)	-
Gender	Male	198	51.0
	Female	184	47.4
	Other	5	1.29
	Prefer not to say	1	0.26
Education	Less than high school diploma	4	1.03
	High School Diploma	127	32.7
	Bachelor's Degree	138	35.6
	Master's Degree	82	21.1
	Doctorate	12	3.09
	Other	25	6.44
Political Orientation	1 (Strongly Liberal)	60	15.5
	2	63	16.2
	3	50	12.9
	4	82	21.1
	5	46	11.9
	6	41	10.6
	7 (Strongly Conservative)	46	11.9
Religion	1 (Not religious at all)	129	33.2
	2	56	14.4
	3	44	11.3
	4	58	14.9
	5	52	13.4
	6 (Very religious)	49	12.6
MacArthur Scale of Subjective Social Status	1 (Worst off in society)	6	1.55
	2	21	5.41
	3	54	13.9
	4	57	14.7
	5	73	18.8
	6	66	17.0
	7	64	16.5
	8	36	9.28
	9	6	1.55
	10 (Best off in society)	5	1.29

S5. Tournament Subjective Perceptions

Subjective Control

Players' perceptions of control over the outcome of the tournament were assessed using the question: "How out of control did you feel over the outcome of the tournament?" (*1 = Not in control at all, 5 = Completely in control*). Participants in the Disadvantaged condition reported significantly lower perceived control (*M*=1.95, SD=1.19) than those in the Advantaged condition (*M*=3.02, SD=1.27), indicating that participants assigned to the 'Difficult' tables felt less able to influence the tournament outcome (W=27572, *p*< .001).

Effort-Luck Attribution

We examined players' perceptions of whether rewards reflected effort or luck (*0% Effort (100% Luck) – 100% Effort (0% Luck))*. Participants in the Disadvantaged condition attributed outcomes significantly more to luck (*M*=42.0%, SD=35.3, Median=27.5%, IQR=65) than participants in the Advantaged condition (*M*=61.3%, SD=30.3, Median=65.5%, IQR=53.8), indicating that players presented with 'Difficult' tables felt the tournament was more a reflection of luck, while those with 'Easy' tables felt the tournament was more a reflection of effort (W=24940, *p*<.001).

Perceived Fairness

Players' perceptions of tournament fairness were analysed on a scale from *1 (Very Unfair) – 5 (Very Fair).* Participants in the disadvantaged condition rated the tournament as significantly less fair (*M*=1.84, SD=1.06, Median=1), than participants in the Advantaged condition (M=3.18, SD=1.30, Median=3), indicating that the same structure was experienced as unjust by those facing constrained opportunities, but not as overtly unfair by those benefiting from advantage (W=29350, *p*<.001).

Effort-Reward Imbalance

To quantify effort-reward imbalance, we computed an effort-reward (ER) ratio per player. Effort, as numerator, was calculated through the sum score of the three perceived effort questions (e.g. "*I found the tournament was easy to complete in the time given*", etc.) and, reward, as denominator, was captured through the sum score of three perceived reward questions (e.g. "*I am satisfied with my overall ranking*", etc.), adapted from N. Van Vegchel, J. De Jonge, H. Bosma, W. Schaufeli, Reviewing the effort–reward imbalance model: drawing up the balance of 45 empirical studies. *Social science & medicine,* 60(5), 1117-1131 (2005), and J. Siegrist, The effort–reward imbalance model. *The handbook of stress and health: A guide to research and practice*, 24-35 (2017) (See Supplementary Methods *(Questionnaires)*):

$$ER = \frac{E}{R \: x \: c} \quad (1)$$

where *E* is the effort score, *R* the reward score, and *c* a correction factor that adjusts for unequal number of items of the effort and reward scores[16]. In this study, *c* always equals 1. For ER < 1, there are less efforts for reward, and ER > 1 reflects more efforts for reward.

Perceived effort (*E)* did not differ between conditions. Participants in the Advantaged condition (*M*=19.50, SD=3.04) and the Disadvantaged condition (*M*=19.30, SD=2.68) reported similar levels of effort, and Wilcoxon rank-sum test indicated no statistically significant difference between groups (W=19,498, *p*=.536). In contrast, perceived reward (*R*) differed substantially between conditions. Participants in the Advantaged condition reported significantly higher perceived rewards (*M*=21.40, SD=3.44) than participants in the Disadvantaged condition (*M*=15.20, SD=3.97). This difference was statistically significant according to a Wilcoxon rank-sum test (W=32,854, *p*< .001). Finally, the effort–reward ratio differed markedly between conditions. Participants in the Disadvantaged condition perceived a substantially greater imbalance between effort and reward (*M*=1.35, SD=0.39) compared with participants in the Advantaged condition (*M*=0.93, SD=0.21). This difference was statistically significant (W=6,142, *p*<.001).

Taken together, these findings indicate that participants perceived the effort required to complete the task as comparable across conditions but experienced the reward structure differently. While perceived effort did not differ between groups, participants in the Disadvantaged condition reported substantially lower perceived rewards, resulting in a significantly higher perceived effort–reward imbalance. In other words, participants in the harder task condition experienced the tournament as requiring more effort relative to the rewards they received.

S6. Study 1 Results

Supplementary Table S5. Study 1 - Role-Specific Generalised Linear Mixed-Effects Model Predicting Allocation

**Model specification: Beta GLMM (logit link); random intercepts for Participant (N = 124; observations = 4,960)**	**Estimate**	**Std. Error**	**Z value**	**Pr(>|z|)**
(Intercept)	0.03814	0.06968	0.547	0.5841
Player1_StructuralProductivityDisadvantaged	-0.56464	0.04253	-13.276	<2e-16 ***
Player1_PerformanceLowPerformer	0.66004	0.03513	18.787	<2e-16 ***
Player2_StructuralProductivityDisadvantaged	0.58375	0.05615	10.396	<2e-16 ***
Player2_PerformanceLowPerformer	-0.83106	0.05835	-14.242	<2e-16 ***
Player1_StructuralProductivityDisadvantaged:Player1_PerformanceLowPerformer	0.16713	0.06744	2.478	0.0132 *
Player2_StructuralProductivityDisadvantaged:Player2_PerformanceLowPerformer	-0.07282	0.06785	-1.073	0.2832

Significance codes: 0 '***' 0.001 '**' 0.01 '*' 0.05 '.' 0.1 ' ' 1

S7. Study 2 Results

Supplementary Table S6. Study 2 - Role-Specific Generalised Linear Mixed-Effects Model Predicting Allocation

**Model specification: Beta GLMM (logit link); random intercepts for Participant (N = 122; observations = 9,760)**	**Estimate**	**Std. Error**	**Z value**	**Pr(>|z|)**
(Intercept)	0.02888	0.033887	0.853	0.39372
Player1_StructuralProductivityDisadvantaged	0.15838	0.03397	4.663	3.12e-06 ***
Player1_PerformanceLowPerformer	0.85679	0.03434	24.950	<2e-16 ***
Player2_StructuralProductivityDisadvantaged	-0.14071	0.03409	-4.128	3.67e-05 ***
Player2_PerformanceLowPerformer	-0.90595	0.03447	-26.286	<2e-16 ***
Player1_StructuralProductivityDisadvantaged:Player1_PerformanceLowPerformer	-0.18890	0.04818	-3.921	8.83e-05 ***
Player2_StructuralProductivityDisadvantaged:Player2_PerformanceLowPerformer	0.13683	0.04822	2.837	0.00455 **

Significance codes: 0 ‘***’ 0.001 ‘**’ 0.01 ‘*’ 0.05 ‘.’ 0.1 ‘ ’ 1

Supplementary Table S7. Study 2 Model Comparison Winning Table

Participant-level model comparison winning table using Bayesian Information Criterion (BIC).

**Model**	**Win Rate***	**Mean ∆BIC**	**Median ∆BIC**
Full model (prod + perf)	0.746	1.29	0
Null model	0.098	2110	341
Performance only	0.131	870	51.9
Productivity only	0.123	843	136

*Win rate reflects the proportion of participants for whom a given model achieved or tied for the lowest BIC (ΔBIC = 0). Mean and median ΔBIC values are computed relative to each participant's best-fitting model; lower values indicate better fit. The full model including both structural productivity and performance sensitivities was most frequently the best-fitting model. The full model was assigned (k=3) for the AIC/BIC calculations; model win rates were robust when (k=2) was used instead.

Consistent with the above, likelihood-ratio tests provided further strong evidence in favour of the integrated model. Compared with the null model, the full model yielded a significantly better fit for 91.0% of participants ($p < .05$), with 88.5% showing $p < .001$. Importantly, the integrated model also outperformed each reduced model for the majority of participants, with significant improvements over the productivity-only model in 82.8% of participants (77.0% at $p < .001$) and over the performance-only model in 81.1% of participants (73.8% at $p < .001$), indicating that both dimensions contributed independently to explaining behaviour. Consistent with these results, participant-level BIC model selection showed that the integrated model was the best-fitting model for 74.6% of participants. In contrast, the null model, performance-only model, and productivity-only model accounted for behaviour in much smaller subsets (9.8%, 9.8%, and 9.0%, respectively). The full model was assigned (k=3) for the AIC/BIC calculations; model win rates were robust when (k=2) was used instead. This pattern indicates that most participants simultaneously incorporated productivity and performance when forming their decisions, rather than relying on a single dimension or a simple equality rule.

Supplementary Figure S1. Study 2 Parameter Recovery

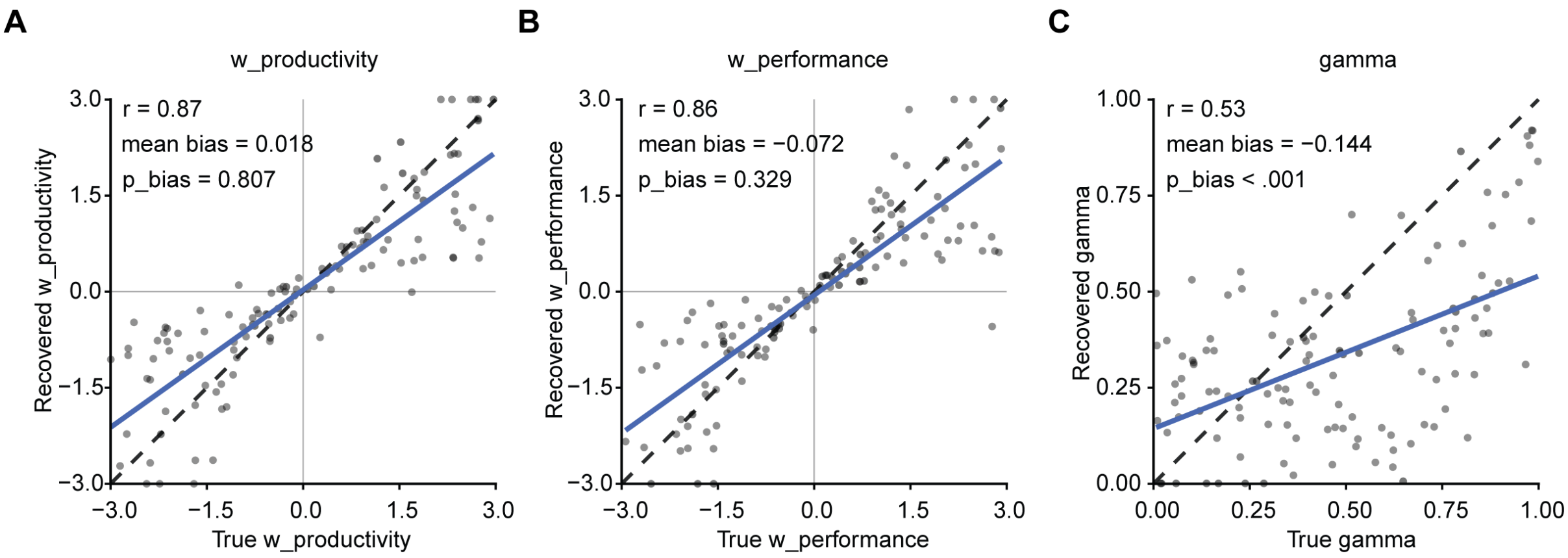

Fig. S1. Study 2 Parameter Recovery. (A) Structural Productivity (*w_productivity)* Recovery. Simulated datasets were generated from known values of raw structural productivity sensitivity, after which the model was refit to recover each parameter. **(B) Performance (*w_performance)* Recovery**. Simulated datasets were generated from known values of raw performance sensitivity, after which the model was refit to recover each parameter. **(C) Senstivity-attentuation (*gamma)* Recovery.** Simulated datasets were generated from known values of sensitivity-attenuation parameter, after which the model was refit to recover each parameter. For A-C, dashed lines indicate perfect recovery (y = x), points represent individual simulations and solid lines indicate the fitted linear recovery relationship. Structural productivity and performance sensitivity showed higher recovery (*w_productivity*: r = 0.87, 95% CI [0.82, 0.91], p<0.001; *w_performance*: r = 0.86, 95% CI [0.80, 0.90], p<0.001), than recovery of gamma (r = 0.53, 95% CI [0.39, 0.64], p<0.001). Bias tests assessed whether recovery error (defined as recovered minus true), differed significantly from zero. These tests showed no evidence of systematic bias for *w_productivity* (mean bias = 0.018, 95% CI [-0.131, 0.167], t(124) = 0.24, p = 0.807) or *w_performance* (mean bias = -0.072, 95% CI [-0.217, 0.073], t(124) = -0.98, p = 0.329), but indicated significant under-recovery of gamma (mean bias = -0.144, 95% CI [-0.190, -0.098], t(124) = -6.22, p<0.001). In-panel statistics report the correlation between true and recovered parameters (r), and mean recovery bias with p-value.

Supplementary Figure S2. Study 2 Gamma Simulations and Distribution

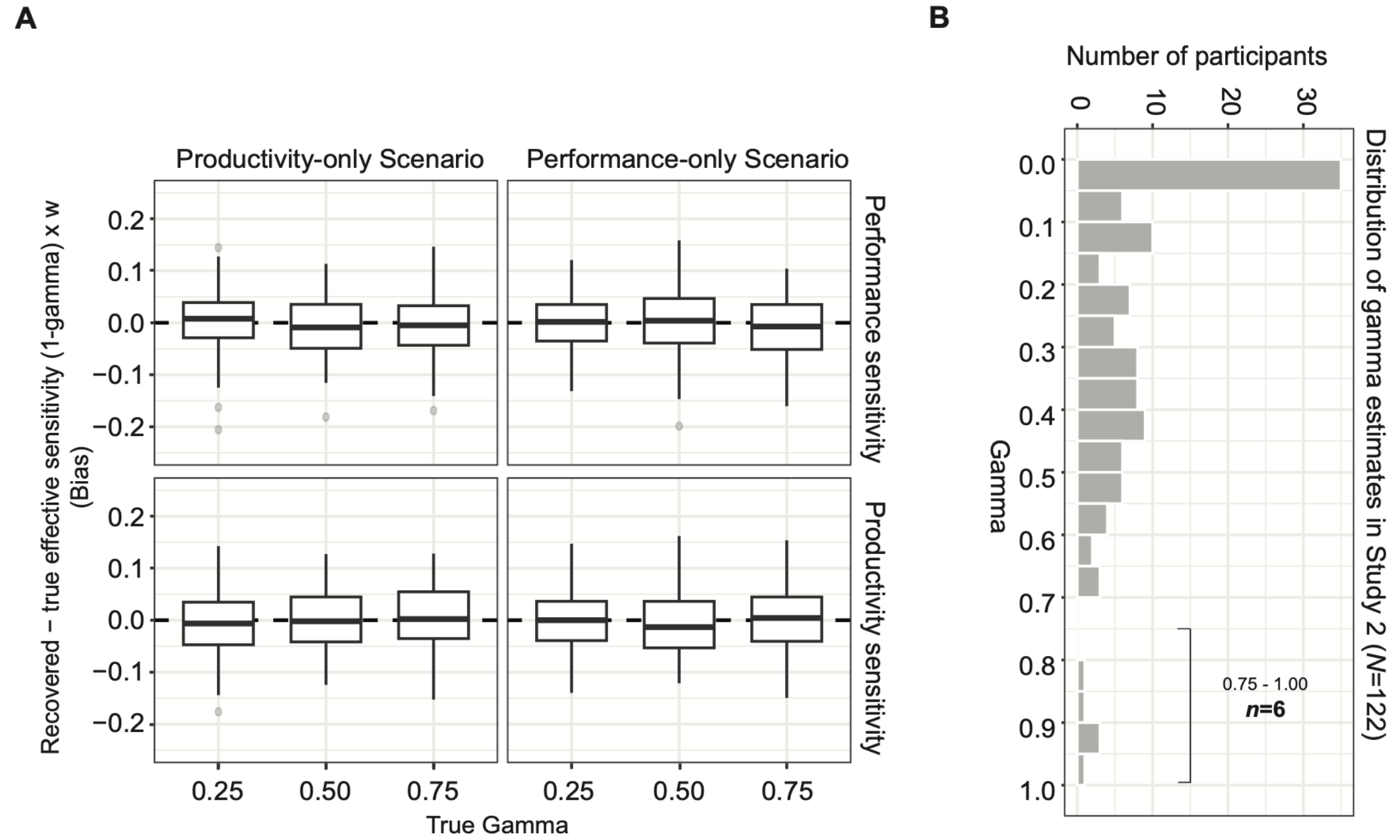

Fig. S2. Study 2 Robustness of sensitivity recovery across gamma levels. (**A**) **Gamma Simulations.** Simulations were run to test whether weaker recovery of gamma biased the behaviourally expressed sensitivity estimates, defined in line with our model as: (1 - *gamma*) x *w* ('*effective sensitivities*'). Simulated datasets were generated for a productivity-only scenario (i.e. simulated data in which effective productivity sensitivity was fixed at 0.75 and effective performance sensitivity was fixed at 0), and a performance-only scenario (i.e. simulated data in which effective performance sensitivity was fixed at 0.75 and effective productivity sensitivity was fixed at 0) across three true values of gamma (0.25, 0.50, and 0.75). Boxplots show recovery bias, defined as recovered minus true sensitivity. The dashed horizontal line indicates zero bias. In the productivity-only scenario, mean bias in effective productivity sensitivity remained close to zero (-0.004, -0.003, 0.006), as did mean bias in the absent performance sensitivity (0.004, -0.005, -0.006). Similarly, in the performance-only scenario, mean bias in effective performance sensitivity remained small (-0.002, 0.0004, -0.009), as did mean bias in the absent productivity sensitivity (-0.0001, -0.008, 0.002). Thus, although gamma itself was underestimated at higher true values, this did not materially bias the behaviourally expressed productivity or performance sensitivity estimates. (**B**) **Gamma Distribution.** Distribution of actual gamma estimates in Study 2 (*N*=122), with 6 participants in this sample (4.92% of total sample) having a gamma higher than 0.75.

Supplementary Figure S3. Study 2 Model Recovery Confusion Matrix

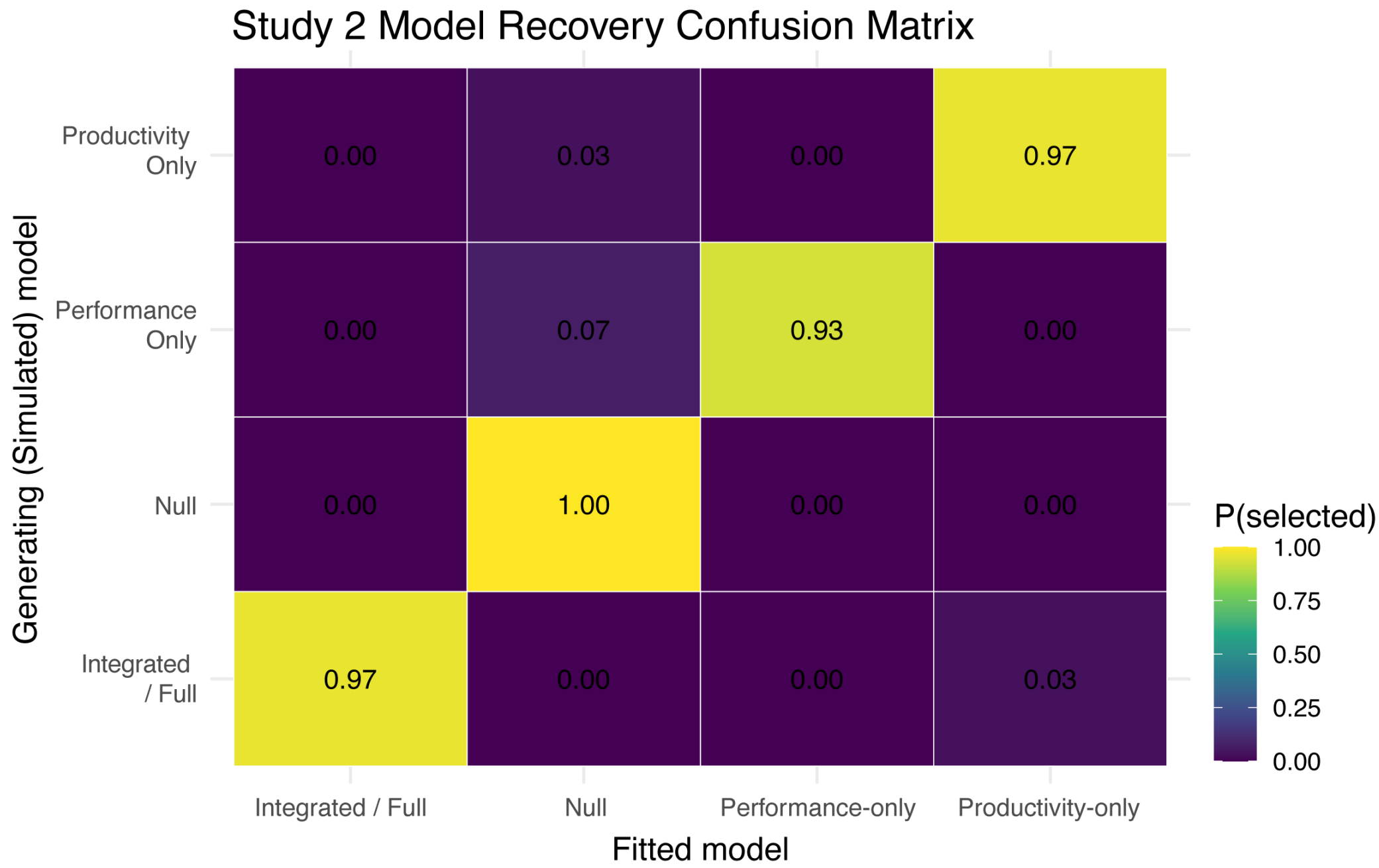

Fig. S3. Study 2 Model recovery confusion matrix. Rows indicate the true data-generating model and columns indicate the model selected as best-fitting based on BIC. Cell values and colour intensity represent the proportion of simulations in which each fitted model was selected for a given generating model. Simulations were conducted using the entire pooled empirical trial structure (30 simulations per model), with model selection performed after fitting all candidate models to each simulated dataset. High values indicate reliable recovery of the true data-generating model.

Interpretation Note: To ensure that the four candidate models were distinguishable under the empirical task structure, we conducted model-recovery simulations in which synthetic datasets were generated from each model using realistic parameter values and the pooled empirical trial structure. The recovered model was identified using BIC. Datasets generated from the integrated model were correctly identified as such in 96.7% of cases, and those generated from the productivity-only and performance-only models were correctly recovered in 96.7% and 93.3% of simulations, respectively. The null model was recovered perfectly (100%). Misclassification rates were low and occurred primarily between adjacent models (e.g. 3.3% confusion between integrated and performance-only models; 3.3% between productivity-only and null; 6.7% between performance-only and null). Overall, model-recovery simulations demonstrate that the modelling space is identified under the full empirical task structure. As an additional check, we repeated model recovery using reduced participant-level empirical trial structures. In this analysis, using 100 simulations per model and sensitivity parameters sampled from [-3, 3], the integrated, performance-only, productivity-only, and null models were recovered in 78%, 78%, 85%, and 100% of simulations, respectively. Misclassifications primarily reflected BIC selection of the null model for one-dimensional sensitivity models, rather than confusion between productivity and performance sensitivity.

Supplementary Figure S4: Study 2 Held-out predictive performance of candidate models

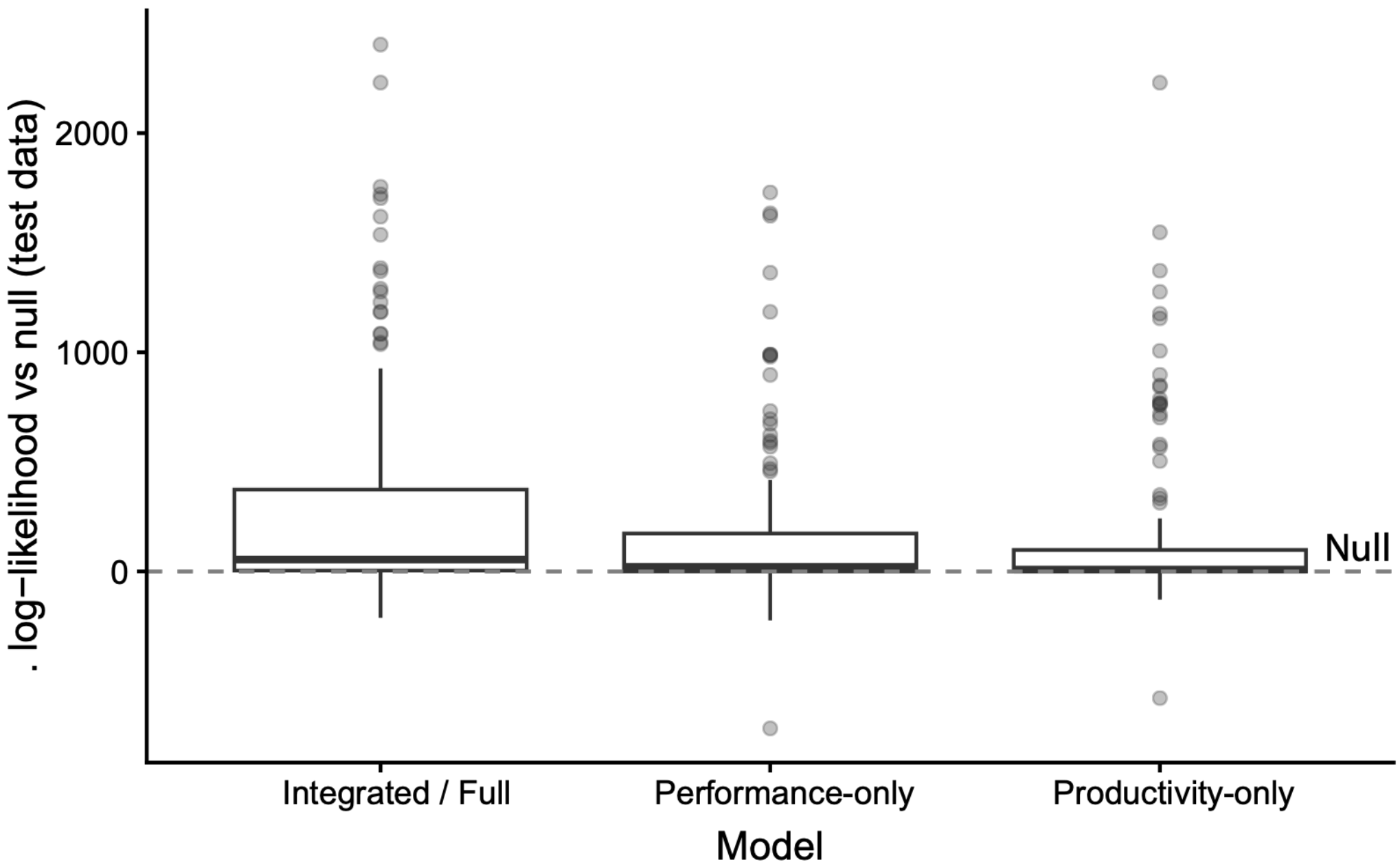

Fig. S4. Study 2 Held-out predictive performance of candidate models. For each participant, models were fit on a randomly selected training set (70% of trials) and evaluated on held-out test trials (30%). Shown are within-participant differences in test log-likelihood relative to the null model (Δ log-likelihood), summarised across participants for each candidate model. Boxplots depict the distribution of Δlog-likelihood values, with positive values indicating better out-of-sample predictive performance than the null model. The dashed horizontal line marks equivalence with the null model (Δ log-likelihood = 0).

Interpretation Note: To evaluate the generalisability of the computational models, we conducted held-out predictive checks in which the four models were fit to a randomly selected subset of 70% trials and evaluated on 30% unseen data (see *Supplementary Methods*). The integrated value-difference model showed the strongest predictive performance, yielding substantially higher held-out log-likelihoods than the null, productivity-only, and performance-only models. At the group level, the integrated model outperformed the null model for 81.4% of participants and showed the largest improvement in predictive accuracy (mean Δlog-likelihood = 314; median = 54.1). Both reduced models also outperformed the null model on average (performance-only: mean ΔLL = 186, 76% > 0; productivity-only: mean ΔLL = 179, 72% > 0). Non-null held-out likelihoods were not estimable for four participants who gave invariant 0.50 responses on all trials. These results demonstrate that combining productivity and performance information improves out-of-sample predictive performance, supporting an account in which decisions of merit reflect integrated value representations rather than reliance on a single dimension.

Supplementary Figure S5. Study 2 K-Means Clustering of Behavioural Allocations

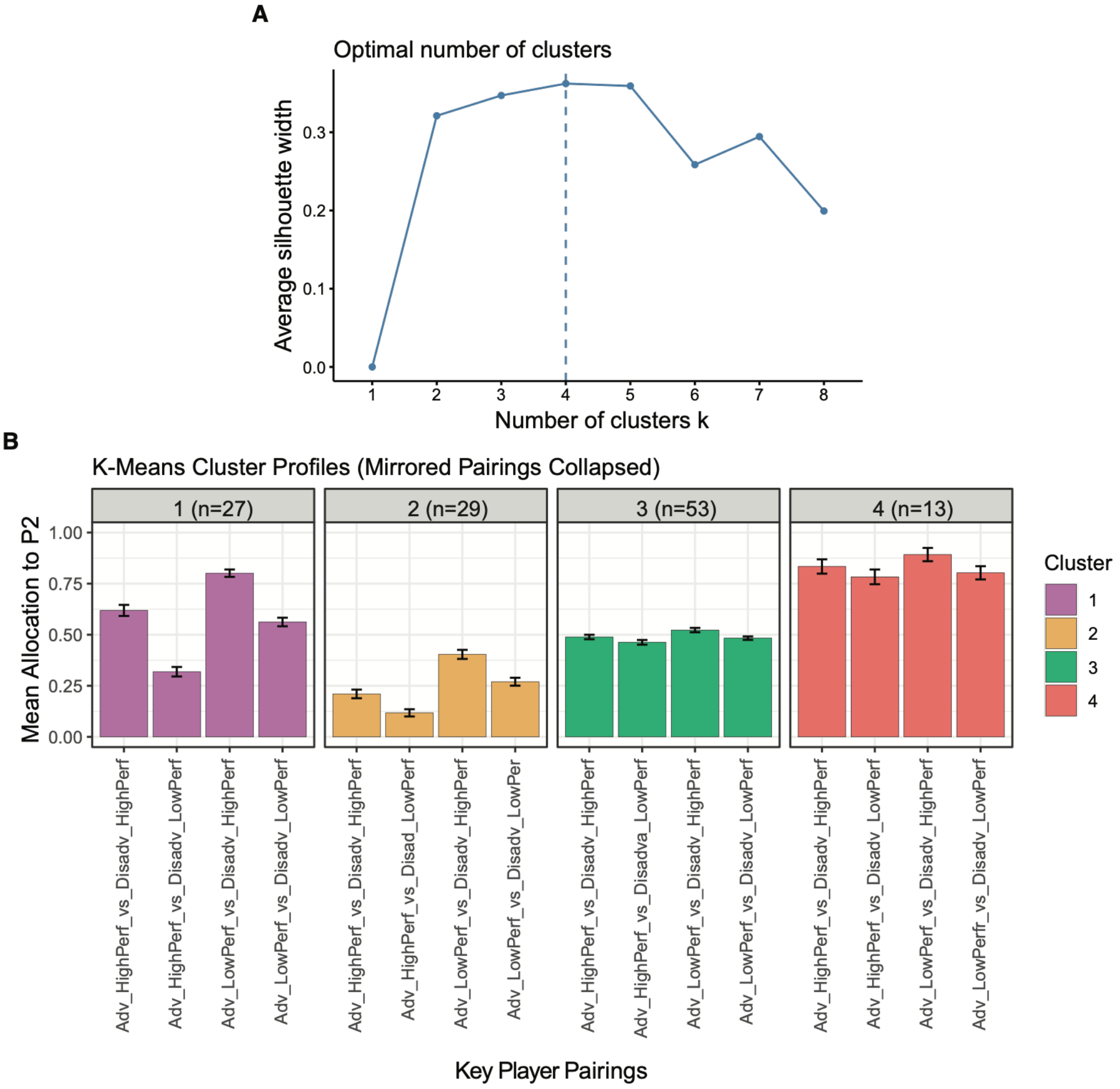

Fig. S5. K-Means Clustering (A) Silhouette Plot. Silhouette analysis of k-means clustering based on participants' mean allocations across player pairings (normalised), showing average silhouette width across candidate cluster solutions; the four-cluster solution was retained. **(B) Mean Allocation to Key Player Pairings by K-Means Clustering Grouping.** Cluster-level allocation profiles display mean allocation to Player 2 (±SE) for key cross-group pairings, based on participant-level mean responses after collapsing mirrored pairings into a single canonical ordering (e.g., Advantaged High Performer vs Disadvantaged Low Performer and its reversed presentation treated as the same pairing). For trials presented in reversed order, allocations were conditionally flipped (1−Response) so that all values reflect allocation relative to the same ordered pairing. Same-group pairings and pairings with identical productivity but differing performance were omitted from plotting solely to help interpret allocation patterns. Patterns converged broadly with allocation patterns generated from thresholding of parameter space: 102 of 122 participants (83.6%) were assigned to corresponding strategy groups. Concordance was high for the Null group (45/47; 95.7%), Legitimising group (27/30; 90.0%), and Performance-dominant group (17/23; 73.9%). Agreement was lower for the Corrective group (13/22; 59.1%), with most of these discrepancies involving participants instead classified as Performance-dominant by the k-means solution.

S8. Study 3 Results

Supplementary Table S8. Study 3 Model comparison

Model comparison (using ΔAIC and ΔBIC relative to Null Model)

Comparison model	**Mean ΔAIC**	**Median ΔAIC**	**Mean ΔBIC**	**Median ΔBIC**
Integrated model	-1224	-225	-1221	-221
Productivity-only model	-930	-59.7	-928	-57.3
Performance-only model	-483	-30.2	-481	-27.8

Note. Δ values are computed as (Model – Null, thus null Mean ΔAIC, Mean ΔBIC = 0). Negative values indicate better fit than the null model. The full model was assigned (k=3) for the AIC/BIC calculations; model comparison results were robust when (k=2) was used instead.

Supplementary Table S9. Study 3 Model Comparison Winning Table

Participant-level model comparison winning table using Bayesian Information Criterion (BIC).

**Model**	**Win Rate***	**Mean ΔBIC**	**Median ΔBIC**
Full model (*prod* + *perf*)	0.712	0.924	0
Null model	0.272	1222	221
Performance only	0.272	741	35.9
Productivity only	0.368	294	6.16

*Win rate reflects the proportion of participants for whom a given model achieved or tied for the lowest BIC (ΔBIC = 0). Mean and median ΔBIC values are computed relative to each participant's best-fitting model; lower values indicate better fit. The full model including both structural productivity and performance sensitivities were most frequently the best fitting model. The full model was assigned (k=3) for the AIC/BIC calculations; model win rates were robust when (k=2) was used instead.

Interpretation Note. Consistent with these findings, likelihood-ratio tests provided evidence in favour of the integrated model. Compared with the null model, the full model yielded a significantly better fit for 72.8% of participants, with 69.6% showing p<.001. Importantly, the full model also outperformed each reduced model for the majority of participants, with significant improvements over the productivity-only model in 56% of participants (48.8% at p<.001) and over the performance-only model in 66.4% of participants (61.6% at p<.001), indicating that both dimensions contributed independently to explaining behaviour. This pattern indicates that most participants simultaneously incorporated productivity and performance when forming their decisions, rather than relying on a single dimension or a simple equality rule.

Supplementary Figure S6. Study 3 Parameter Recovery

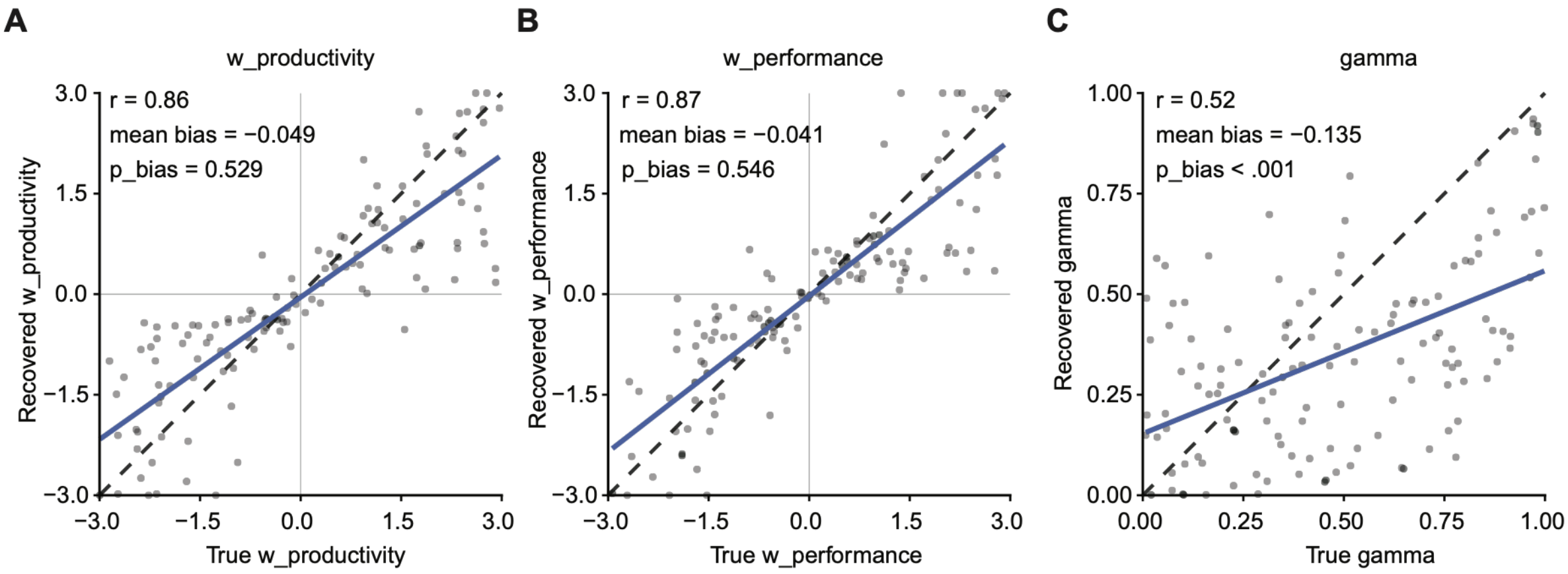

Fig. S6. Study 3 Parameter Recovery. (A) Structural Productivity (*w_productivity)* Recovery. Simulated datasets were generated from known values of raw structural productivity sensitivity, after which the model was refit to recover each parameter. (**B) Performance (*w_performance)* Recovery**. Simulated datasets were generated from known values of raw performance sensitivity, after which the model was refit to recover each parameter. **(C) Senstivity-attentuation (*gamma)* Recovery.** Simulated datasets were generated from known values of sensitivity-attenuation parameter, after which the model was refit to recover each parameter. For A-C, dashed lines indicate perfect recovery (y = x), points represent individual simulations and solid lines indicate the fitted linear recovery relationship. Productivity sensitivity was recovered with higher accuracy (r = 0.86, 95% CI [0.81, 0.90], t(128) = 19, p<0.001), as was performance sensitivity (r = 0.87, 95% CI [0.82, 0.90], t(128) = 19.7, p<0.001), than recovery of the sensitivity-attenuation parameter (r = 0.52, 95% CI [0.38, 0.64], t(128) = 6.92, p<0.001). Bias tests assessed whether recovery error (defined as recovered minus true), differed significantly from zero. These tests showed no evidence of systematic bias for *w_productivity* (mean bias = -0.049, 95% CI [-0.20,0.10], p = 0.529) or *w_performance* (mean bias = -0.041, 95% CI [-0.17, 0.09], p = 0.546), but indicated significant under-recovery of gamma (mean bias = -0.135, 95% CI [-0.18, -0.09], p<0.001). In-panel statistics report the correlation between true and recovered parameters (r), and mean recovery bias with p-value.

Supplementary Figure S7. Study 3 Gamma Simulations and Distribution

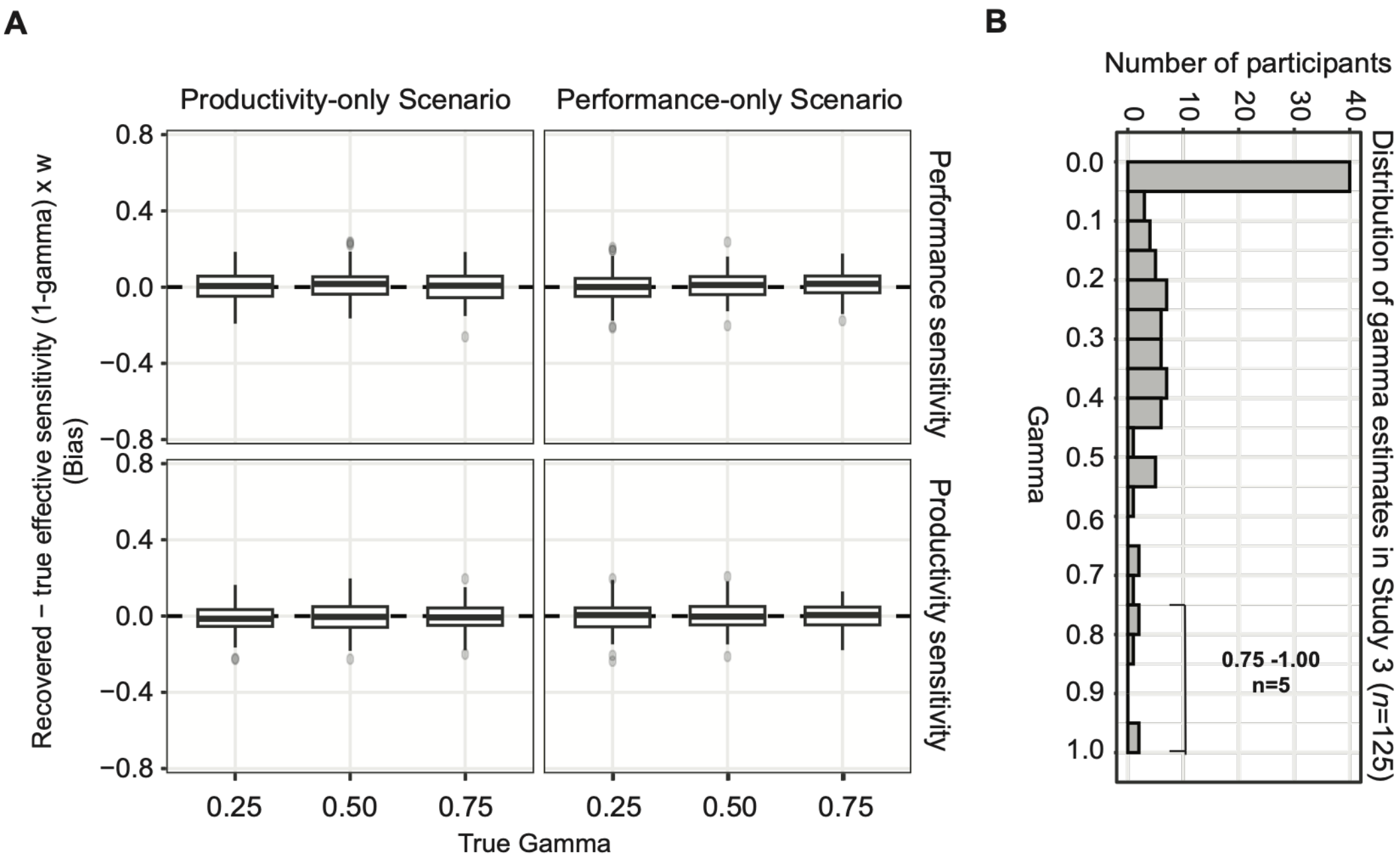

Fig. S7. Study 3 Robustness of Sensitivity Recovery across Gamma Levels. (**A) Gamma Simulations.** Simulations were run to test whether weaker recovery of gamma biased behaviourally expressed sensitivity estimates, defined in line with our model as: (1 - *gamma*) x *w* ('*effective sensitivities*'). Simulated datasets were generated for a productivity-only scenario (i.e. simulated data in which effective productivity sensitivity was fixed at 0.75 and effective performance sensitivity was fixed at 0), and a performance-only scenario (i.e. simulated data in which effective performance sensitivity was fixed at 0.75 and effective productivity sensitivity was fixed at 0) across three true values of gamma (0.25, 0.50, and 0.75). Boxplots show recovery bias, defined as recovered sensitivity minus true sensitivity. The dashed horizontal line indicates zero bias. In the productivity-only scenario, mean bias in effective productivity sensitivity remained close to zero (-0.013, -0.004, and -0.004), as did mean bias in the absent performance sensitivity (0.003, 0.009, and 0.004). Similarly, in the performance-only scenario, mean bias in effective performance sensitivity remained small (-0.005, 0.004, 0.010), as did mean bias in the absent productivity sensitivity (-0.002, 0.002, -0.003). Thus, although gamma itself was underestimated at higher true values, this did not materially bias the behaviourally expressed productivity or performance sensitivity estimates in these single-cue scenarios. (**B) Gamma Distribution.** Distribution of actual gamma estimates in Study 3 (*N*=125), with 5 participants in this sample (4% of total sample) having a gamma higher than 0.75.

Supplementary Figure S8. Study 3 Model Recovery Confusion Matrix

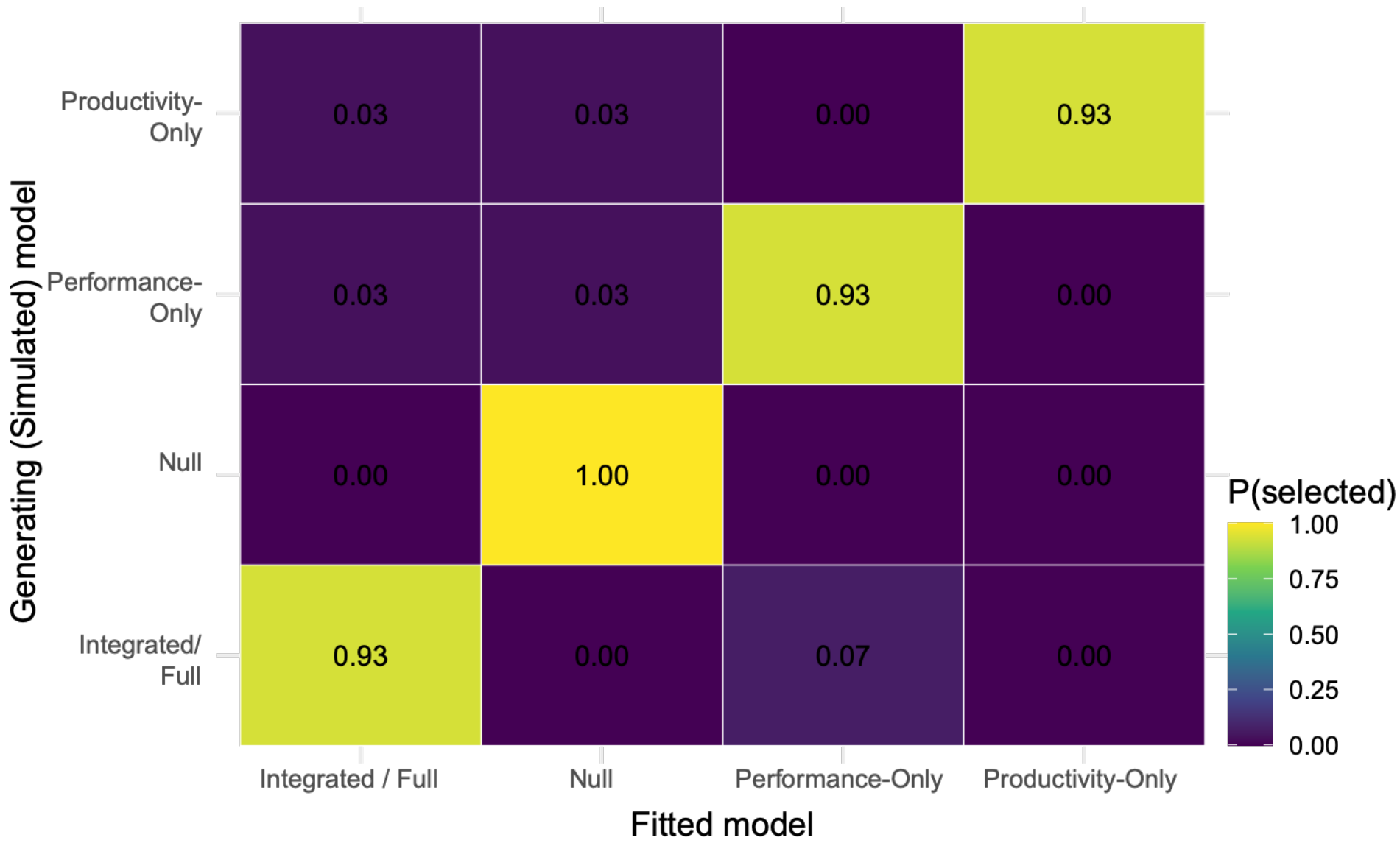

Fig. S8. Study 3 Model recovery confusion matrix. Rows indicate the true data-generating model and columns indicate the model selected as best-fitting based on BIC. Cell values and colour intensity represent the proportion of simulations in which each fitted model was selected for a given generating model. Simulations were conducted using the pooled empirical trial structure (30 simulations per model), with model selection performed after fitting all candidate models to each simulated dataset. High values indicate reliable recovery of the true data-generating model.

Interpretation Note. As an additional conservative check, we repeated model recovery using reduced participant-level trial structures. In this analysis, using 100 simulations per model and sensitivity parameters sampled from [-3, 3], the integrated (full), performance-only, productivity-only, and null models were recovered in 78%, 80%, 82%, and 99% of simulations, respectively. Misclassifications primarily reflected BIC selection of the null model for one-dimensional sensitivity models, rather than confusion between productivity and performance sensitivity.

Supplementary Figure S9: Study 3 Held-out predictive performance of candidate models

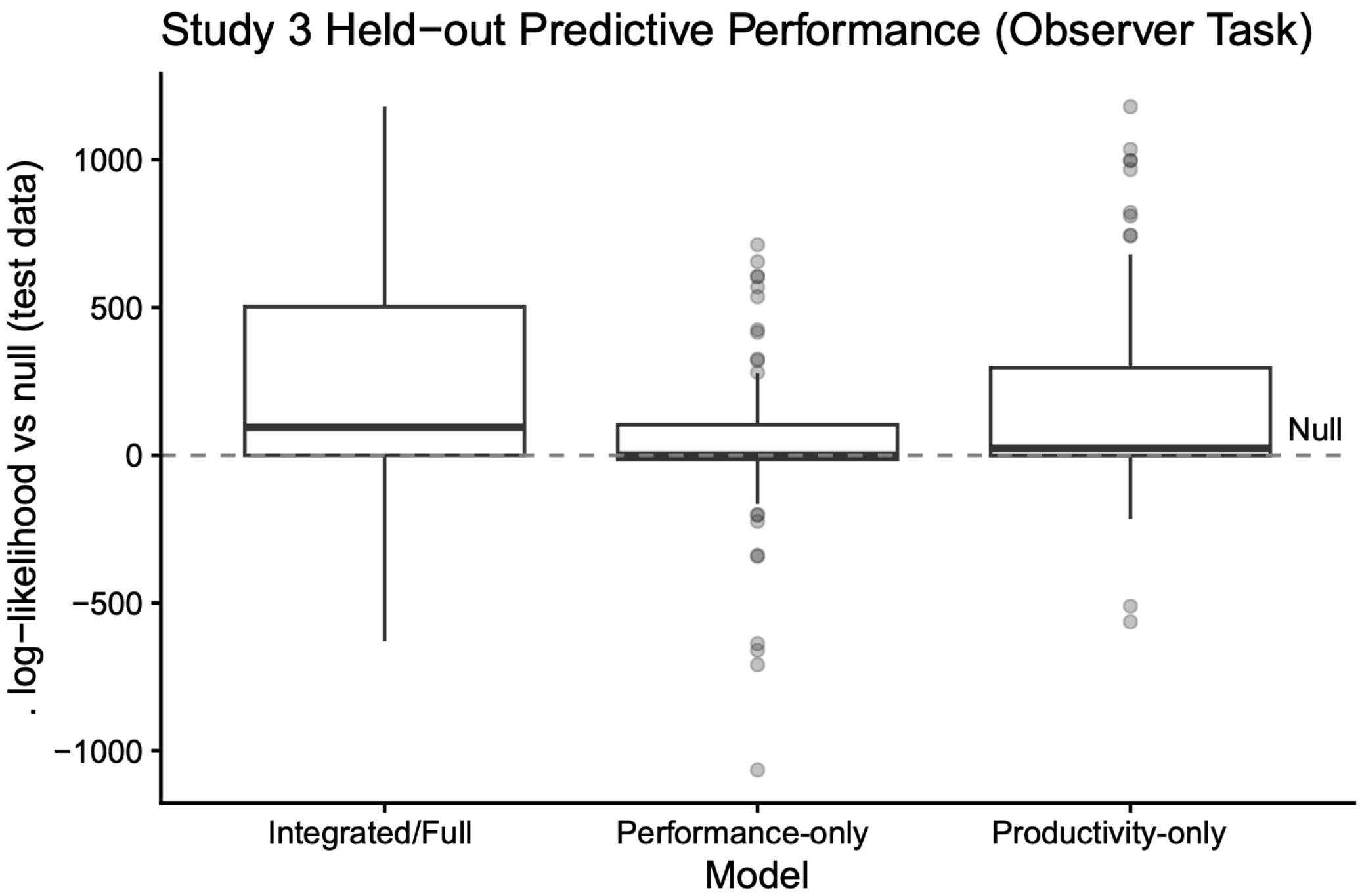

Fig. S9. Study 3 Held-out predictive performance of candidate models. For each participant, models were fit on a randomly selected training set (70% of trials) and evaluated on held-out test trials (30%). Shown are within-participant differences in test log-likelihood relative to the null model (Δ log-likelihood), summarised across participants for each candidate model. Boxplots depict the distribution of Δ log-likelihood values, with positive values indicating better out-of-sample predictive performance than the null model. The dashed horizontal line marks equivalence with the null model (Δ log-likelihood = 0).

S9. Study 4 Results

Supplementary Figure S10. Relative Allocation to In-group versus Out-group

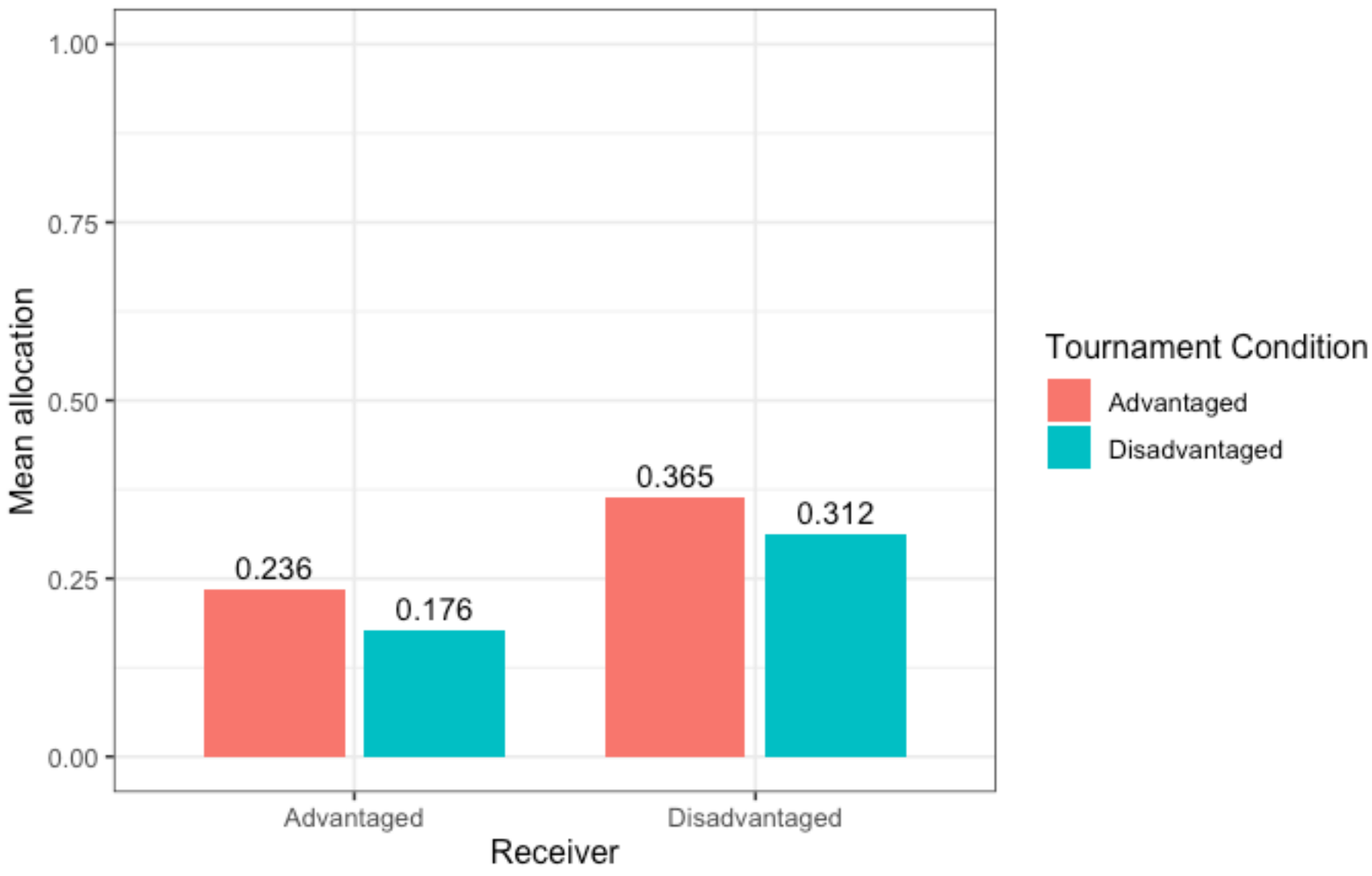

Fig. S10. Mean allocation by participant and receiver tournament condition. Bars show the mean allocation to Advantaged and Disadvantaged receivers, separately for Advantaged and Disadvantaged participants. Advantaged participants allocated on average 0.236 to Advantaged receivers (SD = 0.273, SE = 0.0196, N = 194) and 0.365 to Disadvantaged receivers (SD = 0.299, SE = 0.0214, N = 194), whereas Disadvantaged participants allocated on average 0.176 to Advantaged receivers (SD = 0.254, SE = 0.0182, N = 194) and 0.312 to Disadvantaged receivers (SD = 0.277, SE = 0.0199, N = 194). To formally compare these patterns, we computed a participant-level directional bias score reflecting allocation to the in-group minus allocation to the out-group. Advantaged participants showed a negative directional bias, allocating less to their own (Advantaged) in-group than to the (Disadvantaged) out-group (M = −0.129, SD = 0.257), whereas Disadvantaged participants showed a positive directional bias, allocating more to their own (Disadvantaged) in-group than to the (Advantaged) out-group (M = 0.135, SD = 0.234). These directional bias scores differed significantly between groups, Welch's $t(382.54) = -10.56$, $p < 0.001$, 95% CI [−0.313, −0.214], indicating that structural productivity group was associated with a reversal in the direction of in-/out-group allocation bias.

Supplementary Table S10. Pairwise comparisons for Participant_StructuralProductivity * Participant_Performance interaction

emmeans(lmer_model, pairwise ~ Participant_StructuralProductivity * Participant_Performance, adjust = "bonferroni")
$emmeans

Participant_ StructuralProductivity	Participant_Performance	emmean	SE	asymp.LCL	asymp.UCL
Advantaged	HighPerformer	0.243	0.0236	0.197	0.289
Disadvantaged	HighPerformer	0.181	0.0253	0.131	0.230
Advantaged	LowPerformer	0.366	0.0253	0.317	0.416
Disadvantaged	LowPerformer	0.299	0.0236	0.253	0.345

Results are averaged over the levels of: Receiver_StructuralProductivity, Receiver_Performance
Degrees-of-freedom method: asymptotic
Confidence level used: 0.95

$contrasts

contrast	estimate	SE	z.ratio	p.value
Advantaged HighPerformer - Disadvantaged HighPerformer	0.0624	0.0346	1.804	0.4278
Advantaged HighPerformer - Advantaged LowPerformer	-0.1234	0.0346	-3.567	0.0022
Advantaged HighPerformer - Disadvantaged LowPerformer	-0.0558	0.0333	-1.674	0.5646
Disadvantaged HighPerformer - Advantaged LowPerformer	-0.1857	0.0358	-5.187	<.0001
Disadvantaged HighPerformer - Disadvantaged LowPerformer	-0.1181	0.0346	-3.416	0.0038
Advantaged LowPerformer - Disadvantaged LowPerformer	0.0676	0.0346	1.954	0.3039

Results are averaged over the levels of: Receiver_StructuralProductivity, Receiver_Performance
Degrees-of-freedom method: asymptotic
P value adjustment: bonferroni method for 6 tests

Supplementary Table S11. Pairwise comparisons for Receiver_StructuralProductivity * Receiver_Performance interaction

emmeans(lmer_model, pairwise ~ Receiver_StructuralProductivity * Receiver_Performance, adjust = "bonferroni")

$emmeans

Receiver _StructuralProductivity	Receiver _Performance	emmean	SE	asymp.LCL	asymp.UCL
Advantaged	HighPerformer	0.228	0.0127	0.204	0.253
Disadvantaged	HighPerformer	0.373	0.0127	0.348	0.397
Advantaged	LowPerformer	0.184	0.0127	0.159	0.209
Disadvantaged	LowPerformer	0.304	0.0127	0.279	0.328

Results are averaged over the levels of: Participant_StructuralProductivity, Participant_Performance
Degrees-of-freedom method: asymptotic
Confidence level used: 0.95

$contrasts

contrast	estimate	SE	z.ratio	p.value
Advantaged HighPerformer - Disadvantaged HighPerformer	-0.1442	0.00537	-26.869	<.0001
Advantaged HighPerformer - Advantaged LowPerformer	0.0443	0.00537	8.246	<.0001
Advantaged HighPerformer - Disadvantaged LowPerformer	-0.0752	0.00537	-14.009	<.0001
Disadvantaged HighPerformer - Advantaged LowPerformer	0.1885	0.00537	34.512	<.0001
Disadvantaged HighPerformer - Disadvantaged LowPerformer	0.0690	0.00537	12.860	<.0001
Advantaged LowPerformer - Disadvantaged LowPerformer	-0.1195	0.00537	-22.256	<.0001

Results are averaged over the levels of: Participant_StructuralProductivity, Participant_Performance
Degrees-of-freedom method: asymptotic
P value adjustment: bonferroni method for 6 tests

Supplementary Table S12. Study 4 Model comparison

Model comparison (using ΔAIC and ΔBIC relative to Null Model)

Comparison model	**Mean ΔAIC**	**Median ΔAIC**	**Mean ΔBIC**	**Median ΔBIC**
Integrated model	-1268	-252	-1265	-249
Productivity-only model	-1067	-122	-1066	-120
Performance-only model	-402	-16.8	-400	-14.4

Note. Δ values are computed as (Model – Null, thus null Mean ΔAIC, Mean ΔBIC = 0). Negative values indicate superior fit than the null model. The full model was assigned (k=3) for the AIC/BIC calculations; model comparison results were robust when (k=2) was used instead.

Supplementary Table S13. Study 4 Model Comparison Winning Table

Participant-level model comparison winning table using Bayesian Information Criterion (BIC).

**Model**	**Win Rate***	**Mean ΔBIC**	**Median ΔBIC**
Full model (prod + perf)	0.716	1.02	0
Null model	0.332	1266	249
Performance only	0.286	866	68.2
Productivity only	0.446	200	4.20

*Win rate reflects the proportion of participants for whom a given model achieved or tied for the lowest BIC (ΔBIC = 0). Mean and median ΔBIC values are computed relative to each participant's best-fitting model; lower values indicate better fit. The full model including both structural productivity and performance sensitivities was most frequently the best-fitting model. The full model was assigned (k=3) for the AIC/BIC calculations; model win rates were robust when (k=2) was used instead.

Interpretation Note. Consistent with these findings, likelihood-ratio tests provided support in favour of the integrated model. Compared with the null model, the full model yielded a significantly better fit for 66.5% of participants (with 65.7% showing p<.001). Importantly, the full model also outperformed each reduced model for most participants, with significant improvements over the productivity-only model in 48.5% of participants (42.3% at p<.001) and over the performance-only model in 64.2% of participants (61.6% at p<.001). Consistent with these findings, participant-level BIC model selection showed that the integrated model was the best-fitting model for 71.6% of participants. In contrast, the null model, productivity-only model and performance-only model accounted for behaviour in smaller subsets of participants (null: 33.2%, productivity-only: 44.6%, performance-only: 28.6%). The full model was assigned (k=3) for the AIC/BIC calculations; model win rates were robust when (k=2) was used instead.

Supplementary Figure S11. Study 4 Parameter Recovery

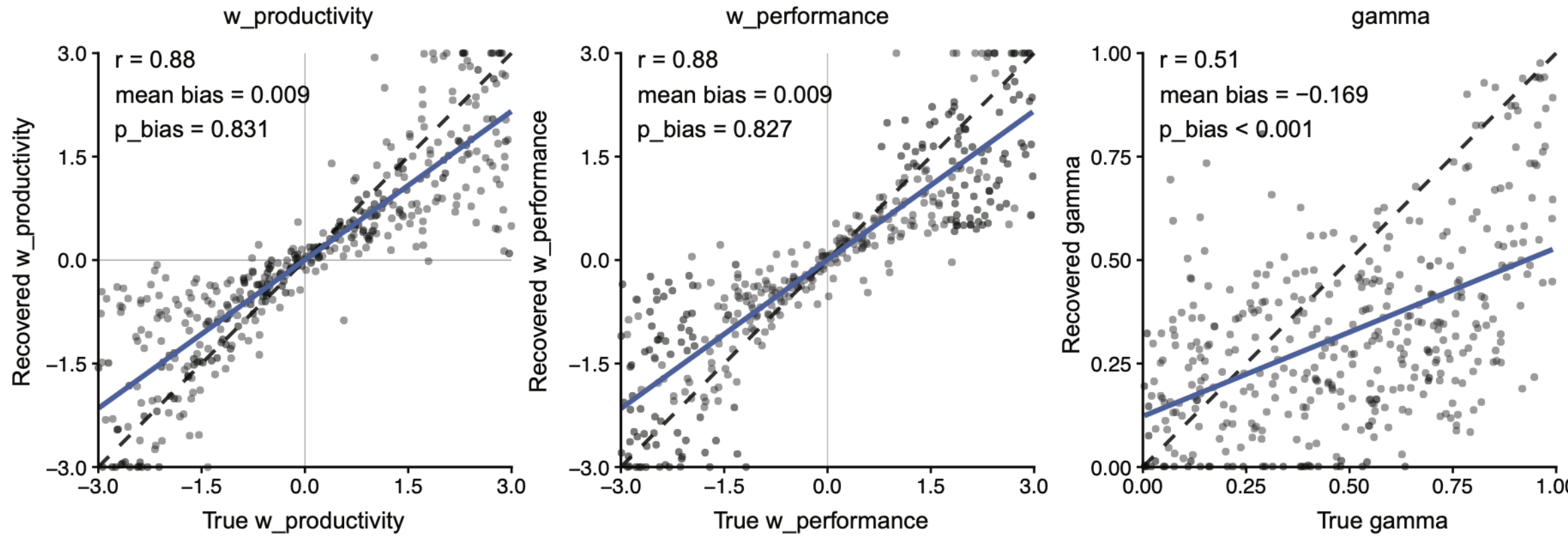

Fig. S11. Study 4 Parameter Recovery. (A) Structural Productivity (*w_productivity)* Recovery. Simulated datasets were generated from known values of raw structural productivity sensitivity, after which the model was refit to recover each parameter. **(B) Performance (*w_performance)* Recovery**. Simulated datasets were generated from known values of raw performance sensitivity, after which the model was refit to recover each parameter. **(C) Senstivity-attentuation (*gamma)* Recovery.** Simulated datasets were generated from known values of sensitivity-attenuation parameter, after which the model was refit to recover each parameter. For A-C, dashed lines indicate perfect recovery (y = x), points represent individual simulations and solid lines indicate the fitted linear recovery relationship. Productivity sensitivity was recovered with higher accuracy (r = 0.88, 95% CI [0.85, 0.90], t(398) = 36.4, p<0.001), as was performance sensitivity (r = 0.88, 95% CI [0.85, 0.89], t(398) = 36.6, p<0.001), than recovery of the sensitivity-attenuation parameter (r = 0.51, 95% CI [0.44, 0.58], t(398) = 11.9, p<0.001). Bias tests assessed whether recovery error (defined as recovered minus true), differed significantly from zero. These tests showed no evidence of systematic bias for *w_productivity* (mean bias = 0.009, 95% CI [-0.07,0.09], p = 0.831) or *w_performance* (mean bias = 0.009, 95% CI [-0.07, 0.09], p = 0.827), but indicated significant under-recovery of gamma (mean bias = -0.169, 95% CI [-0.19, -0.14], p<0.001). In-panel statistics report the correlation between true and recovered parameters (r), and mean recovery bias with p-value.

Supplementary Figure S12. Study 4 Gamma Simulations and Distribution

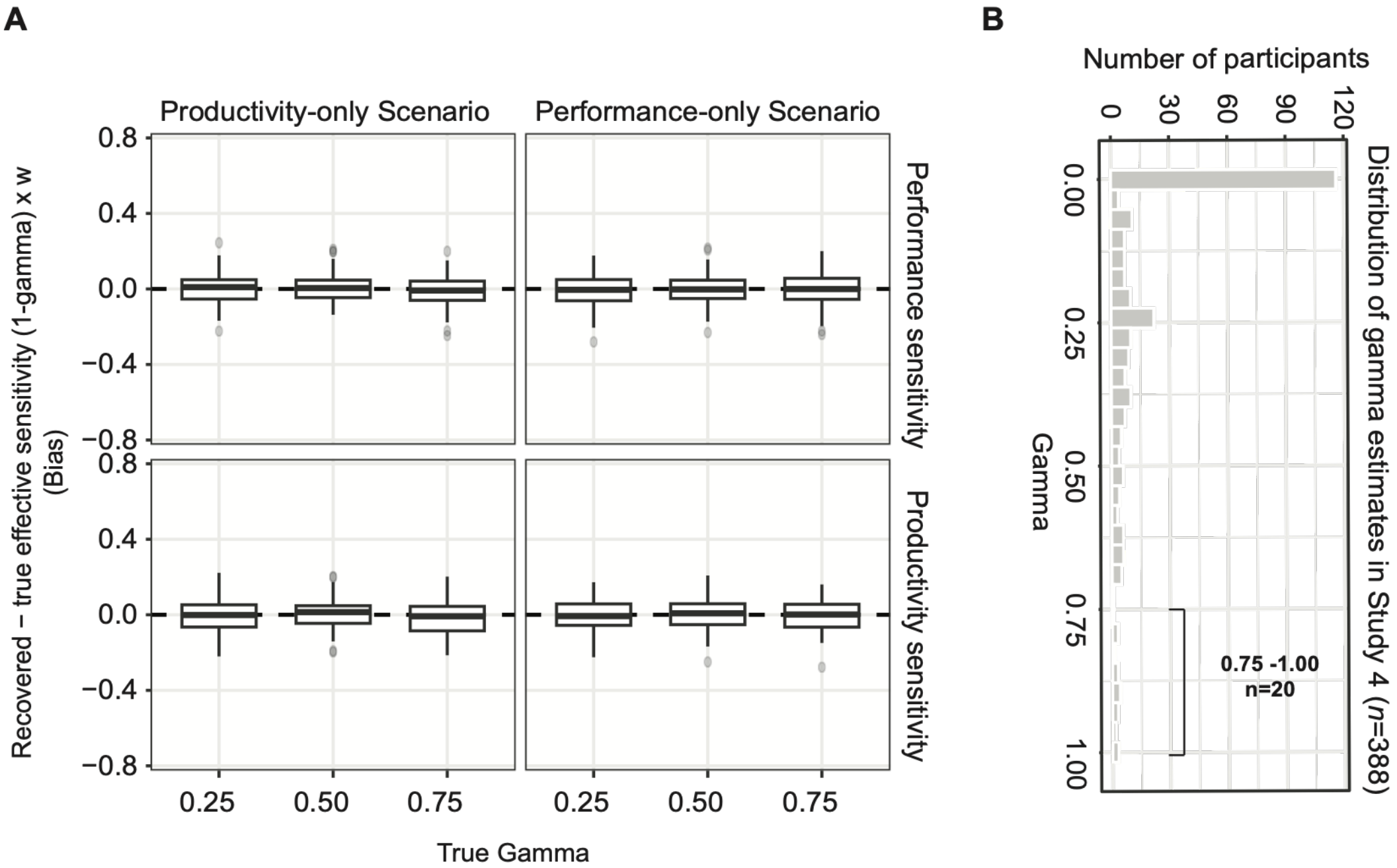

Fig. S12. Study 4 Robustness of sensitivity recovery across gamma levels. (A) Gamma Simulations. Simulations were run to test whether weaker recovery of gamma biased the behaviourally expressed sensitivity estimates, defined in line with our model as: (1 - *gamma*) x *w* (‘*effective sensitivities*’). Simulated datasets were generated for a productivity-only scenario (i.e. simulated data in which effective productivity sensitivity was fixed at 0.75 and effective performance sensitivity was fixed at 0), and a performance-only scenario (i.e. simulated data in which performance sensitivity was fixed at 0.75 and productivity sensitivity was fixed at 0) across three true values of gamma (0.25, 0.50, and 0.75). Boxplots show recovery bias, defined as recovered minus true sensitivity. The dashed horizontal line indicates zero bias. In the productivity-only scenario, mean bias in effective productivity sensitivity remained close to zero (-0.002, -0.010 and 0.001), as did mean bias in the absent performance sensitivity (0.005, 0.006 and 0.010). Similarly, in the performance-only scenario, mean bias in effective performance sensitivity remained small (0.012, -0.010 and 0.0003), as did mean bias in the absent productivity sensitivity (0.003, 0.005 and 0.007). Thus, although gamma itself was underestimated at higher true values, this did not materially bias the behaviourally expressed productivity or performance sensitivity estimates in these single-cue scenarios. **(B) Gamma Distribution.** Distribution of actual gamma estimates in Study 4 (*N*=388), with 20 participants in this sample (5.15% of total sample) having a gamma higher than 0.75.

Supplementary Figure S13. Study 4 Model Recovery Confusion Matrix

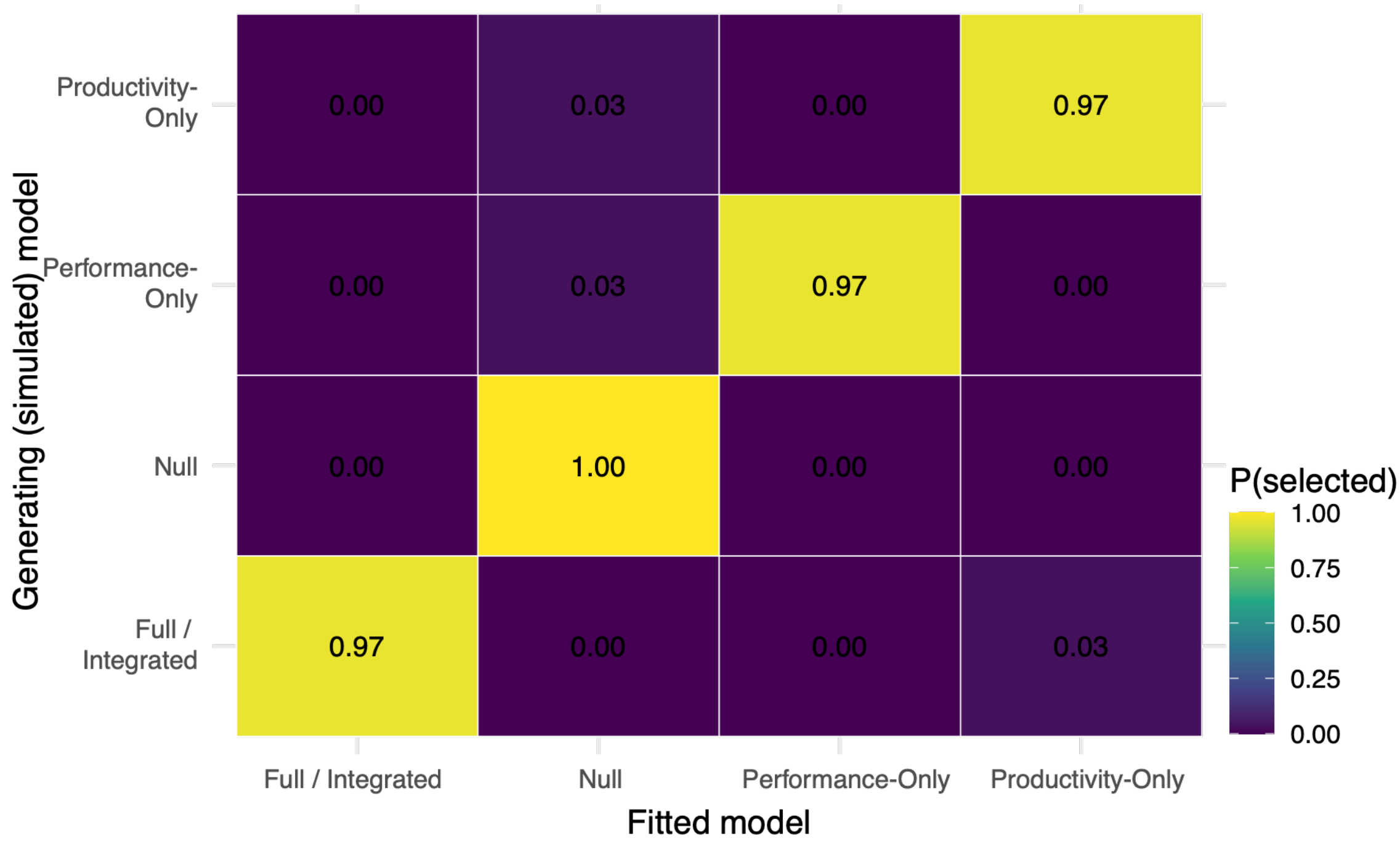

Fig. S13. Study 4 Model recovery confusion matrix. Rows indicate the true data-generating model and columns indicate the model selected as best-fitting based on BIC. Cell values and colour intensity represent the proportion of simulations in which each fitted model was selected for a given generating model. Simulations were conducted using the empirical trial structure (30 simulations per model), with model selection performed after fitting all candidate models to each simulated dataset. High values indicate reliable recovery of the true data-generating model.

Interpretation Note. As an additional conservative check, we repeated model recovery using reduced participant-level trial structures. In this analysis, using 100 simulations per model and sensitivity parameters sampled from [-3, 3], the integrated (full), performance-only, productivity-only, and null models were recovered in 80%, 73%, 80%, and 98% of simulations, respectively. Misclassifications primarily reflected BIC selection of the null model for one-dimensional sensitivity models, rather than confusion between productivity and performance sensitivity.

Supplementary Figure S14: Study 4 Held-out predictive performance of candidate models

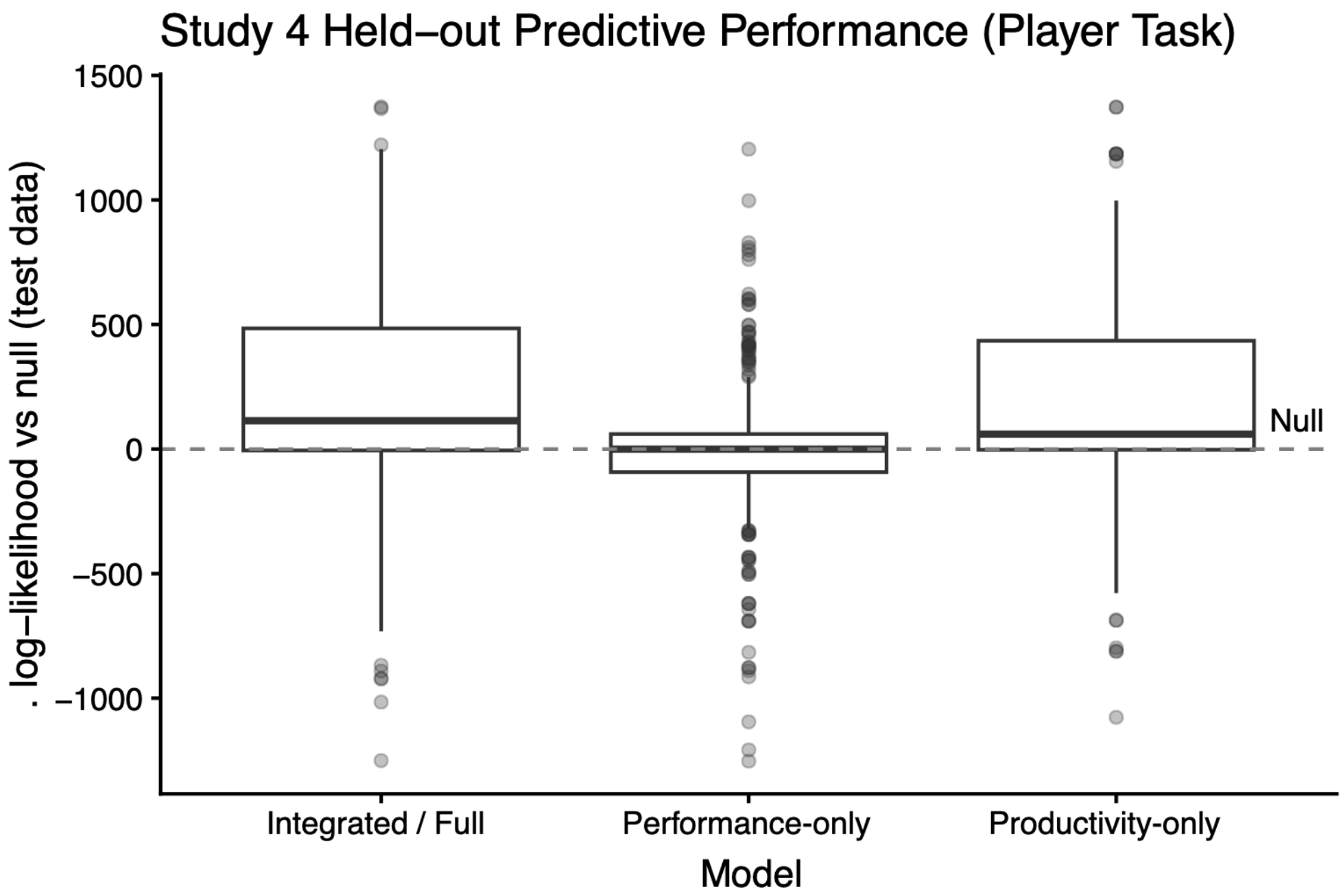

Fig. S14. Study 4 Held-out predictive performance of candidate models. For each participant, models were fit on a randomly selected training set (70% of trials) and evaluated on held-out test trials (30%). Shown are within-participant differences in test log-likelihood relative to the null model (Δ log-likelihood), summarised across participants for each candidate model. Boxplots depict the distribution of Δ log-likelihood values, with positive values indicating better out-of-sample predictive performance than the null model. The dashed horizontal line marks equivalence with the null model (Δ log-likelihood = 0).

S10. Regime Disaggregation Exploratory Results

Supplementary Figure S15. Exploratory description of merit regimes in Study 2 (*N*=122)

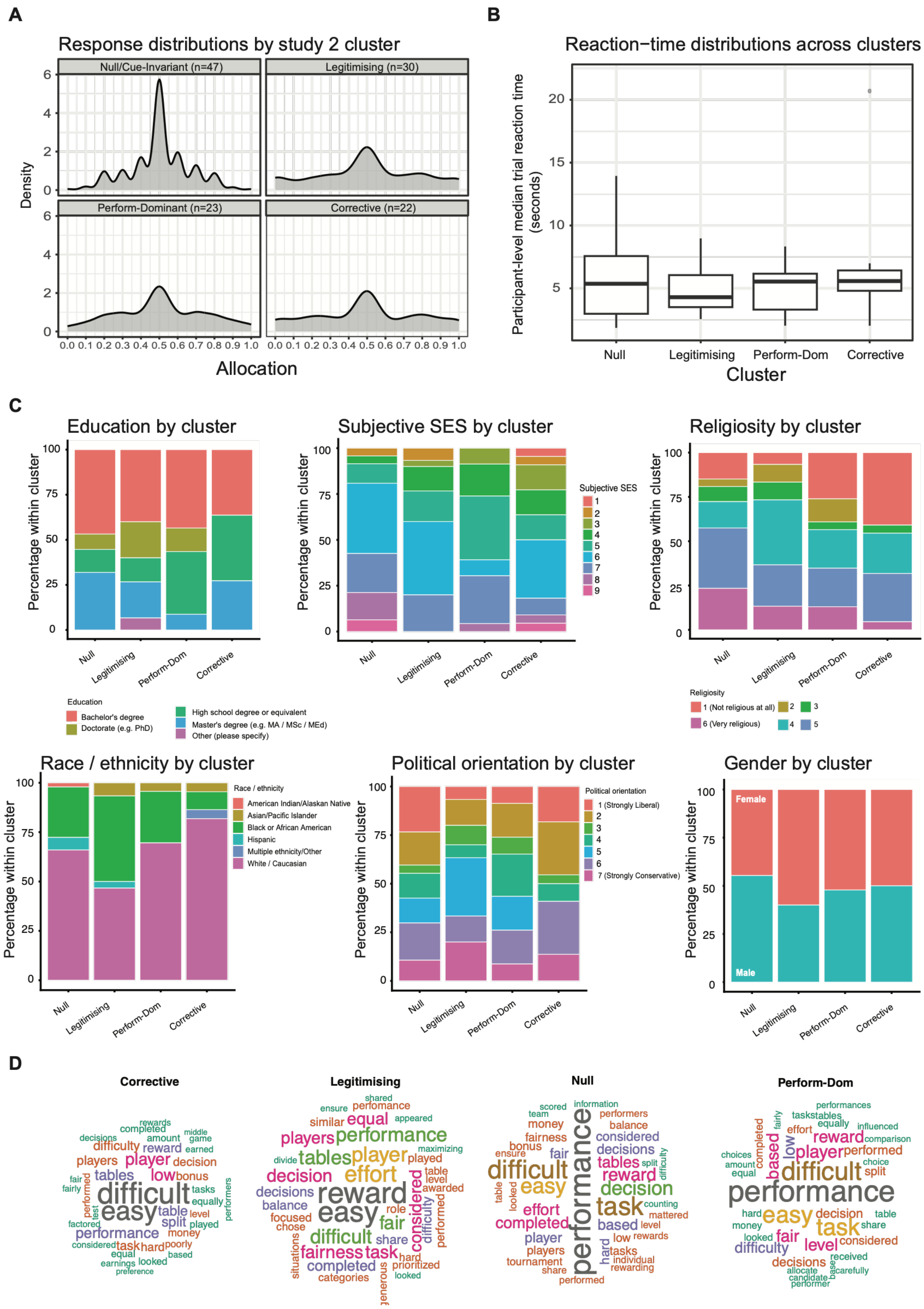

Fig. S15. Exploratory description of behavioural, demographic, and self-reported characteristics of participant decision-making clusters in Study 2. (A) Response Distribution by Cluster. Density distributions of allocation decisions across all trials for

participants assigned to the Null, Corrective, Legitimising, and Performance-Dominant clusters. (**B) Reaction Time by Cluster.** Participant-level median reaction times across clusters. Boxes indicate the interquartile range, horizontal lines indicate the median, whiskers indicate 1.5 × IQR. (**C) Demographic Disaggregation by Cluster.** Demographic composition of each cluster, showing the proportional distribution of education, subjective SES, religiosity, race, political orientation, and gender. Percentages are calculated within cluster. (**D) Open Strategy Responses by Cluster.** Word clouds derived from participants' open-ended strategy descriptions after removal of common task-related terms. Word size is proportional to frequency within each cluster and is shown for descriptive purposes only. Together, these analyses provide a descriptive characterisation of cluster membership beyond the computational model classifications.

Supplementary Figure S16. Exploratory description of merit regimes in Study 3 (*N*=125)

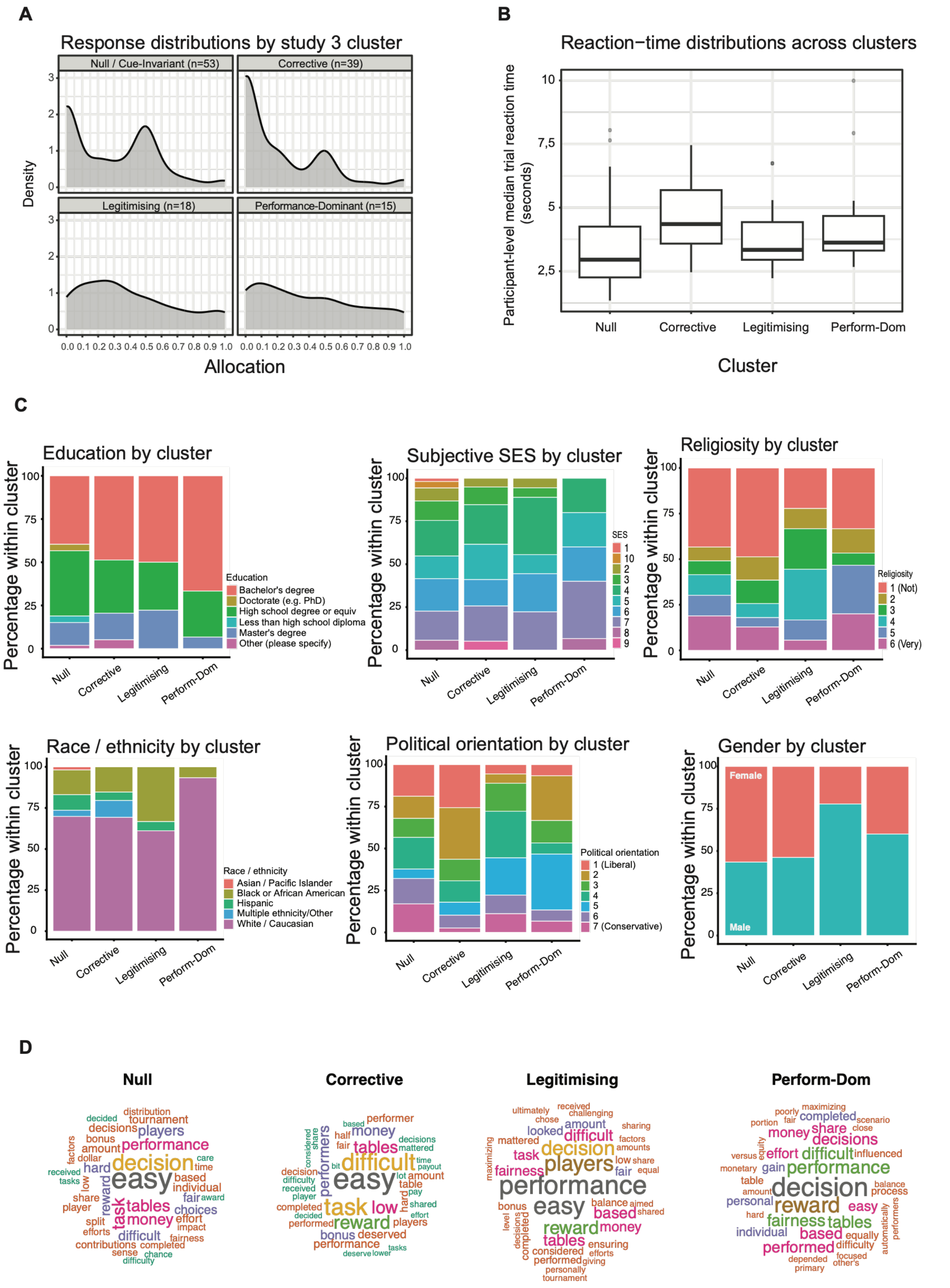

Fig. S16. Exploratory description of behavioural, demographic, and self-reported characteristics of participant decision-making clusters in Study 3. (A) Response Distribution by Cluster. Density distributions of allocation decisions across all trials for participants assigned to the Null, Corrective, Legitimising, and Performance-Dominant

clusters. (**B) Reaction Time by Cluster.** Participant-level median reaction times across clusters. Boxes indicate the interquartile range, horizontal lines indicate the median, whiskers indicate 1.5 × IQR. (**C) Demographic Disaggregation by Cluster.** Demographic composition of each cluster, showing the proportional distribution of education, subjective SES, religiosity, race, political orientation, and gender. Percentages are calculated within cluster. (**D) Open Strategy Responses by Cluster.** Word clouds derived from participants' open-ended strategy descriptions after removal of common task-related terms. Word size is proportional to frequency within each cluster and is shown for descriptive purposes only. Together, these analyses provide a descriptive characterisation of cluster membership beyond the computational model classifications., race/ethnicity, and religiosity. Percentages are calculated within cluster.

Supplementary Figure S17. Exploratory description of merit regimes in Study 4 (*N*=388)

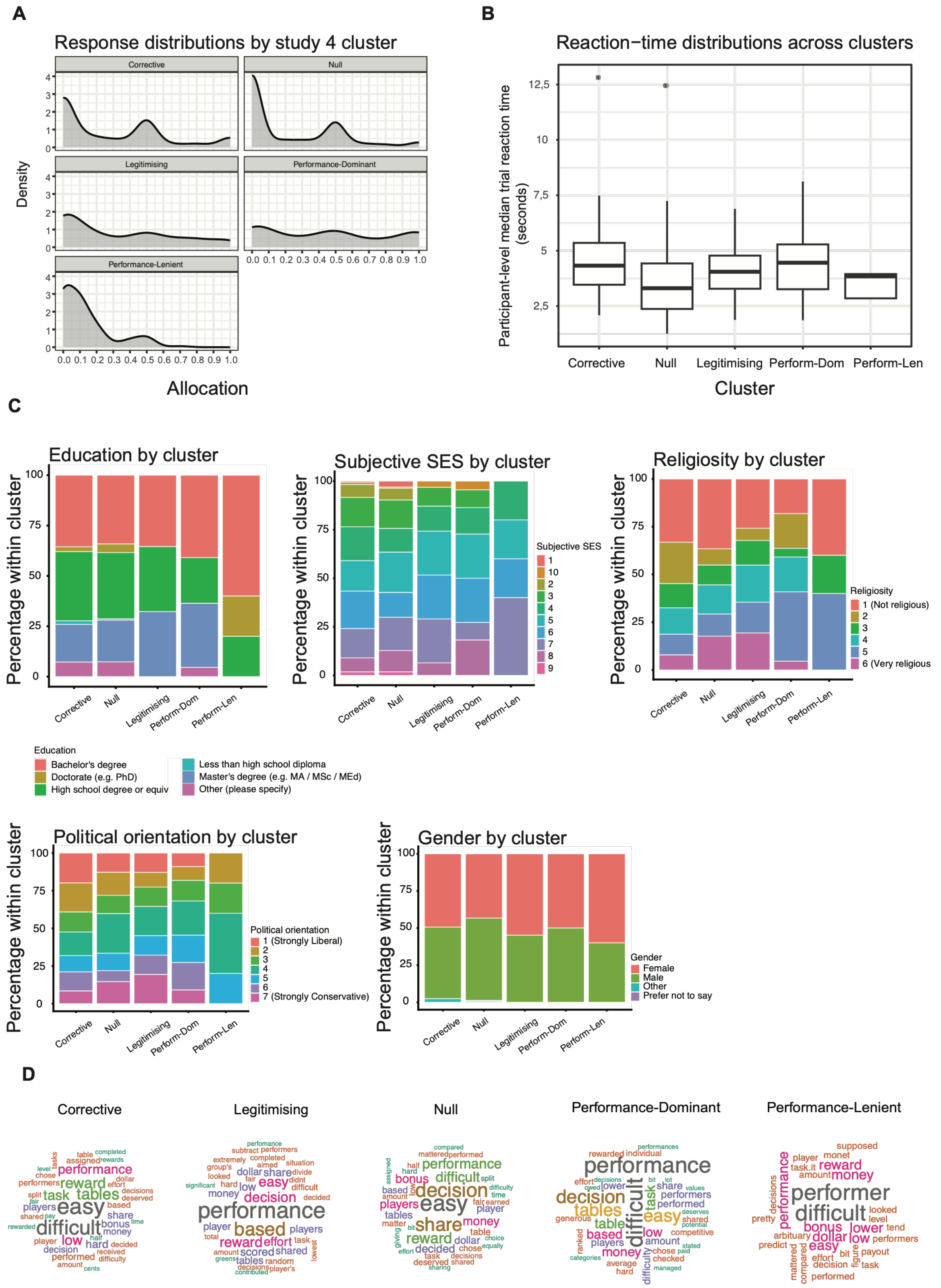

Fig. S17. Exploratory description of behavioural, demographic, and self-reported characteristics of participant decision-making clusters in Study 4. (A) Response Distribution by Cluster. Density distributions of allocation decisions across all trials for

participants assigned to the Null, Corrective, Legitimising, Performance-Dominant and Performance-Lenient clusters. (**B) Reaction Time by Cluster.** Participant-level median reaction times across clusters. Boxes indicate the interquartile range, horizontal lines indicate the median, whiskers indicate 1.5 × IQR. **(C) Demographic Disaggregation by Cluster.** Demographic composition of each cluster, showing the proportional distribution of education, subjective SES, religiosity, political orientation, and gender. Percentages are calculated within cluster. (**D) Open Strategy Responses by Cluster.** Word clouds derived from participants' open-ended strategy descriptions after removal of common task-related terms. Word size is proportional to frequency within each cluster and is shown for descriptive purposes only. Together, these analyses provide a descriptive characterisation of cluster membership beyond the computational model classifications.

S11. Regime by Tournament Conditions

Supplementary Figure S18. Cluster Allocations across Tournament Condition

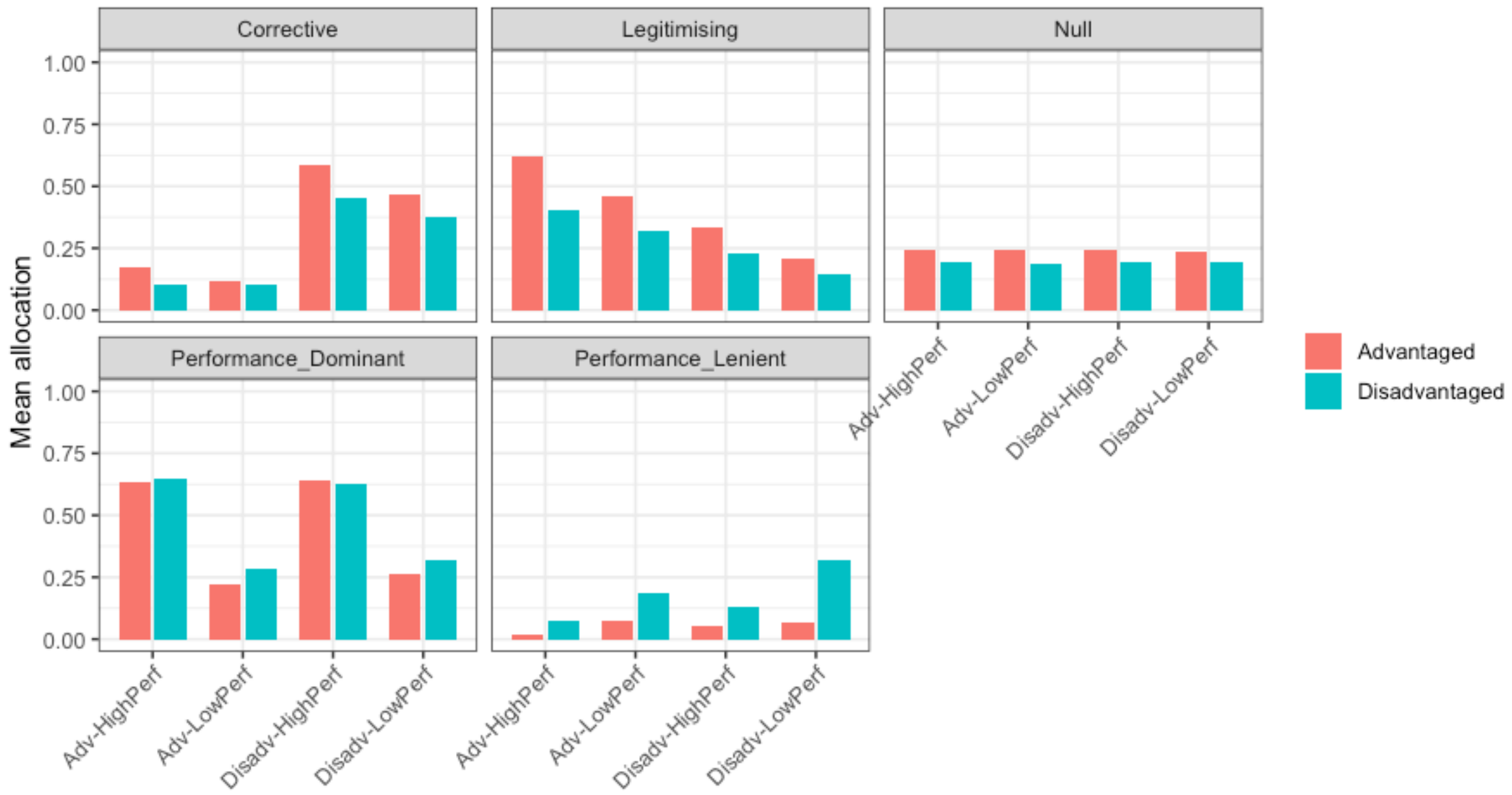

Fig. S18. Cluster Allocations across Tournament Conditions by Cluster. Mean allocation to receiver as a function of participant tournament conditions and merit regime cluster. Bars show the mean participant-level allocation to each receiver category, separately for Advantaged and Disadvantaged participants, with facets per clusters. Cluster sizes by participant condition were: Corrective, Advantaged N=74 and Disadvantaged N=92; Legitimising, Advantaged N=13 and Disadvantaged N=18; Null, Advantaged N=92 and Disadvantaged N=72; Performance-Dominant, Advantaged N=13 and Disadvantaged N=9; and Performance-Lenient, Advantaged N=2 and Disadvantaged N=3.

S12. Socio-Demographic Exploratory Results

(i) Demographic Analyses and Third-Party Judgement: Study 2 (*N*=122)

In Study 2, we conducted exploratory socio-demographic analyses to examine whether individual differences in model parameters were associated with age, subjective socioeconomic status, political orientation, religion, gender, or education.

We first fit a multivariate linear model with effective structural productivity sensitivity and performance sensitivity (1 - *gamma*) x *w* ('*effective sensitivities*') as joint outcomes. Using Pillai's trace, age and SES showed associations with the joint parameter profile, age: Pillai = 0.055, $F(2,111) = 3.20$, $p = 0.044$; SES: Pillai = .059, $F(2,111) = 3.49$, $p = 0.034$. However, neither association survived Holm correction across the demographic predictors, age: *p_Holm* = 0.222; SES: *p_Holm* = 0.204. Political orientation was not associated with the joint parameter profile, Pillai = .026, $F(2,111) = 1.46$, p = .236, *p_Holm* = 0.429 (See **Supplementary Table S14**).

We then examined parameter-specific predictor-level tests. For effective structural productivity sensitivity, age showed an association after correction, $F(1,112) = 9.80$, $p = 0.002$, *p_Holm* = .013, partial $\eta^2 = 0.081$. Older participants showed stronger effective sensitivity to structural productivity. Education was also associated with structural productivity sensitivity before correction, $F(4,112) = 3.21$, $p = .016$, partial $\eta^2 = .103$, but this effect did not survive Holm correction, p_Holm = 0.077. For performance sensitivity, SES and gender showed associations, SES: $F(1,112) = 5.86$, $p = 0.017$; gender: $F(1,112) = 4.15$, $p = 0.044$, but neither survived Holm correction. No other demographic predictor explained effective performance sensitivity. See **Supplementary Table S15**.

Finally, coefficient-level follow-up tests were treated as descriptive, and the only coefficient-level association that survived Holm correction within outcome was age predicting effective structural productivity sensitivity, b = 0.033, SE = 0.010, t = 3.13, $p = .002$, *p_Holm* = 0.020. Political orientation did not predict effective structural productivity sensitivity, b = −0.018, $p = 0.777$, or effective performance sensitivity, b = 0.064, $p = .211$. See **Supplementary Table S16**.

Together, these exploratory analyses suggest that age is the most consistent demographic correlate of structural productivity sensitivity, whereas political orientation does not explain individual differences in either sensitivity.

Supplementary Table S14. Study 2 (*N*=122) Multivariate regression of socio-demographics on jointly estimated effective parameters

	Df	Pillai	F (df1, df2)	P-value (Uncorrected)	P (Holm)
**age**	1	0.055	3.200 (2, 111)	0.044 *	0.222
**SES**	1	0.059	3.487 (2, 111)	0.034 *	0.204
**political orientation**	1	0.026	1.465 (2, 111)	0.236	0.429
**religion**	1	0.028	1.577 (2, 111)	0.211	0.429
**gender**	1	0.039	2.277 (2, 111)	0.107	0.429
**education**	4	0.108	1.598 (8, 224)	0.126	0.429

Significance codes: 0 '***', 0.001 '**', 0.01 '*', 0.05 '.' 0.1 ' ' 1

Supplementary Table S15. Study 2 (N=122) Exploratory univariate analyses examining demographic predictor of structural productivity and performance sensitivities, respectively

**Outcome**	**Predictor**	df	Partial η²	F	P-value (uncorrected)	p (Holm)
**Effective productivity sensitivity**	age	1	0.081	9.80	0.002 **	0.01 *
	SES	1	0.004	0.406	0.524	1.000
	political orientation	1	0.001	0.081	0.777	1.00
	religion	1	0.007	0.840	0.361	1.000
	gender	1	0.103	0.813	0.369	1.000
	education	4	0.026	3.21	0.02 *	0.077
**Effective performance sensitivity**	age	1	0.001	0.166	0.685	1.000
	SES	1	0.050	5.86	0.02 *	0.103
	political orientation	1	0.013	1.58	0.211	0.845
	religion	1	0.002	0.179	0.673	1.000
	gender	1	0.036	4.15	0.04 *	0.220
	education	4	0.004	0.107	0.980	1.000

Note. Holm correction was applied separately within each outcome. Partial η² values are reported as measures of effect size.

Supplementary Table S16. Study 2 (N=122) Coefficient-level regression estimates for demographic predictors of sensitivity parameters

Outcome	Term	Estimate	Std_error	T_value	p_value (uncorrected)	p_holm
**Effective productivity sensitivity**	age	0.033	0.010	3.1308	0.002 **	0.02 *
	SES	0.057	0.090	0.639	0.524	1
	political_orientation	-0.018	0.064	-0.284	0.777	1
	religion	-0.077	0.084	-0.916	0.361	1
	genderMale	0.236	0.261	0.902	0.369	1
	educationDoctorate (e.g. PhD)	-0.745	0.425	-1.752	0.082	0.577
	educationHigh school degree or equivalent	0.599	0.352	1.699	0.092	0.577
	educationMaster's degree (e.g. MA / MSc / MEd)	0.143	0.314	0.454	0.651	1
	educationOther (please specify)	-2.166	0.965	-2.244	0.027 *	0.214
**Effective performance sensitivity**	age	-0.003	0.008	-0.407	0.685	1
	SES	-0.173	0.072	-2.420	0.017 *	0.154
	political_orientation	0.064	0.051	1.257	0.211	1
	religion	-0.028	0.067	-0.423	0.673	1
	genderMale	-0.425	0.208	-2.037	0.043 *	0.352
	educationDoctorate (e.g. PhD)	-0.116	0.339	-0.343	0.732	1
	educationHigh school degree or equivalent	-0.087	0.281	-0.311	0.757	1
	educationMaster's degree (e.g. MA / MSc / MEd)	-0.031	0.251	-0.123	0.903	1
	educationOther (please specify)	0.334	0.770	0.434	0.665	1

(ii) Demographic Analyses and Observer Allocation: Study 3 (n = 125)

In Study 3, we conducted exploratory socio-demographic analyses to examine whether individual differences in model parameters were associated with age, subjective socioeconomic status (SES), political orientation, religion, gender, or education.

We first fit a multivariate linear model with effective structural productivity sensitivity and performance sensitivity (1 - *gamma*) x *w* (‘*effective sensitivities*’) as joint outcomes. Using Pillai’s trace, political orientation was associated with the joint parameter profile, Pillai = .111, $F(2,113) = 7.03$, $p = 0.001$, and this association survived multiple-comparison correction, $p_{Holm} = .008$. No other demographic predictor was reliably associated with the joint parameter profile, all Holm-corrected *ps* = 1.000. See **Supplementary Table S17**.

We then examined parameter-specific predictor-level tests through follow-up univariate analyses. For structural productivity sensitivity, political orientation showed a statistically significant association, $F(1,114) = 8.42$, $p = 0.004$, $p_{Holm} = .027$, partial $\eta^2 = .069$. Higher political-orientation scores were associated with lower sensitivity to structural productivity. No other demographic predictor was associated with structural productivity sensitivity after correction. For performance sensitivity, no demographic predictor showed a reliable association, all Holm-corrected *ps* = 1.000. See **Supplementary Table S18**.

Finally, coefficient-level follow-up tests were treated as descriptive and showed that political orientation negatively predicted structural productivity sensitivity, $b = -0.217$, $SE = 0.075$, $t = -2.90$, $p = .004$, $p_{Holm} = .045$ within outcome. In contrast, political orientation did not predict effective performance sensitivity, $b = 0.039$, $SE = 0.050$, $t = 0.79$, $p = 0.434$. See **Supplementary Table S19**.

Collectively, these exploratory analyses suggest that political orientation is the most consistent demographic correlate of structural productivity sensitivity in Study 3, whereas age, SES, religion, gender, and education did not reliably explain individual differences in either sensitivity.

Supplementary Table S17. Study 3 (*N*=125) Exploratory MANOVA examining socio-demographics on jointly estimated parameters

Predictor	Pillai	F (df1, df2)	P-value (Uncorrected)	P (Holm)
**age**	0.001	0.05 (2, 113)	0.955	1.000
**SES**	0.027	1.56 (2, 113)	0.214	1.000
**political orientation**	0.111	7.03 (2, 113)	0.001 **	0.008
**religion**	0.008	0.43 (2, 113)	0.650	1.000
**gender**	0.019	1.10 (2, 113)	0.335	1.000
**education**	0.049	0.58 (10, 228)	0.831	1.000

Significance codes: 0 '***' 0.001 '**' 0.01 '*' 0.05 '.' 0.1 ' ' 1

Supplementary Table S18. Study 3 (*N*=125) Exploratory univariate analyses examining demographic predictor of structural productivity and performance sensitivities, respectively

Outcome	Predictor	df	Partial η²	F	P-value (uncorrected)	p (Holm)
**Effective productivity sensitivity**	age	1	0.000	0.001	0.981	1.000
	SES	1	0.000	0.016	0.899	1.000
	political orientation	1	0.069	8.420	0.004 **	0.027 *
	religion	1	0.004	0.430	0.514	1.000
	gender	1	0.008	0.944	0.333	1.000
	education	5	0.026	0.616	0.688	1.000
**Effective performance sensitivity**	age	1	0.000	0.048	0.827	1.000
	SES	1	0.016	1.900	0.171	1.000
	political orientation	1	0.005	0.615	0.434	1.000
	religion	1	0.005	0.578	0.449	1.000
	gender	1	0.015	1.690	0.197	1.000
	education	5	0.024	0.569	0.724	1.000

Note. Holm correction was applied separately within each outcome. Partial η² values are reported as measures of effect size.

Supplementary Table S19. Study 3 (*N*=125) Coefficient-level regression estimates for demographic predictors of sensitivity parameters

Outcome	Predictor	Estimate	Std_error	T_value	P-value (uncorrected)	P (Holm)
**Effective productivity sensitivity**	age	0.0002	0.012	0.024	0.981	1,000
	SES	0.012	0.092	0.127	0.899	1,000
	political_orientation	-0.217	0.075	-2.901	0.004 **	0.045
	religion	-0.052	0.080	-0.655	0.514	1,000
	genderMale	-0.269	0.277	-0.972	0.333	1,000
	educationDoctorate (e.g. PhD)	-0.320	1.081	-0.296	0.768	1,000
	educationHigh school degree or equivalent	0.013	0.330	0.038	0.970	1,000
	educationLess than high school diploma	-0.509	1.119	-0.455	0.650	1,000
	educationMaster's degree (e.g. MA / MSc / MEd)	0.061	0.408	0.149	0.882	1,000
	educationOther (please specify)	1.461	0.896	1.63	0.106	0.953
**Effective performance sensitivity**	age	0.002	0.008	0.219	0.827	1,000
	SES	0.085	0.061	1.378	0.171	1,000
	political_orientation	0.039	0.050	0.785	0.434	1,000
	religion	0.040	0.053	0.760	0.449	1,000
	genderMale	0.240	0.184	1.30	0.200	1,000
	educationDoctorate (e.g. PhD)	-0.689	0.720	-0.957	0.341	1,000
	educationHigh school degree or equivalent	0.011	0.210	0.049	0.961	1,000
	educationLess than high school diploma	-0.551	0.745	-0.740	0.461	1,000

	educationMaster's degree (e.g. MA / MSc / MEd)	-0.239	0.272	-0.8801	0.381	1,000
	educationOther (please specify)	-0.516	0.597	-0.864	0.389	1,000

(iii) Demographic Analyses and Observer Allocation: Study 4 (*N*=388)

In Study 4, we conducted exploratory socio-demographic analyses to examine whether individual differences in model parameters were associated with age, subjective socioeconomic status (SES), political orientation, religion, gender, or education.

We first fit a multivariate linear model with effective structural productivity sensitivity and performance sensitivity (1 - *gamma*) x *w* (‘*effective sensitivities*’) as joint outcomes. Using Pillai’s trace, political orientation showed an uncorrected association with the joint parameter profile, Pillai = .020, $F(2, 374) = 3.831$, $p = 0.022$, however, this association did not survive multiple-comparison correction, $p\_Holm = 0.135$. No other demographic predictor was reliably associated with the joint parameter profile. See **Supplementary Table S20**.

We then examined parameter-specific predictor-level tests through follow-up univariate analyses. For structural productivity sensitivity, political orientation showed a statistically significant uncorrected association, $F(1,375) = 4.46$, $p = 0.04$, $p\_Holm = 0.213$, partial $\eta^2 = 0.012$. Higher political-orientation scores were associated with lower sensitivity to structural productivity. No other demographic predictor was associated with structural productivity sensitivity after correction. For performance sensitivity, no demographic predictor showed a reliable association. See **Supplementary Table S21**.

Finally, coefficient-level follow-up tests were treated as descriptive and showed that political orientation negatively predicted structural productivity sensitivity, $b = -0.096$, $SE = 0.045$, $t = -2.11$, $p = 0.04$, $p\_Holm = 0.425$ within outcome. In contrast, political orientation did not predict effective performance sensitivity, $b = -0.0003$, $SE = 0.032$, $t = -0.012$, $p = 0.991$. See **Supplementary Table S22**.

Collectively, these exploratory analyses suggest that political orientation is the most consistent (uncorrected) demographic correlate of structural productivity sensitivity in Study 4, whereas age, SES, religion, gender, and education did not reliably explain individual differences in either sensitivity.

Supplementary Table S20. Study 4 (*N*=388) Exploratory MANOVA examining socio-demographics on jointly estimated parameters

Predictor	Pillai	F (df1, df2)	P-value (Uncorrected)	P (Holm)
**age**	0.004	0.708 (2, 374)	0.493	1.000
**SES**	0.009	1.707 (2, 374)	0.182	0.91
**political orientation**	0.020	3.832 (2, 374)	0.02 *	0.135
**religion**	0.004	0.804 (2, 374)	0.448	1.000
**gender**	0.008	0.525 (6, 750)	0.790	1.000
**education**	0.021	0.799 (10, 750)	0.630	1.000

Signif. codes: 0 '***' 0.001 '**' 0.01 '*' 0.05 '.' 0.1 ' ' 1

Supplementary Table S21. Study 4 (*N*=388) Exploratory univariate analyses examining demographic predictor of structural productivity and performance sensitivities, respectively

**Outcome**	**Predictor**	df	Partial η²	F	P-value (uncorrected)	p (Holm)
**Effective productivity sensitivity**	Age	1	0.002	0.763	0.383	1.000
	SES	1	0.000351	0.132	0.717	1.000
	Political orientation	1	0.0117	4.46	0.03 *	0.213
	Religion	1	0.00171	0.642	0.424	1.000
	Gender	3	0.00440	0.552	0.647	1.000
	Education	5	0.0105	0.796	0.553	1.000
**Effective performance sensitivity**	Age	1	0.000	0.038	0.845	0.991
	SES	1	0.001	0.543	0.461	0.991
	Political orientation	1	0.000	0.000	0.991	0.991
	Religion	1	0.002	0.785	0.376	0.991
	Gender	3	0.004	0.452	0.716	0.991
	Education	5	0.010	0.818	0.537	0.991

Note. Holm correction was applied separately within each outcome. Partial η² values are reported as measures of effect size.

Supplementary Table S22. Study 4 (*N*=388) Coefficient-level regression estimates for demographic predictors of sensitivity parameters

outcome	term	estimate	std_error	t_value	P-value (uncorrected)	P_holm
**Effective productivity sensitivity**	age	-0.005	0.006	-0.873	0.383	1
	SES	-0.017	0.047	-0.363	0.717	1
	political_orientation	-0.096	0.0454	-2.110	0.035 *	0.425
	religion	-0.040	0.050	-0.801	0.424	1
	genderMale	-0.040	0.152	-0.267	0.790	1
	genderOther	0.663	0.666	0.995	0.321	1
	genderPrefer not to say	-1.072	1.465	-0.732	0.465	1
	educationDoctorate (e.g. PhD)	-0.037	0.442	-0.085	0.932	1
	educationHigh school degree or equivalent	0.064	0.197	0.325	0.745	1
	educationLess than high school diploma	1.483	0.750	1.979	0.049 *	0.535
	educationMaster's degree (e.g. MA / MSc / MEd)	-0.003	0.208	-0.013	0.990	1
	educationOther (please specify)	0.067	0.332	0.207	0.836	1
**Effective performance sensitivity**	age	0.001	0.004	0.196	0.845	1
	SES	0.025	0.034	0.737	0.461	1
	political_orientation	-0.000	0.032	-0.011	0.990	1
	religion	-0.031	0.035	-0.886	0.376	1
	genderMale	-0.025	0.108	-0.235	0.814	1
	genderOther	-0.522	0.474	-1.102	0.271	1
	genderPrefer not to say	-0.394	1.043	-0.378	0.706	1

	educationDoctorate (e.g. PhD)	-0.366	0.314	-1.164	0.245	1
	educationHigh school degree or equivalent	0.048	0.140	0.344	0.731	1
	educationLess than high school diploma	-0.411	0.534	-0.770	0.442	1
	educationMaster's degree (e.g. MA / MSc / MEd)	0.137	0.148	0.925	0.356	1
	educationOther (please specify)	-0.144	0.236	-0.611	0.541	1
